# Direct copolymerization of ethylene with protic comonomers enabled by multinuclear Ni catalysts

**DOI:** 10.1038/s41467-021-26470-x

**Published:** 2021-11-01

**Authors:** Gang Ji, Zhou Chen, Xiao-Yan Wang, Xiao-Shan Ning, Chong-Jie Xu, Xing-Min Zhang, Wen-Jie Tao, Jun-Fang Li, Yanshan Gao, Qi Shen, Xiu-Li Sun, Hao-Yang Wang, Jun-Bo Zhao, Bo Zhang, Yin-Long Guo, Yanan Zhao, Jiajie Sun, Yi Luo, Yong Tang

**Affiliations:** 1 State Key Laboratory of Organometallic Chemistry, Shanghai Institute of Organic Chemistry, Chinese Academy of Sciences, Shanghai, China; 2 School of Chemistry and Chemical Engineering, Soochow University, Suzhou, China; 3 State Key Laboratory of Fine Chemicals, School of Chemical Engineering, Dalian University of Technology, Dalian, China; 4 Petrochina Petrochemical Research Institute, Beijing, China

**Keywords:** Environmental chemistry, Polymer chemistry

## Abstract

Ethylene/polar monomer coordination copolymerization offers an attractive way of making functionalized polyolefins. However, ethylene copolymerization with industrially relevant short chain length alkenoic acid remain a big challenge. Here we report the efficient direct copolymerization of ethylene with vinyl acetic acid by tetranuclear nickel complexes. The protic monomer can be extended to acrylic acid, allylacetic acid, ω-alkenoic acid, allyl alcohol, and homoallyl alcohol. Based on X-ray analysis of precatalysts, control experiments, solvent-assisted electrospray ionization-mass spectrometry detection of key catalytic intermediates, and density functional theory studies, we propose a possible mechanistic scenario that involves a distinctive vinyl acetic acid enchainment enabled by Ni···Ni synergistic effects. Inspired by the mechanistic insights, binuclear nickel catalysts are designed and proved much more efficient for the copolymerization of ethylene with vinyl acetic acid or acrylic acid, achieving the highest turnover frequencies so far for both ethylene and polar monomers simultaneously.

## Abstract (web summary)

The applications of polyolefins are limited by the nonpolar nature of the polymer. Here the authors show a method to copolymerize ethylene with the protic vinyl acetic acid via well defined multinuclear nickel catalysts.

## Introduction

Polyethylene (PE) is one of the most used plastic materials, which is in part due to its chemical stability/inertness. However, the non-polar feature also limits its further applications that require adhesiveness, compatibility, toughness, adhesion, surface properties (dyeability, printability, etc.) and rheological properties^1^. Industrial applications mainly rely on post-polymerization functionalization of polyolefins, which are usually under harsh conditions and lack selectivity due to the radical process that induces chain scission and cross-linking etc^2^. Ethylene coordination copolymerization with polar monomer is a straightforward option attracting much attention, but only limited success has been achieved so far. For example, group 4 transition metal catalysts have been very successful in olefin polymerizations, however, their use in polar monomer copolymerization is severely limited due to high catalyst oxophilicity^3–5^. Late transition metal catalysts^6–13^ such as Brookhart- ^9,13–16^, Grubbs- ^12,17^, Drent- ^11,18–20^, and Mitsubishi-type^21–23^ complexes, etc. exhibit greater polar comonomer tolerance and used in ethylene copolymerizations with a wide range of polar comonomers. But there is still a lack of catalyst systems with high efficiency and *M*_w_ capability in olefin-polar monomer copolymerization. Thus, developing new catalyst systems is still a great challenge that attracts considerable attention from both academia and industry^24^.

Even more challenging is to incorporate short chain length alkenoic acid, such as acrylic acid (AA) and vinyl acetic acid (VA) into PEs^25–27^, since: (*i*) acidic proton in comonomers such as alkenoic acids can protonate and thus poison the catalyst^27^; (ii) strong carboxylate coordination can promote the formation of stable chelate complex or β-X elimination^28,29^. Thus, although a few results of direct copolymerization of ethylene with AA using palladium catalyst based on either phosphinesulfonate or diphosphazane monoxide ligand were reported^25–27^, AA are more harmful to enchainment than the corresponding aprotic monomers in all the cases. For example, less than half AA enchainment was obtained comparing to methyl acrylate (MA) in ethylene copolymerization using phosphinesulfonate palladium under identical conditions^26^. As PEs containing -COOH groups are particularly important targets^30^, we are interested in the topic for several years^4,31,32^. Here we show a tetranuclear Ni catalyst system, which shows much higher efficiency in enchaining protic VA without compromising activity or *M*_w_ capability vs. its aprotic analog methyl vinyl acetate (MVA). The same trends are also seen for a range of protic monomers such as allylacetic acid, ω-alkenoic acid, allyl alcohol and homoallyl alcohol, exhibiting abnormal effects in contrast to previous catalyst systems. X-Ray analysis of precatalysts, control experiments, solvent-assisted electrospray ionization-mass spectrometry (SAESI-MS) detection of key intermediates, and DFT studies reveal a possible mechanistic scenario that involves a distinctive VA enchainment enabled by Ni···Ni synergistic effects. Based on the mechanistic insights, a binuclear nickel catalyst is further designed and proves much more efficient for the copolymerization of ethylene with either VA or AA, achieving the highest TOFs so far for both ethylene and polar monomers simultaneously^25–27^.

## Results and Discussion

### Synthesis and characterization of tetranuclear nickel complexes

**Fig. 1:**
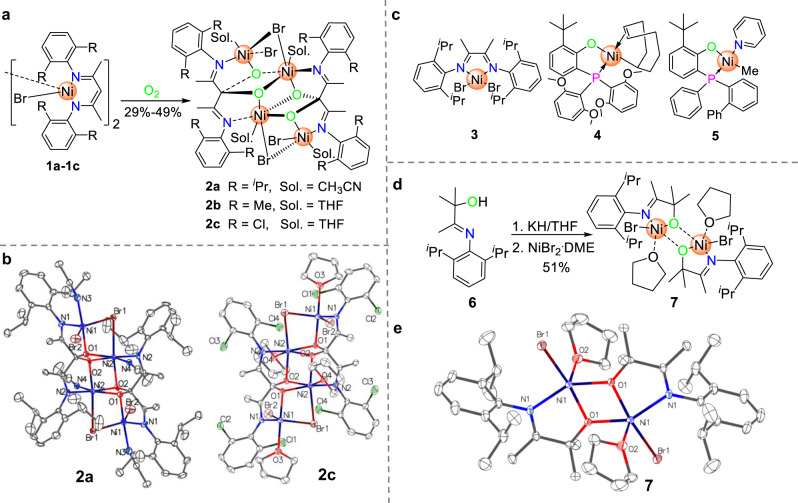
Structure formula and single crystal structures of catalysts. **a** Synthesis of tetranuclear nickel complexes **2a**-**2c**. **b** Single crystal structures of complexes **2a** and **2c**. **c** Literature reported nickel complexes **3**, **4** and **5**. **d** Synthesis of complex **7**. **e** Single crystal structure of complex **7**.

Complexes **1a-c** were synthesized in good yields. Bubbling O_2_^33^ into acetonitrile or toluene solution of **1a-c** at 40 ^o^C leads to solution color changes from blue to red or brown (Supplementary Methods 1.2). After recrystallization from CH_3_CN/*n*-hexane or THF/*n*-hexane solution, pure **2a-c** were obtained and characterized by Elem. Anal. and further confirmed by X-ray analysis (Fig. 1a, b). As shown in Fig. 1b, both **2a** and **2c** are tetranuclear nickel clusters composed of two oxidized ligands. The Ni1···Ni2 distances for **2a** and **2c** are 3.331 Å and 3.324 Å, respectively, which are shorter than the sum of van der Waals radii of two nickel atoms (4.44 Å)^34^. These are among one of the shortest Ni···Ni distances in bimetallic Ni complexes for olefin polymerization such as by Marks (~3.1 Å)^35^, Takeuchi (4.73 Å)^36^, which show great Ni···Ni synergistic effects. The Ni1-O1 bond lengths are 1.991(5) and 2.002(3) Å, and the Ni2-O1 bond lengths are 2.041(5) and 2.056(3) Å for **2a** and **2c**, respectively, which are slightly longer than that in salicylaldiminato Ni(II) complex (1.910 Å)^10^, suggesting similar Ni-O bond strengths/robustness even for the shared O in **2**.

### Ethylene polymerization

Upon activation with modified methylaluminoxane (MMAO)^37^, the conversion of mononuclear **1a-c** into tetranuclear **2a-c** change them from inactive catalysts into very active ones (Table 1, entries 1-3 vs. 4-6). For example, **2a** promotes ethylene polymerization with activity as high as 2928 kg/(mol Cat)∙h∙atm, and high *M*_w_, 850 kg/mol (entry 4). Replacing ^*i*^Pr group on aniline by methyl group (**2b**) or Cl (**2c**) results in slightly decreased activity and PE *M*_w_ (entries 5-6).

**Table 1:** Ethylene polymerizations catalyzed by Ni catalysts **1**, **2** and **7**.

Entry^a^	Cat. (µmol)	Yield (g)	Act.^b^	TOF^c^	*M*_w_^d^ (kg/mol)	Đ^d^	B^e^	Chains/Cat.	*T*_m_^f^ (^o^C)
1	**1a** (2.5)	trace	-	-	-	-	-	-	-
2	**1b** (2.5)	trace	-	-	-	-	-	-	-
3	**1c** (2.5)	trace	-	-	-	-	-	-	-
4	**2a** (1.25)	0.61	2928	104571	850	2.4	22	1.4	108
5	**2b** (1.25)	0.25	1200	42857	439	4.6	22	2.1	109
6	**2c** (1.25)	0.18	864	30857	566	2.4	19	0.6	110
7	**2a** (0.25)	0.24	5760	205714	904	2.1	18	2.2	109
8 ^g^	**7** (2.5)	none	-	-	-	-	-	-	-

^a^Conditions: toluene, 100 mL; MMAO, 1500 equiv./Ni; ethylene, 1 atm; 30 °C, 10 min; each entry performed in duplicate. ^b^kg/(mol Cat.)∙h∙atm. ^c^In units of mol_E_/mol_Cat._∙h∙atm. ^d^by GPC. ^e^Total branches/1000 C, by ^1^H NMR. ^f^By DSC. ^g^Neither PE nor oligomers (by GC) detected in the presence of MMAO, MAO, or Et_2_AlCl.

### Ethylene + polar monomer copolymerization

Precatalysts **2a-c** are used for ethylene + polar monomer copolymerizations following three different procedures (Table 2, Supplementary Methods 1.3). Procedure A: polar monomer is mixed with MMAO in toluene under ethylene for 5 min before **2** is introduced to start polymerization; Procedure B: polar monomer is pretreated with MMAO for 24 h before **2** is introduced under ethylene to start polymerization without additional MMAO; Procedure C: polar monomer is pretreated with 1.2 equiv. R_3_Al (R = Me, TMA; R = Et, TEA; R = ^*i*^Bu, TIBA) for 24 h before MMAO and **2** are introduced under ethylene to start polymerization.

**Table 2:** Copolymerization of ethylene and various polar monomers.


Ent.^a^	Cat. (µmol)	Comon. (M)	Pretreatment procedure& reagent	Yield (g)	Act.^b^	TOF^c^ (E/comon.)	*M*_w_^d^	Đ^d^	Incorp.^e^ (mol%)	B^f^	Chains /Cat.	*T*_c_^g^ (^o^C)
1	**2a** (10.0)	VA (0.3)	A, -	0.09	54	1807/40	33	3.3	2.1	76	1.0	79
2^h^	**2a** (10.0)	VA (0.3)	A, -	0.08	48	1616/32	22	3.0	1.9	54	1.0	79
3	**2b** (10.0)	VA (0.3)	A, -	0.03	18	610/11	25	2.7	1.7	68	0.4	76
4	**2c** (10.0)	VA (0.3)	A, -	0.02	12	398/10	26	2.9	2.4	74	0.3	74
5	**2a** (10.0)	VA (0.3)	B, MMAO	0.14	84	2965/11	102	3.6	0.4	76	0.5	99
6	**2a** (10.0)	VA (0.3)	C, TMA	0.12	72	2526/15	53	5.4	0.6	56	1.2	91
7	**2a** (10.0)	VA (0.3)	C, TEA	0.13	78	2749/12	47	6.1	0.4	69	1.7	92
8	**2a** (10.0)	VA (0.3)	C, TIBA	0.19	114	4018/17	33	3.6	0.4	55	2.1	92
9	**2a** (10.0)	MVA(0.3)	A, -	0.11	66	2267/25	15	3.0	1.1	79	2.1	81
10^i^	**2a** (5.0)	PA (0.15)	A, -	0.17	408	11949/733	31	3.8	5.8	75	4.3	39
11^i^	**2a** (5.0)	PA (0.15)	C, TMA	0.28	672	23168/233	122	2.6	1.0	38	1.2	82
12^i^	**2a** (1.25)	UA (0.2)	A. -	0.14	1344	23235/3763	n.d.	n.d.	13.9	24	-	30
13^i^	**2a** (1.25)	UA (0.2)	C, TIBA	0.15	1440	43522/1201	n.d.	n.d.	2.7	27	-	61
14^i^	**2a** (2.5)	UA (0.4)	A, -	0.12	576	7345/2010	n.d.	n.d.	21.5	25	-	n.d.
15^j^	**2a** (5.0)	HAA(0.2)	A, -	0.21	252	8399/233	87	2.9	2.7	49	1.4	70
16^j^	**2a** (15.0)	HAA(0.2)	A, -	0.37	148	4623/257	39	3.2	5.3	60	2.0	55
17^j^	**2a** (5.0)	HAA(0.2)	C, TMA	0.80	960	33174/432	261	4.0	1.3	32	2.5	86
18^k^	**2a** (25.0)	A-ol (0.4)	A, -	0.10	24	826/15	8	3.2	1.8	86	1.6	59
19^k^	**2a** (25.0)	AME(0.4)	A, -	0.10	24	856/0.3	14	3.2	0.04	69	0.9	91
20^k^	**2a** (25.0)	AA (0.4)	A, -	0.12	29	1020/3	21	5.1	0.3	81	1.2	70
21	**3** (40.0)	VA (0.3)	A, -	trace	-	-	-	-	-	-	-	-
22	**3** (40.0)	VA (0.3)	C, TMA	0.81	122	4313/9	62	2.0	0.2	118	0.7	n.d.
23 ^m^	**4**(10.0)	AA(0.1)	-, -	none	-	-	-	-	-	-	-	-
24^n^	**4**(10.0)	AA(1.5)	A, DEAC	trace	-	-	-	-	-	-	-	-
25^p^	**5**(10.0)	AA(0.2)	-, -	trace	-	-	-	-	-	-	-	-
26^n^	**5**(10.0)	AA(1.5)	A, DEAC	trace	-	-	-	-	-	-	-	-

^a^Conditions: toluene, 50 mL; MMAO, 90 mmol (Al/Ni = 2250); ethylene, 1 atm; 30 °C, 10 min; each entry performed in duplicate. ^b^kg/(mol Cat)∙h∙atm; ^c^In units of mol_E_/mol_Cat_.∙h∙atm; mol_Comon._/mol_Cat_.∙h∙atm. E, ethylene. ^d^By GPC, kg/mol. ^e^By ^1^H NMR. ^f^Total branches/1000 C, by ^1^H NMR. Note that branches due to polar monomer enchainment are not counted. ^g^By DSC. ^h^MMAO, 30 mmol. ^i^MMAO (Al/Ni = 3000), 5 min. ^j^MMAO, 60 mmol; ^k^MMAO, 90 mmol (Al/Ni = 900). ^m^20 atm ethylene pressure, 60 min, 70 °C. ^n^AA was in situ mixed with 75 mmol DEAC before polymerization, 3 atm ethylene pressure, 30 min, 30 °C. ^p^10 atm ethylene pressure, 60 min, 50 °C.

Following Procedure A, i.e., no polar monomer pretreatment, precatalyst **2a** catalyzes ethylene + vinylacetic acid (VA) copolymerization with an activity of 54 kg/(mol Cat)∙h∙atm and great VA incorporation (2.1 mol%, entry 1). VA incorporation is confirmed by a combined NMR (^1^H, ^13^C, COSY, HSQC, HMBC) and ATR-IR characterizations (Supplementary Note 1 and Supplementary Fig. 17). Unexpectedly, following Procedure B, i.e., mixing VA with MMAO for 24 h, results in a marginal increase of activity to 84 kg/(mol Cat)∙h∙atm with a significant 5.3× decrease in VA incorporation to 0.4 mol% (entry 5 vs. 1). This trend is counterintuitive as pretreating polar monomers with aluminoxane or R_3_Al is an effective strategy for group 4^3,4,32^ and late^38,39^ transition metal complexes catalyzed polar monomer copolymerizations. It is further confirmed by control experiments following Procedure C, in which the copolymerizations with **2a** only enchain ~0.5 mol% VA regardless of the type of R_3_Al used (entries 6-8). We also tried corresponding aprotic methyl vinylacetate (MVA) following Procedure A, in which comparable activity is obtained but with obviously reduced comonomer incorporation (1.1 mol% vs. 2.1 mol%) and *M*_w_ (15 kg/mol vs. 33 kg/mol) (entry 9 vs. 1). These intriguing trends likely suggest the abnormal effects of -COOH, in contrast to previous catalyst systems^11,40^, showing the unique character of **2** in maintaining good activity and enhancing protic monomer enchainment when Procedure A is followed. In contrast, the well-established α-diimine NiBr_2_ **3** (Fig. 1c) only gives good activity in ethylene + VA copolymerization by pretreating VA with TMA (Procedure C) but shows negligible activity following Procedure A (entry 21 vs. 22). While neutral o-bis(aryl)-phosphinophenolate nickel catalysts **4**^23^ and **5**^21^ (Fig. 1c) are known for good tolerance toward polar monomers^21,22^, these catalysts show negligible activity following Procedure A or typical literature conditions (entries 23-26 and Supplementary Table 3). Thus, these results further demonstrate the difference of cluster catalyst **2** from the known Ni catalysts.

Concurred with the trend observed in **2a** catalyzed ethylene copolymerization with protic monomer VA, i.e., enhanced comonomer incorporation, there is also a similar trend for both 4-pentenoic acid (PA) and 9-undecenoic acid (UA) following Procedure A vs. Procedure C, 5.8 vs. 1.0 mol% for PA (entry 10 vs. 11) and 13.9 vs. 2.7 mol% (entry 12 vs. 13) for UA. In addition, alkenol monomers can also be efficiently incorporated in **2a** catalyzed ethylene copolymerizations. For example, higher HAA incorporation is achieved following Procedure A vs. Procedure C (2.7 vs. 1.3 mol%, entry 15 vs. 17); and the incorporation of allylic alcohol (A-ol) is much higher than its aprotic analog AME (1.8 mol% vs. 0.04 mol%, entry 18 vs. 19). Thus, the above abnormal effects that protic monomer is incorporated much more efficiently than the corresponding aprotic monomers are extensively observed in **2a**-catalyzed ethylene copolymerization with protic polar monomers.

### Rationale for the abnormal effects

To get insights into the effects, we first designed ligand **6** and tried to synthesize mononuclear nickel complex with similar coordination environment to complex **2** to investigate whether it is a ligand effect or a Ni···Ni synergistic effect. The complex **7** shows very similar Ni-O and Ni-N bond parameters to **2a** and **2c** (Fig. 1d, e). However, **7** is inert in ethylene polymerization (Table 1, entry 8). This result suggests less ligand effect and that there may be a substantial synergism between the neighboring nickels during the copolymerization process.

**Fig. 2:**
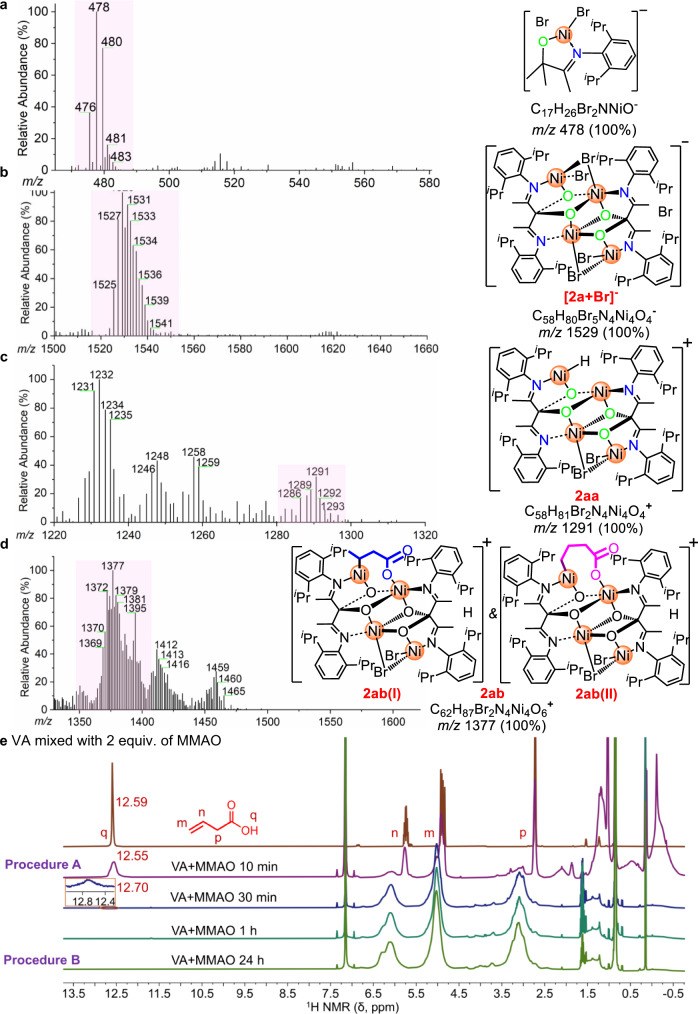
SAESI-MS and NMR spectra. **a** SAESI-MS spectra of **7** in DCM showing signals at m/z 478 [(0.5)**7** + Br]^-^. **b** SAESI-MS spectra of **2a** showing signals of [**2a** + Br]^-^. **c** SAESI-MS spectra of **2a**/MMAO showing signals of [**2a**-2Br + H]^+^ (**2aa**). **d** SAESI-MS spectra of **2a**/MMAO/VA showing signals of [**2a**-2Br + H + VA]^+^ (**2ab**). **e** ^1^H NMR tracking reaction mimicking polar monomer pretreatment procedures (in C_6_D_6_, 25 ^o^C).

To further understand the Ni···Ni synergistic effects^41^, a recently developed solvent-assisted electrospray ionization-mass spectrometry (SAESI-MS) technique was used for straightforward capture of the species^42^. With SAESI-MS, we observed a strong peak that corresponds to [**2a** + Br]^-^ in dichloromethane (DCM), suggesting the cluster nickel complex **2a** does not collapse in DCM (Fig. 2b). In contrast, only a signal at m/z 478 was observed in **7**/DCM, suggesting the dimer collapses into monomer (Fig. 2a). Furthermore, a major signal at m/z 1291 was captured when **2a** was in situ mixed with MMAO (Al/Ni = 25, mimicking the polymerization conditions in Supplementary Table 1, entry 5), which is likely attributed to species [**2a**-2Br + H]^+^ (**2aa**) by reasonable inference (Fig. 2c). **2aa** is likely formed via facile MMAO alkylation and then β-H elimination during **2a** activation^37^. We next explored the polar monomer enchainment step by SAESI-MS. When **2a**, VA and MMAO were mixed in situ (mimicking Procedure A), an intense signal at m/z 1377 was observed (Fig. 2d). This signal could be reasonably deduced as species (**2ab**). However, only the signal at m/z 1291 corresponding to [**2a**-2Br + H]^+^, instead of the chelating species **2ab** ([**2a**-2Br + H + VA]^+^, m/z 1377), was observed when VA was pretreated with MMAO for 24 h before contacting **2a** (Procedure B, Supplementary Fig. 3). The formation of **2ab**(I) and **2ab**(II) due to 2,1- or 1,2-insertion of VA into the Ni-H is consistent with the results of control experiment (Supplementary Methods 1.7, Supplementary Table 2). These results suggest the catalytic species is very likely still a nickel cluster and the aforementioned abnormal effects are associated with Ni···Ni synergetic effects.

To further understand the different results of SAESI-MS spectra, we monitored the reaction of MMAO + VA with a 2/1 molar ratio by ^1^H NMR (the same MMAO/VA molar ratio as in Table 2, entry 2). As shown in Fig. 2e, nearly 50% free VA is intact after mixing for 10 min (Procedure A). The peak at 12.55 ppm was observed even after 30 min. and gradually disappeared in 1 h (Procedure B). The existence of active proton under Procedure A-type condition and the lack thereof under Procedure B-type condition suggests the proton might play a significant role in the activation procedure-associated abnormal effects in **2** catalyzed ethylene + VA copolymerization.

**Fig. 3:**
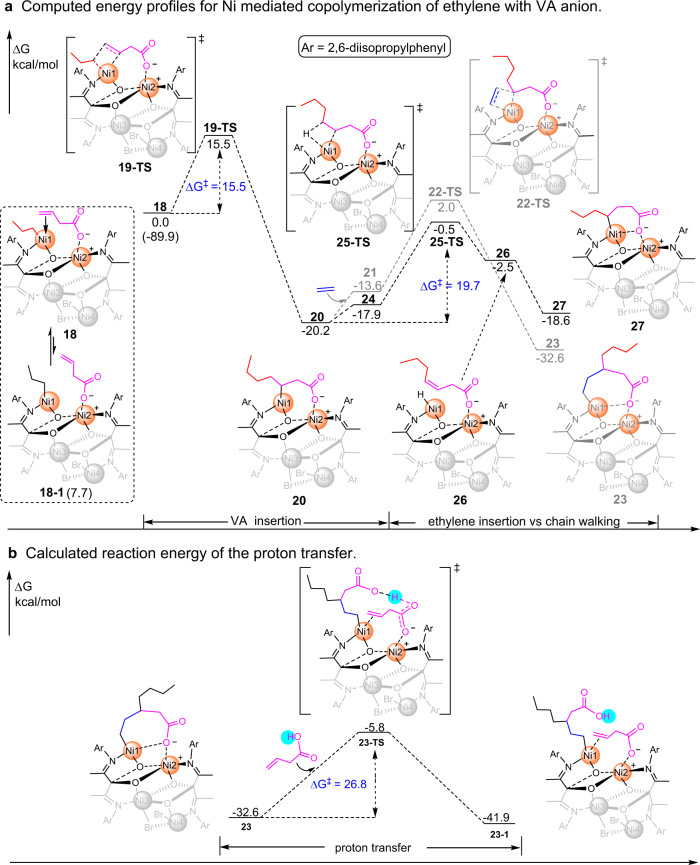
DFT calculations. **a** Computed energy profiles (M06(SMD)/6-311 + G** ∩ SDD//B3LYP/6-31 G* ∩ LANL2DZ, the solvent used in calculations is toluene) for Ni mediated copolymerization of ethylene with VA anion (the effect of large aluminum counterion was not considered). The energies are relative to the intermediate **18**. **b** Calculated reaction energy (M06(SMD)/6-311 + G** ∩ SDD//B3LYP/6-31 G* ∩ LANL2DZ, the solvent used in calculations is toluene) of the proton transfer. The energies are relative to the intermediate **18**.

To clarify the Ni···Ni synergistic effects^43^ and the role of proton in the copolymerization of ethylene with protic polar comonomers, we carried out DFT calculations. From a practical point of view, the growing polymer chain was modeled by an *n*-propyl group, the insertion product of the first ethylene insertion^44^. Initially, the computed energy difference between **18** and **18-1** (ΔΔG = − 7.7 kcal/mol) shows that simultaneous coordination of anionic vinyl acetate via its O atom to Ni2 and its C = C bond to Ni1 center is more favorable (Fig. 3a). The interaction between polar group and metal center^36,45–49^ changes the polar monomer coordination from an intermolecular to an intramolecular process. Further insertion of such intramolecularly coordinated olefin occurs at the center of Ni1 with an energy barrier of ΔG^‡^ = 15.5 kcal/mol (**19-TS**), yielding a seven-membered ring chelate product **20**. This step is dramatically exergonic by ΔG = − 20.2 kcal/mol. These results suggest that the bimetallic cooperative VA coordination/insertion is both kinetically and thermodynamically feasible. The seven-membered ring chelate product **20** readily undergoes ethylene coordination/insertion giving intermediate **23** with an energy barrier of ΔG^‡^ = 22.2 kcal/mol and exergonic by ΔG = −12.4 kcal/mol. Alternatively, **20** could also undergo chain-walking. In this case, the β-H elimination is more kinetically favorable than ethylene insertion (energy barrier of 19.7 vs. 22.2 kcal/mol) (Fig. 3a). Furthermore, the re-insertion of the resulting Ni−H bond in **26** to give **27** is also exergonic. Though the re-insertion transition state was not located, this step should be kinetically accessible^50^. The results agree with the SAESI-MS studies which identified a strong signal that corresponds to **2ab** in the in situ mixed **2a**/MMAO/VA system and suggest the formation of copolymers with extended polar branches (Fig. 2d). This is consistent with the microstructural analysis of the copolymers by a combined NMR experiments (^1^H, ^13^C, HSQC, and HMBC), in which the –COOH groups are observed at the end of branches with varied branch lengths (Supplementary Note 1 and Supplementary Fig. 17).

In this proposed mechanistic scenario, the release of the –COONi from catalytic metal center is one of the key steps. DFT studies show that the proton could assist the release of the coordinating carboxyl group and thus allow further polar monomer enchainment in the copolymerization. As shown in Fig. 3b, the metallocycle in the intermediates such as **23**, formed via Ni···Ni synergistic effects, eventually releases the coordinating carboxyl group via the proton transfer in the polymerization system, which circumvent chain termination and ensures multiple and efficient polar monomer enchainment. DFT calculations do show such a process is both thermodynamically and kinetically feasible (∆G = −9.3 kcal/mol; ∆G^‡^ = 26.8 kcal/mol). Combining the aforementioned results of ^1^H NMR studies, this explains well the observations that copolymerizations following Procedure A afford greater VA enchainment than those following Procedures B and C.

### Binuclear nickel catalyst and the copolymerization of ethylene with VA or AA

**Fig. 4:**
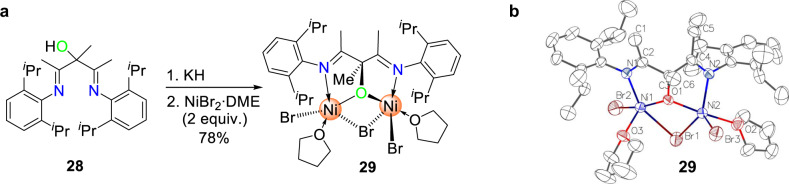
Synthesis and structure of **29**. **a** Synthesis of complex **29**. **b** Single crystal structure of complex **29**.

The above studies show that tetranuclear cluster **2** could greatly enhance the enchainment of various protic monomers vs. aprotic analogs. We are glad to observe similar trends in catalysis with binuclear complex **29**, which has a shorter Ni···Ni distance than those of **2a** and **2c** (3.259 Å vs. ~3.33 Å) (Fig. 4). Moreover, **29** gave both higher activities and higher incorporation of AA or VA compared with complexes **2**, in particular for the industrially more relevant AA monomer (Table 3). For example, upon activation with Et_2_AlCl (DEAC), **29** gave an activity of 300 kg/(mol Cat.)∙h with 2.6 mol% AA incorporation—8.7× higher than that of **2a** (Table 3, entry 5 vs. Table 2, entry 20). Similar protic acid comonomer preference (VA and AA) vs. aprotic MVA and MA was also observed (entry 1 vs. 4; entry 5 vs. 6). Notably, **29** exhibits good thermal stability over 30 min even at 90 °C under 10 atm ethylene (entry 9). Thus, binuclear complex **29** achieved the highest TOFs for the copolymerization of ethylene with VA and AA^25–27^.

**Table 3:** Ethylene/AA (or VA) copolymerization catalyzed by precatalyst **29**.

Ent.^a^	Pre. (atm)	T (°C)	t (min)	Comon. (M)	Pretreatment procedure &reagent^b^	Yield (g)	Act.^c^	TOF^d^ (E/comon.)	*M*_w_^e^ (kg/mol)	Đ^e^	Incorp.^f^ (mol%)	B.^g^	*T*_m_^h^ (°C)
1	1	30	5	VA(0.4)	A, –	0.45	540	17306/644	13	2.7	3.6	74	6(*T*_c_)
2	1	30	5	VA(0.4)	B, DEAC	0.87	1044	35692/518	71	2.9	1.4	82	25(*T*_c_)
3	1	30	5	VA(0.4)	C, TIBA	0.39	468	16441/89	206	3.5	0.5	78	24(*T*_c_)
4	1	30	5	MVA(0.4)	A, –	0.26	312	10976/47	72	1.6	0.4	67	41(*T*_c_)
5	1	30	5	AA(0.4)	A, –	0.25	300	10013/272	1.5	2.9	2.6	35	15(*T*_c_)
6	1	30	5	MA(0.4)	A, –	0.24	288	10160/41	22	2.8	0.4	57	n.d.
7^i^	3	30	30	AA(1.5)	A, –	1.32	264	8947/187	10	3.1	2.1	44	102
8^i^	3	30	30	AA(1.5)	B, –	1.28	256	8869/106	14	3.3	1.2	36	100
9^i^	10	90	30	AA(1.5)	A, –	0.29	58	1974/38	13	2.4	1.9	59	84

^a^Conditions: toluene, 50 mL; Cat. **29** (10 µmol dissolved in 5 mL toluene), DEAC (3.0 M in toluene), 30 mmol. ^b^Procedures A-C are similar to those in Table 2 except that MMAO is replaced with DEAC. ^c^kg/(mol_Cat._)∙h. ^d^In units of mol_E_/mol_Cat_.∙h; mol_Comon._/mol_Cat_.∙h. E, ethylene. ^e^By GPC. ^f^By ^1^H NMR. ^g^By ^1^H NMR. ^h^By DSC. ^i^DEAC, 75 mmol.

In summary, tetranuclear nickel complexes **2** prove successful precatalysts for direct copolymerization of ethylene with protic comonomers with high activity and incorporation. In contrast to known systems, **2** show much higher efficiency in enchaining VA without compromising activity or *M*_w_ capability vs. its aprotic analog MVA. This distinctive protic polar monomer preference is also observed for a range of other protic polar monomers such as allylacetic acid, ω-alkenoic acid, allyl alcohol, and homoallyl alcohol, etc. SAESI-MS captured the signal of [**2a**-2Br + H + VA]^+^ in mimicking the copolymerization procedure, suggesting the active species is likely still tetranuclear nickel species. ^1^H NMR studies on the mixed VA + MMAO show proton exists within 30 min, corroborating with all polymerizations results, suggesting the important role the proton might play in the copolymerization. Based on the experimental results and DFT calculations, a rationale involving enchainment of VA enabled by distinctive Ni···Ni synergistic effects is proposed. Inspired by the mechanistic insights, the designed binuclear precatalyst **29** with shorter Ni···Ni distance is synthesized, which is much more efficient for the copolymerization of ethylene with VA and AA than tetranuclear complexes **2**. Thus, we developed a potentially useful catalyst to enchain the industry-relevant monomers AA and VA into PE backbone, achieving the highest TOFs for the copolymerization of ethylene with VA and AA. This finding shows multinuclear catalysis as an effective strategy in direct coordination copolymerization of ethylene and protic comonomer and paves a way for the design of catalyst in the synthesis of functionalized PEs.

## Methods

### General Information

For synthetic procedures, NMR spectra of compounds, NMR, GPC, and ATR-IR spectra of polymers, see Supplementary Methods and Supplementary Figs. 14–36. For details on SAESI-MS studies of key species, see Supplementary Methods 1.6 and Supplementary Figs. 1–5. Model experiments to determine the insertion mode of polar monomers are given in Supplementary Methods 1.7 and Supplementary Figs. 6–8. NMR study of VA and butyric acid (BA) mixed with MMAO is shown in Supplementary Methods 1.8 and Supplementary Fig. 9. Computational studies can be found in Supplementary Note 4 and Supplementary Figs. 10–13. Polar copolymer microstructure analysis is given in Supplementary Note 1. Branching analysis from ^1^H NMR and ^13^C NMR and polar monomer incorporation analysis from ^1^H NMR are outlined in Supplementary Note 2 and 3.

## Supplementary information

Supplementary Information

## Data Availability

Experimental procedures and characterizations, including detailed experimental procedures, NMR spectra, complete polymerization experiment information, analysis of the polymer structure, and computational details are available in the Supplementary Information. The data that support the plots within this paper are available from the corresponding authors upon reasonable request. The X-ray crystallographic coordinates for structures reported in this study have been deposited at the Cambridge Crystallographic Data Centre (CCDC), under deposition numbers CCDC1575003 (**2a**), CCDC1575004 (**2c**), CCDC1575009 (**7**) and CCDC206294229 (**29**). These data can be obtained free of charge from The Cambridge Crystallographic Data Centre via www.ccdc.cam.ac.uk/data_request/cif.
