# Supplementary material for: Direct copolymerization of ethylene with protic comonomers enabled by multinuclear Ni catalysts
Source: Nat Commun. 2021 Nov 1;12:6283. doi: 10.1038/s41467-021-26470-x (PMC8560877; doi:10.1038/s41467-021-26470-x)
Supplement: Supplementary file 1 — Supplementary Information [file 41467_2021_26470_MOESM1_ESM.pdf]

Direct copolymerization of ethylene with protic comonomers enabled by multinuclear Ni catalysts

Gang Ji, Zhou Chen, Xiao-Yan Wang, Xiao-Shan Ning, Chong-Jie Xu, Xing-Min Zhang, Wen-Jie Tao, Jun-Fang Li, Yan-Shan Gao, Qi, Shen, Xiu-Li Sun,* Hao-Yang Wang, Jun-Bo Zhao, Bo Zhang, Yin-Long Guo,* Yanan Zhao, Jiajie Sun, Yi Luo* and Yong Tang*

*Correspondence to: tangy@sioc.ac.cn, xlsun@sioc.ac.cn, ylguo@sioc.ac.cn, luoyi@dlut.edu.cn

Supplementary Information

Supplementary Methods 2

1.1 General Considerations 2

1.2 Synthesis of Compounds 3

1.3 General Polar Monomer Pretreatment Procedures 7

1.4 General Procedure for Ethylene Polymerization 7

1.5 General Procedures for Ethylene + Polar Monomer Copolymerization 7

1.6 SAESI-MS Studies of Key Species/Intermediates 9

1.7 Model Experiments to Determine the Insertion Mode of Polar Monomers: Reaction of **2a**/MAO with VA 11

1.8 NMR Study of VA and Butyric Acid (BA) Mixed with MMAO 12

1.9 Methyl Esterification Procedures for Ethylene/Alkenoic Acids Copolymers 12

Supplementary Note 1 13

Supplementary Note 2 14

Supplementary Note 3 15

Supplementary Note 4 16

Supplementary Tables 19

Supplementary Figures 31

Supplementary References 60

Supplementary Methods

1.1 General Considerations

All air and moisture sensitive manipulations were carried out under high purity N_2 using standard Schlenk technique or in a glovebox. ^{1}H NMR, ^{13}C NMR, DEPT 135, COSY, HSQC and HMBC spectra were recorded on Agilent Technologies 400 MHz spectrometer, Varian 400 MHz spectrometer or Agilent Technologies 600 MHz spectrometer. Elemental analysis was performed by the Analytical Laboratory of Shanghai Institute of Organic Chemistry (CAS). M_n, M_w, and dispersity (Đ) were determined with Agilent Technologies PL-GPC 220 High temperature Gel Permeation Chromatography at 150 °C (polystyrene calibration, 1,2,4-trichlorobenzene as solvent at 150°C). X-Ray crystallographic data was collected using a Bruker AXSD8 X-ray diffractometer. Mass spectra were carried out with a HP5989A spectrometer. SAESI-MS spectra were recorded on a Thermo TSQ Quantum Access triple-quadrupole mass spectrometer (ThermoFisher Scientific, Waltham, MA). SAESI conditions: vacuum, 1.8×10^{-6} torr; spray voltage, ±3500 V; capillary temperature, 270 °C; sheath gas pressure, 5 psi; aux valve flow, 2 arbitrary unit; collision energy of CID, 35 eV. Data acquisition and analysis were done with the Xcalibur (version 2.0, Thermo Fisher Scientific) software package. GC-MS spectra were recorded on a Shimadzu GCMS-QP2010 Plus equipped with Shimadzu AOC-5000 multi-function autosampler, and the operating software is MDGC solution. The chromatographic conditions: DB-5MS capillary column (30 m × 0.25 mm i.d × 0.25 μm); inlet temperature 250 °C, split injection (10:1), carrier gas helium (purity > 99.999%), flow rate 1.0 mL/min; temperature program: start 50 °C, hold 3 min, then increase to 300 °C with 10 °C/min, hold 2 min, the whole process is 30 min, injection volume: 1 μL. Mass spectrometry conditions: the transmission line temperature 280 °C, the ion source temperature 250 °C, the electron bombardment energy 70 eV, m/z range 40 to 500, the solvent delay 2 min.

Toluene, hexane, CH_3CN, dichloromethane (DCM) and tetrahydrofuran (THF) were purified by MBraun SPS-800 system. Pentane was dried over Sodium still and distilled immediately before use. Methylaluminoxane (MAO) and modified methylaluminoxane (MMAO) were purchased from AkzoNobel as 1.5 M toluene solution and 2.0 M heptane solution, respectively, and used as received. Polymerization-grade ethylene was purified by going through ethylene purification system (R&D by Dalian Institute of Chemical Physics, CAS). Trimethylaluminum (TMA),

triethylaluminum (TEA), triisobutylaluminium (TIBA) and diethylaluminum chloride (DEAC) were purchased from Jiangsu Yongjian Chemical Co., Ltd as neat liquid and used as received. Ligands **L1a**, **L1b**, **L1c** and complexes **1a** and **1b** were synthesized according to literature[1-2]. The *o*-bis(aryl)-phosphinophenolate nickel catalysts **4** and **5** were prepared according to literature[3-4].

1.2 Synthesis of Compounds

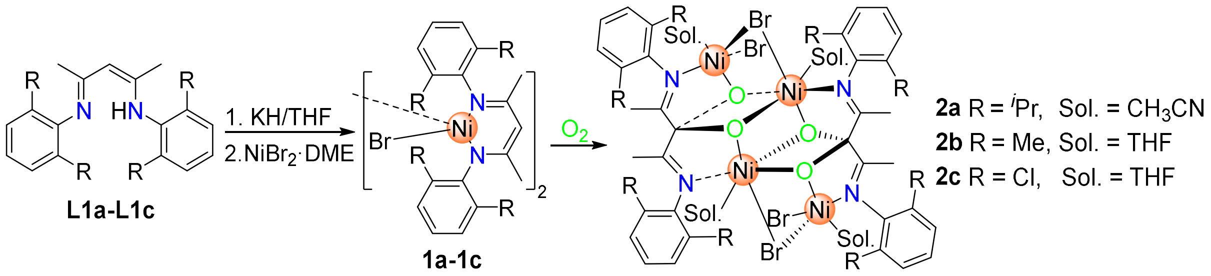

Synthesis of Complex **1c**

To a suspension of potassium hydride (KH) (0.056 g, 1.4 mmol) in 20 mL dry THF was added a solution of **L1c** (0.388 g, 1.0 mmol) in THF (15 mL) at 25 °C. The resulting suspension was stirred overnight. After filtration, the solution was added dropwise into a suspension of $NiBr_2 \cdot DME$ (0.309 g, 1.0 mmol) in THF (10 mL) at 25 °C and the suspension was stirred for 12 h. All volatiles were removed under vacuum, and the residue was dissolved in dry toluene (30 mL). After filtration, the filtrate was concentrated to 2 mL and dry pentane (20 mL) was added. The precipitated solid was washed twice by pentane and dried under vacuum to give blue solid, 0.342 g, 65% isolated yield. Anal. Calcd. for $C_{17}H_{13}BrCl_4N_2Ni$: C, 38.84; H, 2.49; N, 5.33. Found: C, 38.84; H, 2.44; N, 5.15.

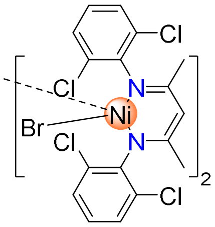

1c

Synthesis of Complexes **2**

Complex **1** (2.0 mmol) dissolved in acetonitrile or toluene (60 mL) was stirred under O_2 at 40 °C for 1 week. The solution color turned from blue to red or brown over time. After the solution was concentrated to 2 mL, hexane (10 mL) was added to precipitate yellow solid. Pure **2a** was

obtained by recrystallization from acetonitrile; **2b** and **2c** were obtained by recrystallization from a mixed solvent of THF and hexane.

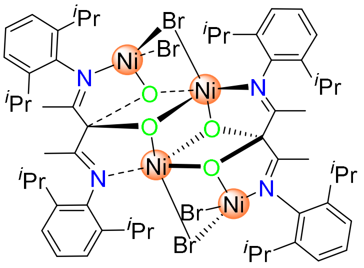

2a

The product was obtained by recrystallization from acetonitrile. Yield: 0.17 g (49 %). Anal. Calcd. for $C_{58}H_{80}Br_4N_4Ni_4O_4$: C, 47.99; H, 5.55; N, 3.86. Found: C, 47.98; H, 5.70; N, 3.63.

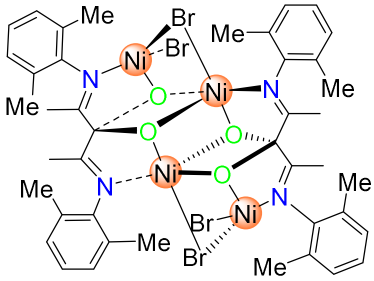

2b

The product was obtained by recrystallization from THF/hexane solution (2 mL/4 mL). Yield: 0.06 g (29 %). Anal. Calcd. for $C_{42}H_{48}Br_4N_4Ni_4O_4$: C, 41.10; H, 3.94; N, 4.57. Found: C, 41.49; H, 4.36; N, 4.56.

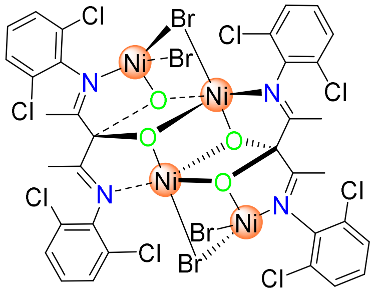

2c

The product was obtained by recrystallization from THF solution. Yield 0.47 g (31 %). Anal. Calcd. for $C_{34}H_{24}Br_4Cl_8N_4Ni_4O_4$: C, 29.37; H, 1.74; N, 4.03. Found: C, 29.78; H, 1.98; N, 4.20.

Synthesis of Complex **7**

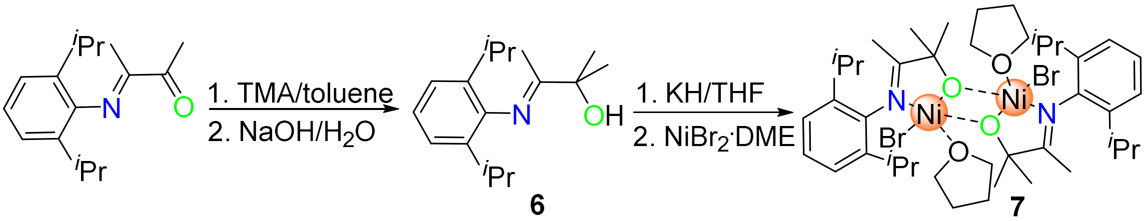

Under N_2, to (2,6-diisopropylphenylimido)-2-butene-3-one[5] (10.41 g, 42.44 mmol) in toluene (90 mL) was slowly added TMA (50 mL, 1.0 M) at room temperature. The reaction mixture was refluxed for 4 h, then carefully quenched with 5% NaOH aqueous solution at 0 ºC. The organic layer was extracted with ethyl acetate, dried over $MgSO_4$. After all volatiles were removed, the resulting red oil was purified by flash chromatography (petroleum ether/ethyl acetate, 30/1,v/v), giving **6** as pale yellow solid. Yield 5.56 g (50 %). ^{1}H NMR (400 MHz, $CDCl_3$): δ = 7.15-7.09 (m, 3H, Ar-*H*), 5.67 (brs , 1H, O*H*), 2.65 (m, 2H, C*H*(CH_3)$_2$), 1.76 (s, 3H, C*H*$_3$), 1.48 (s, 6H, C*H*$_3$), 1.14 (d, J = 6.8Hz,12H, C*H*$_3$); ^{13}C-NMR (100 MHz, $CDCl_3$): δ = 175.7, 144.1, 136.6, 124.1, 123.1, 73.0, 27.9, 27.6, 23.2, 22.9, 15.2; HRMS(ESI): [M+H]$^+$ calcd for $C_{17}H_{28}NO$, 262.2171; Found, 262.2168. Anal. Calcd. for $C_{17}H_{27}NO$: C, 78.11; H, 10.41; N, 5.36. Found: C, 78.11; H, 10.61; N, 5.36.

To the suspension of potassium hydride (KH) (0.19 g, 4.8 mmol) in dry THF (30 mL) was added a solution of **6** (1.05 g, 4.0 mmol) in THF (10 mL) at 25 °C, and the suspension was stirred overnight. The resulting solution was then added dropwise into a suspension of $NiBr_2$·DME (1.23 g, 4 mmol) in THF (10 mL) and the resulting mixture was stirred for 12 h. Volatiles were removed under reduced pressure, and the residue was dissolved in dry toluene (30 mL). After filtration, the filtrate was concentrated to 5 mL before 15 mL pentane was added, the resulting precipitated solid was washed twice by hexane and dried under vacuum to give **7** as sky-blue solid. Yield: 0.96 g (51 %). Anal. Calcd. for $C_{17}H_{26}BrNNiO·(C_4H_8O)$: C, 53.54; H, 7.27; N, 2.97. Found: C, 53.92; H, 6.87; N, 2.75. Heating **7** under vacuum at 300-400 °C removes THF with the color turning gray. Anal. Calcd. for $C_{17}H_{26}BrNNiO$: C, 51.17; H, 6.57; N, 3.51. Found: C, 51.25; H, 6.72; N, 3.48.

Synthesis of Ligand **28**

α-Keto-β-diimine complex was synthesized according to the reported literature[6-7], and the ligand **28** was synthesized as follows.

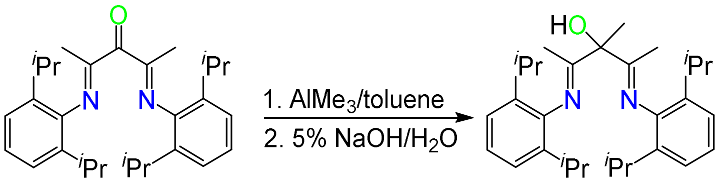

Under N_2, to α-keto-β-diimine complex (7.22 g, 16.69 mmol) in toluene (60 mL) was added TMA (20 mL, 1.0 M, 1.2 equiv.) slowly at room temperature. The reaction mixture was refluxed for 10 h, then the resulting mixture was hydrolyzed carefully with 5% aqueous NaOH solution at 0 ºC. The organic product was extracted with ethyl acetate, dried over $MgSO_4$. After removing the solvent, the resulting slightly yellow solid was purified by recrystallization from a mixture of petroleum ether/ethyl acetate (10/1, v/v), affording **28** as colorless crystal. Yield 6.7 g (90%). ^{1}H NMR (400 MHz, $CDCl_3$): δ = 7.18-7.09 (m, 6H, Ar-*H*), 6.29 (brs, 1H,O*H*), 2.79-2.65 (m, 4H, C*H*(CH_3)$_2$), 1.86 (s, 6H, C*H*$_3$), 1.83 (s, 3H, C*H*$_3$), 1.19-1.16 (m, 24H, C*H*$_3$); ^{13}C NMR (100 MHz, $CDCl_3$): δ = 172.8, 144.8, 136.2, 136.0, 123.9, 123.1, 80.5, 28.1, 24.4, 23.4, 23.3, 22.9, 22.7, 15.9; HRMS(ESI): [M+H]$^+$ calcd for $C_{30}H_{45}N_2O$,449.3532; Found, 449.3524. Anal. Calcd. for $C_{30}H_{44}N_2O$: C, 80.31; H, 9.88; N, 6.24. Found: C, 80.22; H, 9.64; N, 6.16.

Synthesis of Complex **29**

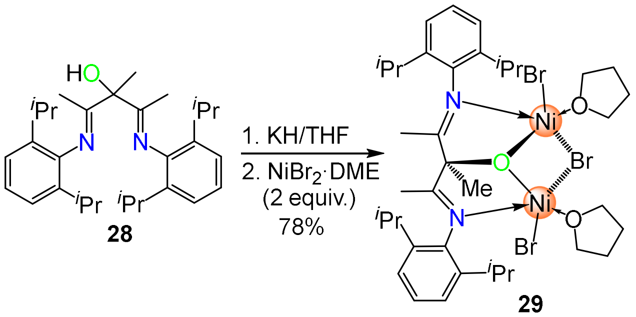

To a suspension of potassium hydride (KH) (0.12 g, 3.0 mmol, 2.0 equiv.) in dry THF (20 mL) was added a solution of **28** (0.67 g, 1.5 mmol) in THF (15 mL) at 25 °C. The resulting suspension was stirred overnight. It was then added dropwise into a suspension of $NiBr_2$·DME (0.93 g, 3.0 mmol, 2.0 equiv.) in THF (10 mL) at 25 °C and the suspension was stirred for 12 h. All volatiles were removed under reduced pressure and the residue was dissolved in dry DCM (30 mL). After filtration, the filtrate was removed under reduced pressure and the precipitated solid was dissolved in THF for crystallization. The final product **29** was obtained as red solid, 1.12 g, yield: 78%. The single crystals were grown from a mixed solvent of THF/hexane (2.0 mL/1.0 mL,

v/v). Anal. Calcd. for $C_{30}H_{43}Br_3N_2Ni_2O$ (solvent-free): C, 44.77; H, 5.39; N, 3.48. Found: C, 44.79; H, 5.60; N, 3.21.

1.3 General Polar Monomer Pretreatment Procedures

Procedure A: Under 1 atm ethylene, polar monomer was added dropwise into toluene solution of cocatalyst (MMAO or DEAC). After 10 min, desired amount of Ni catalyst in toluene was added to start polymerization.

Procedure B: To a solution of desired amount of VA or AA in toluene (10 mL) at -78 ^oC was added desired amount of cocatalysts (MMAO or DEAC). Additional toluene was added till the total volume is 45 mL. The resulting mixture was allowed to warm to room temperature and stirred for 24 h under N_2 before use.

Procedure C: To a solution of desired amount of polar monomer (AA, VA, PA, UA or HAA) in toluene (10 mL) at -78 ^oC was added R_3Al (1.2 equiv.) in toluene (5 mL). The resulting mixture was allowed to warm to room temperature. Then desired amount of toluene was added until the total volume of the solution is 20 mL and the resulting solution was stored under N_2 for 24 h before use.

1.4 General Procedure for Ethylene Polymerization

To a desired amount of toluene saturated with ethylene at the desired temperature, the desired amount of cocatalyst was added. The mixture was stirred for 10 min and the polymerization was started by adding toluene solution of nickel catalyst. After the desired time, the polymerization was quenched with acidified ethanol and then the mixture was poured into acidified ethanol (300 mL, 10 % HCl (v/v) in ethanol). The precipitated polymer was collected, washed with ethanol, and then dried under vacuum at 60 °C to constant weight.

When complex **1** or **7** was used as precatalyst, the polymerization procedures was the same as described above. However, the reaction bottle was quickly sealed and put under dry ice acetone bath at the end of polymerization. An aliquot was filtered using NNY 0.45 μm membrane and was then quickly injected into GC detector for the analysis of possible oligomer products.

1.5 General Procedures for Ethylene + Polar Monomer Copolymerization

a) Copolymerization of ethylene and polar monomer following *Procedure A*:

Under 1 atm ethylene:

Under 1 atm ethylene, to a solution of toluene and desired amount of cocatalyst (MMAO or DEAC) was added dropwise the polar monomer. In all cases, the total volume was kept at 45 mL. At the desired temperature, desired amount of Ni catalyst in toluene (5 mL) was added to start polymerization. The reaction was quenched with acidified methanol (concentrated HCl/MeOH, 1/20, v/v). Then the resulting mixture was poured into 300 mL acidified methanol (concentrated HCl/MeOH, 1/20, v/v) and kept stirring for 2 hours. The precipitated polymer was collected, washed with methanol, and dried under vacuum at 60 °C to constant weight.

Under elevated ethylene pressure:

Polymerizations were carried out in a Parr reactor. The Parr reactor was dried under vacuum at 120 °C for 6 h and cooled to desired temperature. It was then pressurized with ethylene and vented, and repeated 3 times. Under ethylene flow, dry toluene and desired amount of cocatalyst (MMAO or DEAC) was injected. Then desired amount of AA was added dropwise. At the desired temperature, desired amount of Ni catalyst in toluene was added. Then, the autoclave was quickly sealed and pressurized to desired pressure. The reaction mixture was stirred under constant ethylene pressure for the given reaction time, and then the pressure was carefully released. After rapidly cooling the autoclave to room temperature, the reaction was quenched with acidified methanol (concentrated HCl/MeOH, 1/20, v/v). The resulting mixture was poured into 300 mL acidified methanol (concentrated HCl/MeOH, 1/20, v/v) and kept stirring over night. The precipitated polymer was collected, washed with methanol, and dried under vacuum at 60 °C to constant weight.

b) Copolymerization of ethylene and polar monomers following *Procedure B*:

Under 1 atm ethylene, desired amount of toluene was added to the solution of cocatalyst-pretreated polar monomer while keeping the total volume as 45 mL. Then, desired amount of Ni catalysts (in 5 mL toluene) was added to start polymerization (*Note*: no additional cocatalyst added). The reaction was quenched with acidified methanol (concentrated HCl/MeOH, 1/20, v/v). Then the resulting mixture was poured into 300 mL acidified methanol (concentrated HCl/MeOH, 1/20, v/v) and kept stirring for 2 hours. The precipitated polymer was collected, washed with methanol, and dried under vacuum at 60 °C to constant weight.

c) Copolymerization of ethylene and polar monomers following *Procedure C*:

To toluene solution of R_3Al-pretreated polar monomer was added the desired amount of cocatalyst (MMAO or DEAC) under 1 atm ethylene, followed by desired amount of toluene to make the total volume at 45 mL. At the desired temperature, desired amount of Ni catalyst in 5

mL toluene was added to start polymerization. The reaction was quenched with acidified methanol (concentrated HCl/MeOH, 1/20, v/v). Then the resulting mixture was poured into 300 mL acidified methanol (concentrated HCl/MeOH, 1/20, v/v) and kept stirring for 2 hours. The precipitated polymer was collected, washed with methanol, and dried under vacuum at 60 °C to constant weight.

1.6 SAESI-MS Studies of Key Species/Intermediates

Firstly, complex **2a** (2.4 mg, 1.65 μmol) was dissolved in DCM (3 mL). Then the resulting solution was filtered through NNY 0.45 μm membrane before SAESI-MS analysis. Complex **2a** is soluble in DCM, but DCM is not compatible with ESI-MS analysis due to its low conductivity. Solvent-assisted electrospray ionization-mass spectrometry (SAESI-MS) method uses the apparatus shown in Supplementary Figure 1, recently developed by Guo *et al*[8], successfully solved the issue. SAEIS-MS method uses two separate sprayers intersecting at the spray end tip, which allows for the simultaneous use of both ESI-friendly solvent such as CH_3CN and DCM to dissolve Ni complex.

In the SAESI-MS experiment, the angle (α) between the two sprayers is 45 degree and the distance (L) between the tip of sprayers and the inlet to the mass is 6 mm[8]. The solution of catalyst were injected by a 500-μL air-tight syringe with a speed at 10 μL/min to SAESI-MS. The solution of MAO or MMAO was injected by another 500-μL air-tight syringe at a speed of 6 μL/min to SAESI-MS. *Note*: after ESI-MS measurements the ion source has to be thoroughly cleaned immediately to remove metal residue.

a) Structure of complex 7 in DCM

Complex **7** (2.0 mg) dissolved in DCM (4 mL) was injected into the ESI-MS detector, An ESI-MS spectrum with a clear signal at *m/z* 478 was observed. It suggests complex **7** collapses into its monomer in DCM (Figure 2a)

b) Structure of complex 2a in DCM

Complex **2a** (2.4 mg, 1.65 μmol) dissolved in DCM (3 mL) was injected into the ESI-MS detector, A clear ESI-MS spectrum of **2a** obtained showing the strong signals of $[M+Br]^-$ at *m/z* 1529, indicating the existence of intact **2a** in the solvent (Figure. 2b).

c) Detection of active intermediate in 2a/MMAO system

In the SAESI-MS positive ion mode, **2a** (2.4 mg, 1.65 μmol) dissolved in DCM (3 mL) and MMAO (165 μmol, Al/Ni = 25) in toluene (15 mL) were injected through two separate channels simultaneously. Once formed, the active intermediate can be captured by SAESI-MS. A clear ESI-MS spectrum of **2a/MMAO** was obtained showing the strong signals of $[\mathbf{2a}\text{-2Br+H}]^+$ at *m/z* 1291 (Figure. 2c), which can be attributed to species **2aa** by reasonable inference[9, 10-13].

Towards the active intermediate at *m/z* 1291, MS/MS spectrometry was performed giving the following spectrum. It further confirms the structure of **2aa** (Supplementary Figure 1).

d) Analysis of active species in 2a/MMAO/VA system

VA (83 μmol, 7μL) (VA/**2a** = 50, MMAO/VA = 2) was mixed with 2 equiv. of MMAO (165 μmol, 82 μL, 2.0 M in heptane) in toluene (15 mL). The solution of complex **2a** (1.65 μmol in 3 mL DCM) and the *in situ* mixed solution of MMAO/VA were injected with two separated sprayers at the same time. In the SAESI-MS positive ion mode, a clear ESI-MS spectrum of **2a**/MMAO/VA shows strong signals of $[\mathbf{2a}\text{-2Br+H+VA}]^+$ at *m/z* 1377 (Figure. 2d), which correspond to species **2ab**.

MS/MS spectrometry was performed towards *m/z* 1377, further confirming the structure of **2ab** (Supplementary Figure 2).

e) MMAO/VA mixed for 24 h before ESI-MS analysis

7 μL VA (83 μmol) (VA/**2a** = 50, Al/VA = 2) was pretreated with 2 equiv. of MMAO (165 μmol, 82 μL, 2.0 M in heptane). The resulting solution was diluted in toluene (15 mL), and stirred at room temperature for 24 h before ESI-MS experiment.

The solution of complex **2a** (1.65 μmol in 3 mL DCM) and the resulting MMAO/VA mixture were injected with two separated sprayers at the same time. In the SAESI-MS positive ion mode, no signal was detected at *m/z* 1377 (Supplementary Figure 3) .

f) ESI-MS analysis for 2a/MMAO/MVA system

7.5 μL MVA (83 μmol) (MVA/**2a** = 50, Al/MVA = 2) was pretreated with 2 equiv. of MMAO (165 μmol, 82 μL, 2.0 M in heptane), and the resulting solution was diluted in toluene (15 mL). The solution of complex **2a** (1.65 μmol in 3 mL DCM) and the *in situ* mixed solution of MMAO/MVA were injected with two separated sprayers at the same time. In the SAESI-MS positive ion mode, no signal related to the chelation complex of **2a** and MVA was captured (Supplementary Figure 4a). Instead, signal of MVA at m/z 101 was detected (Supplementary Figure 4b).

g) Detection of active intermediate in 2a/MAO system

In the SAESI-MS positive ion mode, **2a** (2.4 mg, 1.65 μmol) dissolved in DCM (3 mL) and MAO (165 μmol, Al/Ni = 25) in toluene (15 mL) were injected through two separate channels at the same time. A clear ESI-MS spectrum of **2a/MAO** was obtained with the signal of [**2a**-2Br+Me]$^+$ at *m/z* 1305 (Supplementary Figure 5).

1.7 Model Experiments to Determine the Insertion Mode of Polar Monomers: Reaction of 2a/MAO with VA

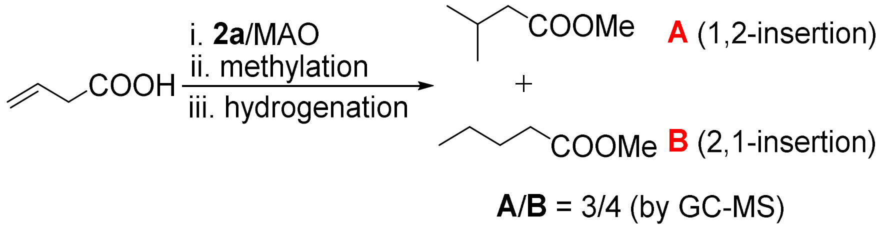

VA monomer insertion into Ni-Me was first studied using MMAO. However, the alkylated species likely undergo facile β-H elimination giving Ni-H species, which complicates the insertion regiochemistry studies. Instead, when MAO was used, the alkylated species contains Ni-Me moiety was detected by SAEIS-MS (Supplementary Figure 5).

a) Product separation

Under Ar, VA (0.232 mmol) in toluene (14.5 mL) was added dropwise to MAO (1.5 M in toluene, 15.5 mL) (23.3 mmol, 25 equiv./Ni). **2a** (0.232 mmol) in toluene (20 mL) was then added and the reaction mixture was stirred for 30 minutes at 30 °C. Finally, the reaction was quenched with saturated potassium sodium tartrate, extracted with DCM, and the aqueous layer was kept and further acidified with HCl (12.0 M) until pH = 1.

b) Methyl esterification

Then, the aqueous solution was extracted with 3 × 100 mL DCM, and the combined organic extract was dried under vacuum. The residue was then dissolved in 10 mL methanol and 3-4 drops concentrated sulfuric acid (H_2SO_4) was added. The resulting mixture was refluxed for two hours for complete esterification (monitored by ^{1}H NMR), and then anhydrous sodium bicarbonate ($NaHCO_3$) was added to neutralize the acid. The resulting reaction mixture was filtered through a thin layer of silica gel, and all volatiles were removed to give the crude product as red oil.

d) Hydrogenation

The methyl esterification product was dissolved in methanol (15 mL), and Pd/C (15 mg of 10 wt%) was added. The reaction mixture was sealed in an autoclave, and the reactor was pressurized with hydrogen and vented, and was repeated 3 times before it was pressurized to 15 bar. The reaction mixture was stirred under 60 °C for 24 h for complete olefin hydrogenation. After pressure was carefully released, Pd/C was removed by filtering through celite. All volatiles were then removed under vacuum to give the crude hydrogenated product as red oil.

e) GC-MS analysis

The crude hydrogenated product was analyzed by GC-MS. Methyl pentanoate and methyl isopentanoate were both detected as the major products (Supplementary Figures 6-8 and Supplementary Table 2).

1.8 NMR Study of VA and Butyric Acid (BA) Mixed with MMAO

In order to understand the difference between *in situ* mixed VA/MMAO and 24 h VA/MMAO or R_3Al pretreatment, we carried out NMR studies. In a glovebox, 150 μL MMAO (2.0 M in heptane, 0.3 mmol, 2 equiv.) in 10 mL Schlenk tube was connected to a vacuum system to evaporate the solvent. Then 0.6 mL benzene-d_6 was added and the deuterated solvent containing dry MMAO was transferred to an NMR tube. Subsequently, 12.7 μL VA (0.15 mmol) was added into the NMR tube, NMR data was recorded after 10 min, 30 min, 1 hour and 24 hours (Figure 2e).

In a glovebox, 150 μL MMAO (2.0 M in heptane, 0.3 mmol, 2 equiv.) in 10 mL Schlenk tube was connected to a vacuum system to evaporate the solvent. Later, 0.6 mL benzene-d_6 was added and the deuterated solvent containing dry MMAO was transferred to an NMR tube. Subsequently, 13.7 μL butyric acid (BA) (0.15 mmol) was added into the NMR tube, NMR data was recorded after 10 min, 30 min, 1 hour and 24 hours (Supplementary Figure 9).

1.9 Methyl Esterification Procedure for Ethylene/Alkenoic Acid Copolymers

At the end of the ethylene/alkenoic acid copolymerization, acidified methanol (concentrated HCl/MeOH, 1/20, v/v) was added to quench the reaction. For this reason, both carboxylic acid and methyl ester group exist in the copolymer (Supplementary Figure 35a). In order to obtain a clear NMR spectrum for subsequent polymer microstructural analysis, methyl esterification was carried out to convert all carboxylic acid group to methyl ester group. The ethylene/alkenoic acid copolymer was added into a 100 mL flask containing 60 mL 1,2-dichlorobenzene solution with

0.03 g butylated hydroxytoluene (BHT) as an antioxidant. Subsequent, 5 mL methanol and 3-4 drops concentrated sulfuric acid (H_2SO_4) was added before the solution was heated under 120 °C for 2 hours. Finally, the hot solution was poured into 100 mL methanol to precipitate the copolymer. The copolymers was further detected by ATR-IR spectra featuring a prominent ν(C=O) in ester group at 1745 cm^{-1} (Supplementary Figure 35b).

Supplementary Note 1

Polar Copolymer Microstructure Analysis

A representative quantitative ^{13}C NMR spectrum of ethylene/VA copolymer (Table 2, entry 1) is shown in Supplementary Figure 17b. According to 2D NMR spectrum analysis (Supplementary Figure 17), the resulting copolymer exhibits a structure as shown in the following scheme, the signals appear at 39.72, 32.36 and 34.66 ppm can be assigned to the α-carbons adjacent to carbonyl groups, representing three different structures **I**, **II**, and **III**, respectively. Microstructure **I** is mainly generated from direct VA insertion into polymer chain followed by ethylene insertion. Microstructure **II** is formed via VA insertion into polymer chain followed by one-step chain walking process and further ethylene insertion. Microstructure **III**[14] is likely generated from VA insertion into polymer chain followed by multi-step chain walking process and further ethylene insertion (*path 1*) and/or is formed by a quick chain termination reaction after 2,1-insertion of VA (*path 2*). Although we can't rule out the possibility of path 2, DFT studies suggest *path 1* is preferred (see DFT calculation results in Figure. 3a).

Besides, from the above microstructure analysis of enchained polar monomer, we didn't observe product with chain end resulting from 1,2-insertion. Generally, the regioselectivity of C=C bond insertion into metal-alkyl bond usually follows the following pattern: electron-deficient olefins selectively insert in a 2,1-fashion, whereas electron-rich olefins insert in a 1,2-fashion[15]. Quantitative ^{13}C NMR spectrum shows that the copolymer has 76 branches/1000 C, including 3.2% branch-on-branch (*sec*-butyl) structure and 22.7% longer chain (butyl+) branch structure, indicative of extensive chain walking relative to propagation[16,17]. Besides, for the esterified copolymers of ethylene with VA (Table 2, entry 1), ATR-IR spectra feature a prominent ν(C=O) in ester group at 1745 cm^{-1} (Supplementary Figure 35).

NMR assignments for the ethylene/VA copolymers (after methyl esterification) with chemical shifts listed in ppm:

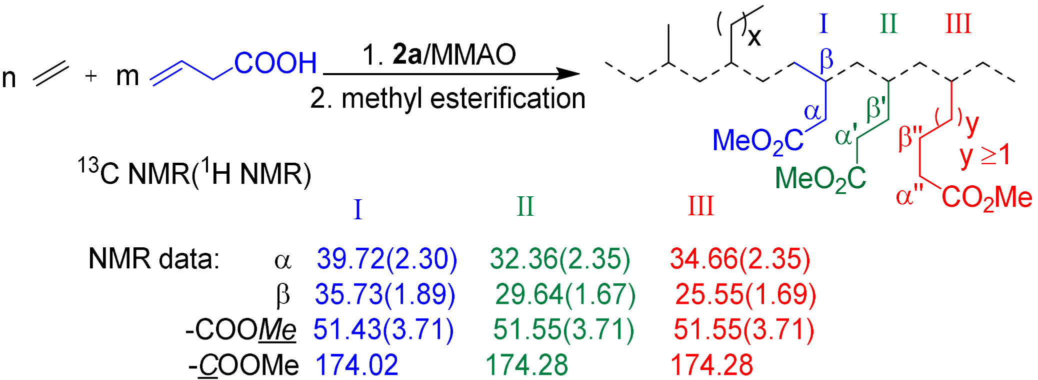

^{13}C NMR(^{1}H NMR)

		I	II	III
NMR data:	α	39.72(2.30)	32.36(2.35)	34.66(2.35)
	β	35.73(1.89)	29.64(1.67)	25.55(1.69)
	-COO*Me*	51.43(3.71)	51.55(3.71)	51.55(3.71)
	-*C*OOMe	174.02	174.28	174.28

A representative quantitative ^{13}C NMR spectrum of ethylene/HAA copolymer (Table 2, entry 16) is shown in Supplementary Figure 21b. According to literature[18] and 2D NMR spectra analysis (Supplementary Figure 21), the resulting copolymer exhibits a structure as shown in the following scheme, the signals appear at 61.89, 63.95 and 63.50 ppm can be assigned to the methylene carbons adjacent to hydroxyl respectively, representing three different structures **I**, **II**, and **III**.

NMR assignments for the ethylene/HAA copolymers with chemical shifts listed in parts per million:

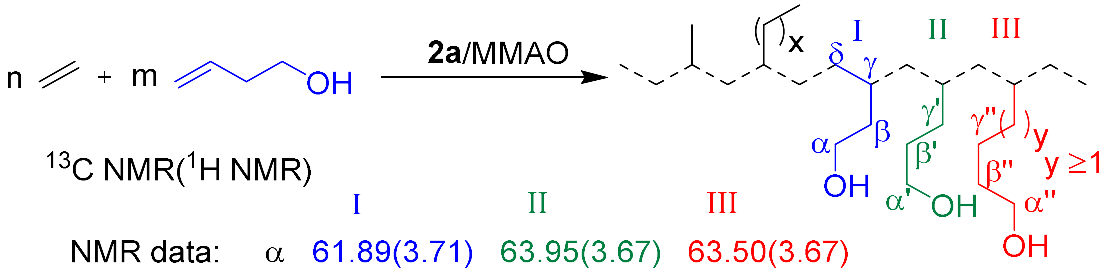

^{13}C NMR(^{1}H NMR)

		I	II	III
NMR data:	α	61.89(3.71)	63.95(3.67)	63.50(3.67)
	β	38.14(1.60)	33.69(1.62)	30.98(1.63)
	γ	35.50	23.89	26.30
	δ	34.88	-	-

Supplementary Note 2

Branching Analysis from ^{1}H NMR and ^{13}C NMR.

The amount of methyl groups ($N_{Me\ groups/1000C}$) and the amount of total branches ($N_{Branches/1000C}$) based on ^{1}H NMR spectra were analyzed according to literature[17]. Since neither internal nor terminal double bonds was observed in the ^{1}H NMR spectra, M_n could not be accurately assessed by NMR. Finally, The M_n data used in $N_{Branches/1000C}$ equation was from GPC analysis ($N_{Me\ groups/1000C}$ was corrected with two methyl groups for each polymer chain).

$$N_{Me\ groups/1000C} = 2\times I_{Me}/(3\times I_{tot})\times 1000$$

$$N_{\text{Branches/1000C}} = \left[\frac{N_{\text{Me groups/1000C}}}{1000} \cdot \frac{M_{\text{n}}}{14\ \text{g mol}^{-1}} - 2\right] \cdot \frac{1000}{M_{\text{n}}} \cdot 14\ \text{g mol}^{-1}$$

As described above, total branch density is calculated by ^{1}H NMR. The percentages of various branches such as methyl (Me), ethyl (Et), propyl (*n*-Pr), linear branches ≥C4 (Bu^{+}) and secondary butyl (*sec*-Bu) are calculated from ^{13}C NMR spectra based on well-established examples[17,19]. Note that the branches from polar monomer enchainment are not counted.

Supplementary Note 3

Polar Monomer Incorporation Analysis from ^{1}H NMR.

The insertion ratio of VA was calculated from quantitative ^{1}H NMR spectrum analysis of copolymers after methyl esterification. The mole ratios of VA and ethylene in the copolymer are x and 1-x, respectively. The integration at chemical shift δ = 1.0 ~ 1.5 ppm is set as n, and the integration at δ = 2.25 ~ 2.45 ppm is set as 1.

$$\frac{2x}{2x + 4(1 - x)} = \frac{1}{n}$$

$$x\ \text{mol\%} = \frac{2}{n + 1} \times 100\%$$

In the case of ethylene/AA copolymerization under elevated ethylene pressure (Table 3, entries 7-9), The COOH group was located at both in-chain and chain-end position for the copolymers (Supplementary Figure 25). Thus, the accurate calculation of AA incorporation ratio was challenging. Considering the in-chain AA structure was less than half of the total AA incorporation, the insertion ratio of AA was proximately calculated as the formula for ethylene/VA coploymers.

The insertion ratio of PA was calculated from quantitative ^{1}H NMR spectrum analysis of copolymers. The mole ratios of PA and ethylene in the copolymer are x and 1-x, respectively. The integration at chemical shift δ = 1.0 ~ 1.5 ppm is set as n, and the integration at δ = 2.25 ~ 2.45 ppm is set as 1.

$$\frac{2x}{3x + 4(1 - x)} = \frac{1}{n}$$

$$x\ \text{mol\%} = \frac{4}{2n + 1} \times 100\%$$

The insertion ratio of UA was calculated from quantitative ^{1}H NMR spectrum analysis of copolymers. The mole ratios of UA and ethylene in the copolymer are x and 1-x, respectively. The

integration at chemical shift δ = 1.0 ~ 1.5 ppm is set as n, and the integration at δ = 2.25 ~ 2.45 ppm is set as 1.

$$\frac{2x}{15x + 4(1 - x)} = \frac{1}{n}$$

$$x \text{ mol\%} = \frac{4}{2n - 11} \times 100\%$$

The HAA incorporation ratio was calculated from quantitative ^{1}H NMR spectrum analysis[20]. The mole ratios of HAA and ethylene in the copolymer are x and 1-x, respectively. The integration at chemical shift δ = 1.0 ~ 1.5 ppm is set as n, and the integration at δ = 3.65 ~ 3.75 ppm is set as 1.

$$\frac{2x}{3x + 4(1 - x)} = \frac{1}{n}$$

$$x \text{ mol\%} = \frac{4}{2n + 1} \times 100\%$$

The A-ol incorporation ratio was calculated from quantitative ^{1}H NMR spectrum analysis. The mole ratios of A-ol and ethylene in the copolymer are x and 1-x, respectively. The integration at chemical shift δ = 1.0 ~ 1.5 ppm is set as n, and the integration at δ = 3.65 ~ 3.75 ppm is set as 1.

$$\frac{2x}{2x + 4(1 - x)} = \frac{1}{n}$$

$$x \text{ mol\%} = \frac{2}{n + 1} \times 100\%$$

Supplementary Note 4

Computational Studies

All calculations were performed with the Gaussian 16 program[21] The B3LYP hybrid exchange-correlation functional was utilized for geometry optimization[22-24]. Each optimized structure was subsequently analyzed by harmonic vibration frequencies for characterization of a minimum (Nimag = 0) or a transition state (Nimag = 1) and providing thermodynamic data. The transition state structures are shown to connect the reactant and product on either side via intrinsic reaction coordinate (IRC) following. The 6-31G(d) basis set was considered for C, H, O, N atoms. The Ni and Br atoms were treated by LANL2DZ and the associated basis sets[25-27]. This basis set is denoted as “BSI”. To obtain more reliable relative energies, the single-point calculations of optimized structures were carried out at the level of M06[28-29]/BSII, taking into account solvation

effect of toluene with the SMD[30] solvation model. In the BSII, the 6-311+G(d,p) basis set was used for nonmetal atoms, while the base set SDD[31-33] as well as associated pseudopotentials are used for Ni and Br atoms. Therefore, unless otherwise mentioned, the free energy (ΔG, 298.15 K, 1 atm) in solution, which was used for description of energy profiles, was obtained from the solvation single-point calculation and the gas-phase Gibbs free energy correction.

The polymerization experiment shows obvious "chain walking" phenomena in the tetranuclear Ni catalyst system as the resulting polymer has many branches, suggesting a favorable β-H elimination after ethylene insertion. Besides, it is computationally found that the β-H elimination and subsequent reinsertion have energy barriers of only 7.8 and 6.1 kcal/mol, respectively. However, ethylene insertion surmounts energy barriers of 8.6 kcal/mol, indicating feasible branch formations (Supplementary Figure 10).

To minimize the computational cost, the growing polymer chain was modeled by the first ethylene inserted product with a propyl moiety. The computational results indicate that the coordination of vinyl acetic acid anion via its O atom to Ni2 and its C=C bond to Ni1 center form complex **18** with a relative energy of –89.9 kcal/mol (Supplementary Figure 11). Such a significant exothermic effect could be largely ascribed to the interaction between the ions. This complex surmounts an energy barrier (**19-TS**) of 15.5 kcal/mol to achieve vinyl insertion at the center of Ni1, yielding a six-membered ring chelate product **20**. This step is dramatically exergonic by −20.2 kcal/mol. These results suggest that the bimetal cooperating coordination-insertion event is both kinetically and thermodynamically feasible. Further calculations of subsequent insertion of ethylene into the Ni1-alkyl bond in **20**, yielding intermediate **23**, indicate that ethylene enchainment has an energy barrier of 22.2 kcal/mol and is exergonic by 12.4 kcal/mol. Although such an energy barrier is accessible for ethylene insertion under experimental conditions, it is also possible for **20** to undergo chain-walking. To test this possibility, β-H elimination in **20** was further calculated. It is found that the β-H elimination is more favorable compared with ethylene insertion (barriers of 19.7 *vs.* 22.2 kcal/mol, Supplementary Figure 11). And, the re-insertion of resulting Ni−H bond in **26** to give **27** is also exergonic. Although the re-insertion transition state was not located, this step should be kinetically accessible[34-35] as evidenced by branch formation during polymerization. Therefore, such a chain walking event is likely a favorable process. Thus, we can reasonably assume that such a chain-walking event may continuously occur along with the polymer chain to expand the metallocycle ring and eventually release the coordinating carboxyl

group. Like **8** and the initial active species with Ni−alkyl moiety, the resulting Ni−R species could be involved in the process of monomer enchainment to grow the polymer chain. It is noteworthy that ethylene insertion into Ni−alkyl bond is kinetically more favorable than VA insertion (Supplementary Figures 10 and 11), accounting for more ethylene unit in the polymer chain. Considering the stronger coordination ability and higher concentration of VA in comparison with ethylene in the solution, however, the enchainment of VA can be a competitive process.

Supplementary Tables

Supplementary Table 1. 2a/MMAO catalyzed ethylene polymerization.

Entry[a]	Al/Ni	Yield (g)	Activity[b]	M_w[c]	Đ[c]	Bra.[d]	T_m[e] (°C)
1	2000	0.62	2976	738	2.8	23	107
2	1500	0.61	2928	850	2.4	22	108
3	500	0.54	2592	826	2.5	21	108
4	50	0.34	1632	1074	2.0	21	109
5	25	0.34	1632	1050	2.6	22	108
6	10	0.33	1584	1087	2.1	20	108
7[f,g]	1500	0.16	7680	597	1.9	20	110
8[f]	1500	0.24	5760	904	2.1	18	109
9[f,h]	1500	0.32	2560	1154	2.6	18	108

[a]Conditions: toluene, 100 mL; **2a**, 1.25 μmol; MMAO (2.0 M in heptane); ethylene, 1 atm; 30 °C, 10 min; each entry performed in duplicate. [b]kg/(mol Cat.)·h·atm. [c]M_w (kg/mol) and Đ were determined by GPC. [d]Total branches per 1000 C, determined by quantitative ^{1}H NMR analysis. [e]Determined by DSC. [f]**2a**, 0.25 μmol. [g]5 min. [h]30 min.

Supplementary Table 2. Integration results of two main components of hydrogenated sample from ion flow map

Num.	retention time (min)	Peak integration
methyl isopentanoate	3.67	8153171
methyl pentanoate	4.53	10575494

Methyl isopentanoate/Methyl pentanoate = 0.77 (~3/4)

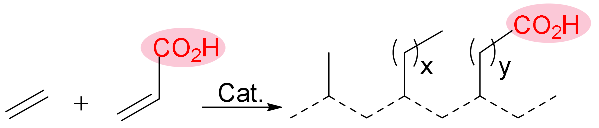

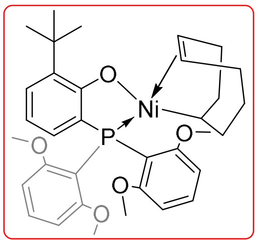

4

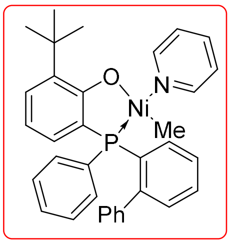

5

Supplementary Table 3. Ethylene/AA copolymerization using Mitsubishi-type catalyst.

Entry[a]	Cat. (μmol)	T (°C)	Comon. (mmol)	P (atm)	t (min)	Yield (mg)	Activity[b]	M_w[c]	Đ[c]	Incorp.[d] (mol%)	T_m (°C)
1	**4**	70	5	20	60	none	-	-	-	-	-
2	**4**	30	75	3	30	none	-	-	-	-	-
3[e]	**4**	30	75	3	30	trace	-	-	-	-	-
4	**5**	50	10	10	60	trace	-	-	-	-	-
5	**5**	30	75	3	30	none	-	-	-	-	-
6[e]	**5**	30	75	3	30	trace	-	-	-	-	-

[a]Conditions: toluene (50 mL), oil bath (30 or 70 °C), cat. (10 μmol), acrylic acid (AA) as comonomer. [b]kg/(mol Cat.)·h. [c]M_w (10^4 g/mol), determined by high temperature GPC. [d]determined by ^1H NMR. [e]AA was premixed with 75 mmol Et_2AlCl before polymerization.

Supplementary Table 4. Control experiment results using saturated acids.

Entry[a]	Cat. (μmol)	Acid monomer/ additive	Pretreatment procedure	Act.[b]	M_w[c]	Đ[c]	Incorp.[d] (mol%)	B[e]	T_c[f] (°C)
1[g]	**2a** (10.0)	VA	A	54	33	3.3	2.1	76	79
2	**2a** (10.0)	acetic acid	A	none	-	-	-	-	-
3	**2a** (10.0)	BA (butyric acid)	A	none	-	-	-	-	-

[a]Conditions: MMAO, 90 mmol (Al/Ni = 2250); acid monomer or additive concentration, 0.3 M, 15 mmol; toluene, 50 mL; ethylene, 1.0 atm; 30 °C, 10 min; each entry performed in duplicate. [b]kg/(mol Cat)·h·atm. [c]M_w (kg/mol) and Đ were determined by GPC. [d]Determined by ^1H NMR. [e]Total branches/1000 C, calculated from ^1H NMR intensity ratio of methyl groups *vs.* overall integral. Note that the branches generated due to polar monomer enchainment are not counted. [f]Determined by DSC. [g]Sample from Table 2, entry 1.

Supplementary Table 5. 29/DEAC catalyzed ethylene/VA cpolymerization.

Entry	VA (mmol)	Yield (g)	Act.[b]	M_w[c] (kg/mol)	Đ[c]	Incorp.[d] (mol%)	B.[e]	T_m[f] (°C)
1	75	2.07	414	40.4	2.6	3.4	62	49(T_c)
2	90	1.94	388	32.8	2.4	3.9	64	n.d.

[a]Conditions: toluene, 50 mL; 3 atm ethylene pressure, Cat. **29** (10 μmol dissolved in 5 mL toluene), DEAC (3.0 M in toluene), 75 mmol. 30 min. [b]kg/(mol Cat.)·h. [c]By GPC. [d]By ^1H NMR. [e]By ^1H NMR. [f]By DSC.

Supplementary Table 6. Crystal data and structure refinement.

Componds No.	(**2a**·$(CH_3CN)_2)_2$	(**2c**·$(THF)_2)_2$	(**7**·THF$)_2$	**29**·2THF
Empirical formula	$C_{70}H_{98}Br_4N_{10}Ni_4O_4$	$C_{66}H_{88}Br_4Cl_8N_4Ni_4O_{12}$	$C_{42}H_{68}Br_2N_2Ni_2O_4$	$C_{38}H_{59}Br_3N_2Ni_2O_3$
Formula weight	1698.06	1967.48	942.22	949.02
Crystal size (mm)	0.30×0.28×0.22	0.20×0.18×0.05	0.20×0.10×0.05	0.12×0.05×0.03
Crystal system	Triclinic	Orthorhombic,	Triclinic	Monoclinic
Space group	P^{-1}	Pbca	P^{-1}	$P2_1/n$
a, Å	14.301(3)	18.225(2)	9.503(12)	9.5447(2)
b, Å	14.617(3)	18.238(2)	9.527(12)	16.8894(3)
c, Å	19.396(4)	22.848(3)	13.702(2)	25.9353(5)
α, deg	85.775(3)	90	69.696(2)	90
β, deg	85.862(4)	90	86.390(3)	95.6410(10)
γ, deg	74.027(3)	90	68.551(2)	90
V, Å^3	3881.6(13)	7594.6(17)	1080.0(3)	4160.63(14)
Z	2	4	1	4
D_{catcd}, Mg/m^3	1.453	1.721	1.449	1.515
Radiation (λ), (Å)	Mo Kα(0.71073)	Mo Kα(0.71073)	Mo Kα(0.71073)	Ga Kα(1.34139)
ϑ range for data collection (deg)	1.75 to 26.00	1.78 to 27.55	1.59 to 30.67	2.979 to 60.681
Absorption coefficient (mm^{-1})	3.064	3.423	2.762	7.582
F(000)	1744	3984	492	1944
Reflections collected / unique	26245/14941 [R(int) = 0.0587]	55929/8730 [R(int) = 0.1725]	10862/6570 [R(int) = 0.0292]	46064/9505 [R(int) = 0.0431]
Goodness-of-fit on F^2	1.102	0.926	1.047	1.039
R1 [I>2σ(I)] ^{a}	0.0696	0.0437	0.0338	0.0407
WR2 [I>2σ(I)] ^{a}	0.2010	0.1017	0.0808	0.1054

^{a} R1 =Σ||F$_o$| - |F$_c$|| / Σ |F$_o$|; wR2 = [Σ [w(F$_o^2$ - F$_c^2$)^2]/P[w(F$_o^2$)^2]]$^{1/2}$.

X-ray structure of **2a**

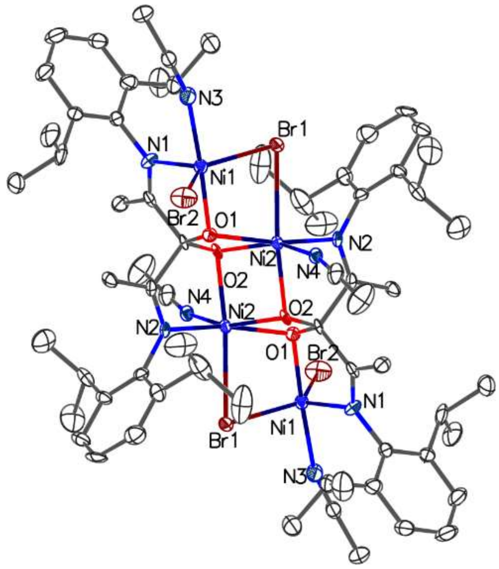

Supplementary Table 7. Bond lengths [Å] for **2a**

Ni(1)-O(1)	1.991(5)	C(8)-C(9)	1.365(14)
Ni(1)-N(3)	2.034(7)	C(9)-C(10)	1.394(15)
Ni(1)-N(1)	2.050(6)	C(10)-C(11)	1.396(12)
Ni(1)-Br(2)	2.4493(13)	C(11)-C(20)	1.523(13)
Ni(1)-Br(1)	2.4933(13)	C(12)-C(17)	1.395(12)
Ni(2)-O(1)	2.041(5)	C(12)-C(13)	1.399(11)
Ni(2)-N(2)#1	2.051(6)	C(13)-C(14)	1.397(11)
Ni(2)-O(2)#1	2.054(5)	C(13)-C(19)	1.529(12)
Ni(2)-N(4)	2.075(6)	C(11)-C(20)	1.523(13)
Ni(2)-O(2)	2.107(5)	C(14)-C(15)	1.374(13)
Ni(2)-Br(1)	2.6546(13)	C(15)-C(16)	1.383(14)
Ni(3)-O(3)	1.983(5)	C(16)-C(17)	1.388(12)
Ni(3)-N(7)	2.045(7)	C(17)-C(18)	1.548(13)
Ni(3)-N(5)	2.049(6)	C(18)-C(27)	1.526(17)
Ni(3)-Br(4)	2.4419(14)	C(18)-C(26)	1.528(17)
Ni(3)-Br(3)	2.4844(13)	C(19)-C(29)	1.501(13)
Ni(4)-O(3)	2.027(5)	C(19)-C(28)	1.528(12)
Ni(4)-O(4)#2	2.046(5)	C(20)-C(25)	1.529(13)
Ni(4)-N(6)#2	2.047(6)	C(20)-C(24)	1.543(14)
Ni(4)-N(8)	2.054(6)	C(21)-C(22)	1.536(12)
Ni(4)-O(4)	2.121(5)	C(21)-C(23)	1.544(12)
Ni(4)-Br(3)	2.6989(12)	C(30)-C(31)	1.443(12)
O(1)-C(3)	1.397(8)	C(32)-C(33)	1.455(13)

O(2)-C(3)	1.380(9)	C(34)-C(35)	1.500(10)
O(2)-Ni(2)#1	2.054(5)	C(35)-C(36)	1.529(9)
O(3)-C(36)	1.393(9)	C(36)-C(37)	1.546(9)
O(4)-C(36)	1.394(8)	C(37)-C(38)	1.486(10)
O(4)-Ni(4)#2	2.046(5)	C(39)-C(40)	1.384(11)
N(1)-C(2)	1.297(10)	C(39)-C(44)	1.428(11)
N(1)-C(6)	1.450(10)	C(40)-C(41)	1.405(11)
N(2)-C(4)	1.277(10)	C(40)-C(53)	1.527(12)
N(2)-C(12)	1.463(10)	C(41)-C(42)	1.369(14)
N(2)-Ni(2)#1	2.051(6)	C(42)-C(43)	1.383(13)
N(3)-C(31)	1.149(11)	C(43)-C(44)	1.410(11)
N(4)-C(33)	1.152(11)	C(44)-C(54)	1.492(12)
N(5)-C(35)	1.287(10)	C(45)-C(46)	1.394(11)
N(5)-C(39)	1.447(9)	C(45)-C(50)	1.415(11)
N(6)-C(37)	1.285(9)	C(46)-C(47)	1.408(11)
N(6)-C(45)	1.449(9)	C(46)-C(52)	1.533(12)
N(6)-Ni(4)#2	2.047(6)	C(47)-C(48)	1.355(13)
N(7)-C(64)	1.130(11)	C(48)-C(49)	1.405(14)
N(8)-C(66)	1.145(10)	C(49)-C(50)	1.398(11)
N(9)-C(68)	1.105(17)	C(50)-C(51)	1.522(12)
N(10)-C(70)	1.110(15)	C(51)-C(61)	1.475(12)
C(1)-C(2)	1.493(10)	C(51)-C(62)	1.514(13)
C(2)-C(3)	1.523(10)	C(52)-C(60)	1.507(12)
C(3)-C(4)	1.553(10)	C(52)-C(59)	1.542(11)
C(6)-C(11)	1.396(12)	C(53)-C(57)	1.522(12)
C(6)-C(7)	1.412(11)	C(53)-C(58)	1.525(12)
C(7)-C(8)	1.402(11)	C(54)-C(56)	1.533(12)
C(4)-C(5)	1.493(10)	C(54)-C(55)	1.552(12)
C(5)-H(5A)	0.9800	C(63)-C(64)	1.461(12)
C(5)-H(5B)	0.9800	C(65)-C(66)	1.461(12)
C(5)-H(5C)	0.9800	C(67)-C(68)	1.421(18)
C(7)-C(21)	1.514(12)	C(69)-C(70)	1.473(17)

Supplementary Table 8. Bond angles [o] for **2a**

O(1)-Ni(1)-N(3)	174.9(2)	C(5)-C(4)-C(3)	116.9(6)
O(1)-Ni(1)-N(1)	82.6(2)	N(1)-C(2)-C(1)	125.6(7)
N(3)-Ni(1)-N(1)	92.3(3)	C(11)-C(6)-C(7)	121.7(7)
O(1)-Ni(1)-Br(2)	93.28(15)	C(11)-C(6)-N(1)	120.1(7)
N(3)-Ni(1)-Br(2)	90.2(2)	C(7)-C(6)-N(1)	117.9(7)
N(1)-Ni(1)-Br(2)	125.37(19)	C(8)-C(7)-C(6)	117.6(8)
O(1)-Ni(1)-Br(1)	85.68(15)	C(8)-C(7)-C(21)	120.7(8)

N(3)-Ni(1)-Br(1)	94.5(2)	C(6)-C(7)-C(21)	121.5(7)
N(1)-Ni(1)-Br(1)	98.54(19)	C(9)-C(8)-C(7)	121.8(9)
Br(2)-Ni(1)-Br(1)	135.63(5)	C(8)-C(9)-C(10)	119.5(8)
O(1)-Ni(2)-N(2)#1	165.8(2)	C(9)-C(10)-C(11)	121.6(9)
O(1)-Ni(2)-O(2)#1	106.0(2)	C(10)-C(11)-C(6)	117.8(9)
N(2)#1-Ni(2)-O(2)#1	81.5(2)	C(10)-C(11)-C(20)	118.8(8)
O(1)-Ni(2)-N(4)	94.9(2)	C(6)-C(11)-C(20)	123.4(7)
N(2)#1-Ni(2)-N(4)	97.6(2)	C(17)-C(12)-C(13)	122.3(7)
O(2)#1-Ni(2)-N(4)	86.1(2)	C(17)-C(12)-N(2)	119.0(7)
O(1)-Ni(2)-O(2)	66.62(19)	C(13)-C(12)-N(2)	118.7(7)
N(2)#1-Ni(2)-O(2)	103.3(2)	C(14)-C(13)-C(12)	117.8(8)
O(2)#1-Ni(2)-O(2)	82.2(2)	C(14)-C(13)-C(19)	120.0(7)
N(4)-Ni(2)-O(2)	154.2(2)	C(12)-C(13)-C(19)	122.1(7)
O(1)-Ni(2)-Br(1)	80.56(14)	C(15)-C(14)-C(13)	120.4(8)
N(2)#1-Ni(2)-Br(1)	92.08(17)	C(14)-C(15)-C(16)	120.8(8)
O(2)#1-Ni(2)-Br(1)	173.44(14)	C(15)-C(16)-C(17)	120.7(9)
N(4)-Ni(2)-Br(1)	93.30(17)	C(16)-C(17)-C(12)	117.8(8)
O(2)-Ni(2)-Br(1)	100.82(14)	C(16)-C(17)-C(18)	120.3(8)
O(3)-Ni(3)-N(7)	174.2(3)	C(12)-C(17)-C(18)	121.8(7)
O(3)-Ni(3)-N(5)	82.3(2)	C(27)-C(18)-C(26)	112.1(9)
N(7)-Ni(3)-N(5)	91.9(3)	C(27)-C(18)-C(17)	109.1(10)
O(3)-Ni(3)-Br(4)	91.55(15)	C(26)-C(18)-C(17)	110.9(9)
N(7)-Ni(3)-Br(4)	91.7(2)	C(29)-C(19)-C(28)	111.9(8)
N(5)-Ni(3)-Br(4)	126.84(19)	C(29)-C(19)-C(13)	111.4(7)
O(3)-Ni(3)-Br(3)	86.58(14)	C(28)-C(19)-C(13)	113.1(8)
N(7)-Ni(3)-Br(3)	94.8(2)	C(11)-C(20)-C(25)	109.3(9)
N(5)-Ni(3)-Br(3)	99.47(18)	C(11)-C(20)-C(24)	111.5(7)
Br(4)-Ni(3)-Br(3)	132.98(5)	C(25)-C(20)-C(24)	110.8(9)
O(3)-Ni(4)-O(4)#2	106.01(19)	C(7)-C(21)-C(22)	113.4(7)
O(3)-Ni(4)-N(6)#2	165.0(2)	C(7)-C(21)-C(23)	108.5(7)
O(4)#2-Ni(4)-N(6)#2	81.9(2)	C(22)-C(21)-C(23)	111.2(7)
O(3)-Ni(4)-N(8)	91.4(2)	N(3)-C(31)-C(30)	177.4(9)
O(4)#2-Ni(4)-N(8)	88.1(2)	N(4)-C(33)-C(32)	177.1(10)
N(6)#2-Ni(4)-N(8)	101.8(2)	N(5)-C(35)-C(34)	126.0(6)
O(3)-Ni(4)-O(4)	66.86(19)	N(5)-C(35)-C(36)	117.1(6)
O(4)#2-Ni(4)-O(4)	82.5(2)	C(34)-C(35)-C(36)	116.9(6)
N(6)#2-Ni(4)-O(4)	102.3(2)	O(3)-C(36)-O(4)	110.2(5)
N(8)-Ni(4)-O(4)	152.5(2)	O(3)-C(36)-C(35)	112.6(6)
O(3)-Ni(4)-Br(3)	80.14(14)	O(4)-C(36)-C(35)	109.7(6)
O(4)#2-Ni(4)-Br(3)	173.27(14)	O(3)-C(36)-C(37)	101.9(5)
N(6)#2-Ni(4)-Br(3)	92.78(17)	O(4)-C(36)-C(37)	112.6(6)
N(8)-Ni(4)-Br(3)	89.05(16)	C(35)-C(36)-C(37)	109.6(6)
O(4)-Ni(4)-Br(3)	102.71(13)	N(6)-C(37)-C(38)	125.8(6)
Ni(1)-Br(1)-Ni(2)	80.59(4)	N(6)-C(37)-C(36)	116.7(6)
Ni(3)-Br(3)-Ni(4)	79.75(3)	C(38)-C(37)-C(36)	116.9(6)
C(3)-O(1)-Ni(1)	113.4(4)	C(40)-C(39)-C(44)	121.4(7)

C(3)-O(1)-Ni(2)	92.7(4)	C(40)-C(39)-N(5)	118.5(7)
Ni(1)-O(1)-Ni(2)	111.4(2)	C(44)-C(39)-N(5)	119.8(7)
C(3)-O(2)-Ni(2)#1	112.0(4)	C(39)-C(40)-C(41)	119.1(8)
C(3)-O(2)-Ni(2)	90.4(4)	C(39)-C(40)-C(53)	122.5(7)
Ni(2)#1-O(2)-Ni(2)	97.8(2)	C(41)-C(40)-C(53)	118.3(7)
C(36)-O(3)-Ni(3)	114.0(4)	C(42)-C(41)-C(40)	121.0(8
C(36)-O(3)-Ni(4)	93.4(4)	C(41)-C(42)-C(43)	119.8(8)
Ni(3)-O(3)-Ni(4)	112.1(2)	C(42)-C(43)-C(44)	122.0(8)
C(36)-O(4)-Ni(4)#2	111.9(4)	C(43)-C(44)-C(39)	116.6(8)
C(36)-O(4)-Ni(4)	89.5(4)	C(43)-C(44)-C(54)	119.8(8)
Ni(4)#2-O(4)-Ni(4)	97.5(2)	C(39)-C(44)-C(54)	123.6(7)
C(2)-N(1)-C(6)	122.2(7)	C(46)-C(45)-C(50)	122.8(7)
C(2)-N(1)-Ni(1)	112.8(5)	C(46)-C(45)-N(6)	119.7(7)
C(6)-N(1)-Ni(1)	125.0(5)	C(50)-C(45)-N(6)	117.5(7)
C(4)-N(2)-C(12)	116.7(6)	C(45)-C(46)-C(47)	117.6(8)
C(4)-N(2)-Ni(2)#1	114.9(5)	C(45)-C(46)-C(52)	120.9(7)
C(12)-N(2)-Ni(2)#1	127.6(5)	C(47)-C(46)-C(52)	121.5(7)
C(31)-N(3)-Ni(1)	163.0(7)	C(48)-C(47)-C(46)	121.3(8)
C(33)-N(4)-Ni(2)	164.5(6)	C(47)-C(48)-C(49)	120.6(7)
C(35)-N(5)-C(39)	121.7(6)	C(50)-C(49)-C(48)	120.9(8)
C(35)-N(5)-Ni(3)	113.5(5)	C(49)-C(50)-C(45)	116.8(8)
C(39)-N(5)-Ni(3)	124.8(5)	C(49)-C(50)-C(51)	119.5(7)
C(37)-N(6)-C(45)	117.7(6)	C(45)-C(50)-C(51)	123.7(6)
C(37)-N(6)-Ni(4)#2	114.5(5)	C(61)-C(51)-C(62)	110.6(8)
C(45)-N(6)-Ni(4)#2	127.2(5)	C(61)-C(51)-C(50)	114.9(7)
C(64)-N(7)-Ni(3)	163.6(7)	C(62)-C(51)-C(50)	110.0(7)
C(66)-N(8)-Ni(4)	160.3(6)	C(60)-C(52)-C(46)	112.7(7)
N(1)-C(2)-C(1)	125.6(7)	C(60)-C(52)-C(59)	110.8(7)
N(1)-C(2)-C(3)	117.5(6)	C(46)-C(52)-C(59)	110.7(7)
C(1)-C(2)-C(3)	116.8(6)	C(57)-C(53)-C(58)	111.6(7)
O(2)-C(3)-O(1)	110.3(5)	C(57)-C(53)-C(40)	108.8(7)
O(2)-C(3)-C(2)	110.4(6)	C(58)-C(53)-C(40)	113.7(7)
O(1)-C(3)-C(2)	112.8(6)	C(44)-C(54)-C(56)	110.5(8)
O(2)-C(3)-C(4)	113.1(6)	C(44)-C(54)-C(55)	111.8(7)
O(1)-C(3)-C(4)	101.4(6)	C(56)-C(54)-C(55)	109.6(8)
C(2)-C(3)-C(4)	108.6(5)	N(9)-C(68)-C(67)	178.6(15
N(2)-C(4)-C(5)	126.5(7)	N(10)-C(70)-C(69)	177.8(15)
N(2)-C(4)-C(3)	116.4(6)		

X-ray structure of **2c**

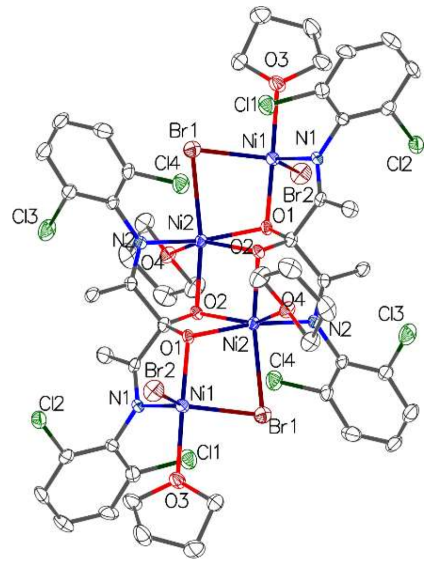

Supplementary Table 9. Bond lengths [Å] for **2c**

Ni(1)-O(1)	2.002(3)	O(6)-C(33)	1.415(7)
Ni(1)-N(1)	2.057(4)	O(6)-C(30)	1.420(7)
Ni(1)-O(3)	2.124(3)	C(1)-C(2)	1.501(6)
Ni(1)-Br(2)	2.4189(8)	C(2)-C(3)	1.516(6)
Ni(1)-Br(1)	2.4590(8)	C(3)-C(4)	1.535(6)
Ni(2)-O(2)	2.047(3)	C(4)-C(5)	1.487(6)
Ni(2)-O(1)#1	2.056(3)	C(6)-C(11)	1.389(6)
Ni(2)-N(2)	2.078(4)	C(6)-C(7)	1.390(6)
Ni(2)-O(2)#1	2.119(3)	C(7)-C(8)	1.386(7)
Ni(2)-O(4)	2.125(3)	C(8)-C(9)	1.378(7)
Ni(2)-Br(1)#1	2.6089(8)	C(9)-C(10)	1.380(7)
Br(1)-Ni(2)#1	2.6090(8)	C(10)-C(11)	1.382(7)
Cl(1)-C(7)	1.731(5)	C(12)-C(17)	1.385(7)
Cl(2)-C(11)	1.745(5)	C(12)-C(13)	1.399(6)
Cl(3)-C(13)	1.730(5)	C(13)-C(14)	1.381(7)
Cl(4)-C(17)	1.730(5)	C(14)-C(15)	1.381(7)
N(1)-C(2)	1.279(6)	C(15)-C(16)	1.390(7)
N(1)-C(6)	1.438(6)	C(16)-C(17)	1.389(7)
N(2)-C(4)	1.273(6)	C(18)-C(19)	1.516(7)
N(2)-C(12)	1.432(5)	C(19)-C(20)	1.528(7)
O(1)-C(3)	1.402(5)	C(20)-C(21)	1.519(7)
O(1)-Ni(2)#1	2.056(3)	C(22)-C(23)	1.510(7)
O(2)-C(3)	1.391(5)	C(23)-C(24)	1.524(8)
O(2)-Ni(2)#1	2.119(3)	C(24)-C(25)	1.503(7)

O(3)-C(18)	1.450(5)	C(26)-C(27)	1.359(9)
O(3)-C(21)	1.454(6)	C(27)-C(28)	1.385(9)
O(4)-C(25)	1.448(6)	C(28)-C(29)	1.519(9)
O(4)-C(22)	1.450(5)	C(30)-C(31)	1.508(8)
O(5)-C(26)	1.494(11)	C(31)-C(32)	1.519(8)
O(5)-C(29)	1.510(11)	C(32)-C(33)	1.515(7)

Supplementary Table 10. Bond angles [o] for **2c**

O(1)-Ni(1)-N(1)	82.34(13)	O(2)-C(3)-O(1)	110.1(3)
O(1)-Ni(1)-O(3)	174.99(12)	O(2)-C(3)-C(2)	106.1(3)
N(1)-Ni(1)-O(3)	94.52(13)	O(1)-C(3)-C(2)	113.1(4)
O(1)-Ni(1)-Br(2)	94.21(9)	O(2)-C(3)-C(4)	113.8(4)
N(1)-Ni(1)-Br(2)	122.47(10)	O(1)-C(3)-C(4)	105.0(3)
O(3)-Ni(1)-Br(2)	90.77(9)	C(2)-C(3)-C(4)	109.0(3)
O(1)-Ni(1)-Br(1)	86.48(8)	N(2)-C(4)-C(5)	126.4(4)
N(1)-Ni(1)-Br(1)	104.67(10)	N(2)-C(4)-C(3)	116.7(4)
O(3)-Ni(1)-Br(1)	90.56(9)	C(5)-C(4)-C(3)	116.8(4)
Br(2)-Ni(1)-Br(1)	132.57(3)	C(11)-C(6)-C(7)	117.7(4)
O(2)-Ni(2)-O(1)#1	106.80(12)	C(11)-C(6)-N(1)	121.4(4)
O(2)-Ni(2)-N(2)	80.99(13)	C(7)-C(6)-N(1)	120.9(4)
O(1)#1-Ni(2)-N(2)	165.92(13)	C(8)-C(7)-C(6)	121.0(4)
O(2)-Ni(2)-O(2)#1	79.41(12)	C(8)-C(7)-Cl(1)	119.5(4)
O(1)#1-Ni(2)-O(2)#1	66.46(11)	C(6)-C(7)-Cl(1)	119.4(4)
N(2)-Ni(2)-O(2)#1	104.48(12)	C(9)-C(8)-C(7)	119.7(5)
O(2)-Ni(2)-O(4)	85.19(12)	C(8)-C(9)-C(10)	120.6(5)
O(1)#1-Ni(2)-O(4)	94.16(12)	C(9)-C(10)-C(11)	119.0(5)
N(2)-Ni(2)-O(4)	98.24(13)	C(10)-C(11)-C(6)	122.0(5)
O(2)#1-Ni(2)-O(4)	150.02(12)	C(10)-C(11)-Cl(2)	119.4(4)
O(2)-Ni(2)-Br(1)#1	171.09(8)	C(6)-C(11)-Cl(2)	118.6(4)
O(1)#1-Ni(2)-Br(1)#1	81.51(9)	C(17)-C(12)-C(13)	117.3(4)
N(2)-Ni(2)-Br(1)#1	91.56(10)	C(17)-C(12)-N(2)	121.3(4)
O(2)#1-Ni(2)-Br(1)#1	107.36(8)	C(13)-C(12)-N(2)	121.4(4)
O(4)-Ni(2)-Br(1)#1	91.10(9)	C(14)-C(13)-C(12)	121.3(5)
Ni(1)-Br(1)-Ni(2)#1	81.94(2)	C(14)-C(13)-Cl(3)	119.2(4)
C(2)-N(1)-C(6)	118.6(4)	C(12)-C(13)-Cl(3)	119.6(4)
C(2)-N(1)-Ni(1)	113.3(3)	C(15)-C(14)-C(13)	120.2(5)
C(6)-N(1)-Ni(1)	128.1(3)	C(14)-C(15)-C(16)	120.0(5)
C(4)-N(2)-C(12)	119.6(4)	C(17)-C(16)-C(15)	118.9(5)
C(4)-N(2)-Ni(2)	114.6(3)	C(12)-C(17)-C(16)	122.3(4)
C(12)-N(2)-Ni(2)	125.6(3)	C(12)-C(17)-Cl(4)	119.2(4)
C(3)-O(1)-Ni(1)	112.9(3)	C(16)-C(17)-Cl(4)	118.5(4)
C(3)-O(1)-Ni(2)#1	92.9(2)	O(3)-C(18)-C(19)	105.1(4)
Ni(1)-O(1)-Ni(2)#1	110.04(13)	C(18)-C(19)-C(20)	102.3(4)
C(3)-O(2)-Ni(2)	112.1(3)	C(21)-C(20)-C(19)	101.1(4)
C(3)-O(2)-Ni(2)#1	90.6(2)	O(3)-C(21)-C(20)	107.1(4)

Ni(2)-O(2)-Ni(2)#1	100.58(12)	O(4)-C(22)-C(23)	102.8(4)
C(18)-O(3)-C(21)	108.6(4)	C(22)-C(23)-C(24)	105.2(4)
C(18)-O(3)-Ni(1)	117.4(3)	C(25)-C(24)-C(23)	104.3(4)
C(21)-O(3)-Ni(1)	120.6(3)	O(4)-C(25)-C(24)	105.9(4)
C(25)-O(4)-C(22)	105.0(4)	C(27)-C(26)-O(5)	112.2(7)
C(25)-O(4)-Ni(2)	112.8(3)	C(26)-C(27)-C(28)	110.1(6)
C(22)-O(4)-Ni(2)	130.6(3)	C(27)-C(28)-C(29)	106.9(6)
C(26)-O(5)-C(29)	102.1(8)	O(5)-C(29)-C(28)	105.6(7)
C(33)-O(6)-C(30)	108.6(5)	O(6)-C(30)-C(31)	106.2(5)
N(1)-C(2)-C(1)	124.7(4)	C(30)-C(31)-C(32)	100.9(5)
N(1)-C(2)-C(3)	117.9(4)	C(33)-C(32)-C(31)	102.2(5)
C(1)-C(2)-C(3)	117.3(4)	O(6)-C(33)-C(32)	107.8(5)

X-ray structure of **7**

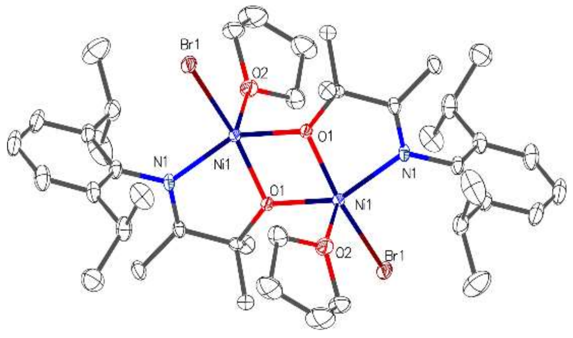

Supplementary Table 11. Bond lengths [Å] for **7**

Ni(1)-O(1)	1.9668(19)	C(3)-C(4)	1.386(5)
Ni(1)-O(1)#1	2.0002(18)	C(4)-C(5)	1.381(5)
Ni(1)-O(2)	2.089(2)	C(5)-C(6)	1.399(4)
Ni(1)-N(1)	2.101(2)	C(6)-C(7)	1.532(4)
Ni(1)-Br(1)	2.4554(5)	C(7)-C(9)	1.516(5)
N(1)-C(14)	1.289(3)	C(7)-C(8)	1.526(5)
N(1)-C(1)	1.445(3)	C(10)-C(12)	1.509(5)
O(1)-C(15)	1.412(3)	C(10)-C(11)	1.543(5)
O(1)-Ni(1)#1	2.0001(18)	C(13)-C(14)	1.493(4)
O(2)-C(18)	1.450(4)	C(14)-C(15)	1.528(4)
O(2)-C(21)	1.463(4)	C(15)-C(16)	1.534(4)
C(1)-C(2)	1.400(4)	C(15)-C(17)	1.539(4)
C(1)-C(6)	1.406(4)	C(18)-C(19)	1.522(4)
C(2)-C(3)	1.395(4)	C(19)-C(20)	1.512(6)
C(2)-C(10)	1.530(4)	C(20)-C(21)	1.480(5)

Supplementary Table 12. Bond angles [o] for **7**

O(1)-Ni(1)-O(1)#1	77.20(8)	C(4)-C(3)-C(2)	121.1(3)
O(1)-Ni(1)-O(2)	95.90(8)	C(5)-C(4)-C(3)	120.1(3)
O(1)#1-Ni(1)-O(2)	97.27(8)	C(4)-C(5)-C(6)	121.0(3)
O(1)-Ni(1)-N(1)	79.12(8)	C(5)-C(6)-C(1)	117.8(3)
O(1)#1-Ni(1)-N(1)	149.72(8)	C(5)-C(6)-C(7)	120.5(3)
O(2)-Ni(1)-N(1)	103.70(9)	C(1)-C(6)-C(7)	121.7(2)
O(1)-Ni(1)-Br(1)	165.35(6)	C(9)-C(7)-C(8)	111.1(3)
O(1)#1-Ni(1)-Br(1)	101.92(6)	C(9)-C(7)-C(6)	112.1(3)
O(2)-Ni(1)-Br(1)	98.71(6)	C(8)-C(7)-C(6)	110.8(3)
N(1)-Ni(1)-Br(1)	96.28(6)	C(12)-C(10)-C(2)	111.3(3)
C(14)-N(1)-C(1)	118.2(2)	C(12)-C(10)-C(11)	109.8(3)
C(14)-N(1)-Ni(1)	114.41(17)	C(2)-C(10)-C(11)	112.2(3)
C(1)-N(1)-Ni(1)	127.27(17)	N(1)-C(14)-C(13)	124.1(2)
C(15)-O(1)-Ni(1)	118.92(15)	N(1)-C(14)-C(15)	117.8(2)
C(15)-O(1)-Ni(1)#1	137.31(15)	C(13)-C(14)-C(15)	118.0(2)
Ni(1)-O(1)-Ni(1)#1	102.81(8)	O(1)-C(15)-C(14)	108.7(2)
C(18)-O(2)-C(21)	108.7(2)	O(1)-C(15)-C(16)	111.1(2)
C(18)-O(2)-Ni(1)	119.65(17)	C(14)-C(15)-C(16)	108.7(2)
C(21)-O(2)-Ni(1)	124.7(2)	O(1)-C(15)-C(17)	110.5(2)
C(2)-C(1)-C(6)	122.0(3)	C(14)-C(15)-C(17)	108.2(2)
C(2)-C(1)-N(1)	119.3(2)	C(16)-C(15)-C(17)	109.7(2)
C(6)-C(1)-N(1)	118.7(2)	O(2)-C(18)-C(19)	106.5(3)
C(3)-C(2)-C(1)	117.9(3)	C(20)-C(19)-C(18)	103.2(3)
C(3)-C(2)-C(10)	119.7(3)	C(21)-C(20)-C(19)	104.4(3)
C(1)-C(2)-C(10)	122.3(2)	O(2)-C(21)-C(20)	103.5(3)

X-ray structure of **29**

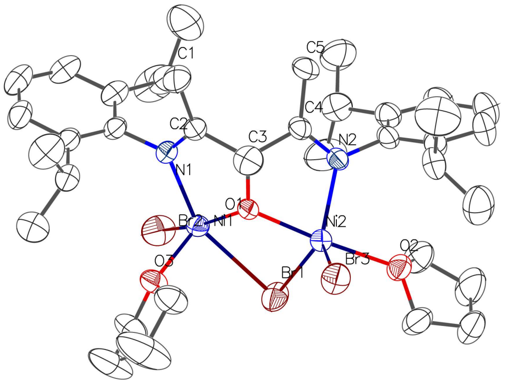

Supplementary Table 13. Bond lengths [Å] for **29**

Br(1)-Ni(1)	2.6521(6)	C(8)-C(16)	1.518(5)
Br(1)-Ni(2)	2.4918(6)	C(9)-C(10)	1.382(6)
Br(2)-Ni(1)	2.3941(6)	C(10)-C(11)	1.369(6)
Br(3)-Ni(2)	2.4194(6)	C(11)-C(12)	1.399(5)
Ni(1)-O(1)	1.958(2)	C(12)-C(13)	1.507(6)
Ni(1)-O(3)	2.034(2)	C(13)-C(14)	1.529(7)
Ni(1)-N(1)	2.090(3)	C(13)-C(15)	1.544(7)
Ni(2)-O(1)	1.976(2)	C(16)-C(17)	1.524(6)
Ni(2)-O(2)	2.137(2)	C(16)-C(18)	1.521(6)
Ni(2)-N(2)	2.063(3)	C(19)-C(20)	1.401(5)
O(1)-C(3)	1.396(4)	C(19)-C(24)	1.401(5)
O(2)-C(31)	1.445(4)	C(20)-C(21)	1.394(5)
O(2)-C(34)	1.438(4)	C(20)-C(28)	1.510(5)
O(3)-C(35)	1.459(4)	C(21)-C(22)	1.378(6)
O(3)-C(38)	1.450(4)	C(22)-C(23)	1.362(6)
N(1)-C(2)	1.284(4)	C(23)-C(24)	1.400(5)
N(1)-C(7)	1.456(4)	C(24)-C(25)	1.501(5)
N(2)-C(4)	1.281(4)	C(25)-C(26)	1.517(6)
N(2)-C(19)	1.445(4)	C(25)-C(27)	1.533(6)
C(1)-C(2)	1.501(5)	C(28)-C(29)	1.516(6)
C(2)-C(3)	1.535(4)	C(28)-C(30)	1.506(6)
C(3)-C(4)	1.536(4)	C(31)-C(32)	1.471(6)
C(3)-C(6)	1.539(5)	C(32)-C(33)	1.493(7)
C(4)-C(5)	1.495(5)	C(33)-C(34)	1.497(6)
C(7)-C(8)	1.396(5)	C(35)-C(36)	1.451(7)
C(7)-C(12)	1.400(5)	C(36)-C(37)	1.464(8)
C(8)-C(9)	1.393(5)	C(37)-C(38)	1.503(7)

Supplementary Table 14. Bond angles [o] for **29**

Ni(2)-Br(1)-Ni(1)	78.546(17)	N(2)-C(4)-C(5)	123.6(3)
Br(2)-Ni(1)-Br(1)	93.37(2)	C(5)-C(4)-C(3)	121.1(3)
O(1)-Ni(1)-Br(1)	81.42(6)	C(8)-C(7)-N(1)	119.4(3)
O(1)-Ni(1)-Br(2)	161.32(7)	C(8)-C(7)-C(12)	122.8(3)
O(1)-Ni(1)-O(3)	95.92(9)	C(12)-C(7)-N(1)	117.8(3)
O(1)-Ni(1)-N(1)	78.77(9)	C(7)-C(8)-C(16)	123.7(3)
O(3)-Ni(1)-Br(1)	92.45(7)	C(9)-C(8)-C(7)	117.5(3)
O(3)-Ni(1)-Br(2)	102.23(7)	C(9)-C(8)-C(16)	118.8(3)
O(3)-Ni(1)-N(1)	103.51(10)	C(10)-C(9)-C(8)	121.3(4)
N(1)-Ni(1)-Br(1)	155.65(7)	C(11)-C(10)-C(9)	119.8(3)

N(1)-Ni(1)-Br(2)	100.92(8)	C(10)-C(11)-C(12)	122.0(4)
Br(3)-Ni(2)-Br(1)	118.54(2)	C(7)-C(12)-C(13)	123.5(3)
O(1)-Ni(2)-Br(1)	85.36(6)	C(11)-C(12)-C(7)	116.6(3)
O(1)-Ni(2)-Br(3)	98.40(6)	C(11)-C(12)-C(13)	119.8(4)
O(1)-Ni(2)-O(2)	170.18(9)	C(12)-C(13)-C(14)	112.7(4)
O(1)-Ni(2)-N(2)	78.27(9)	C(12)-C(13)-C(15)	110.1(4)
O(2)-Ni(2)-Br(1)	90.86(7)	C(14)-C(13)-C(15)	110.1(4)
O(2)-Ni(2)-Br(3)	91.37(7)	C(8)-C(16)-C(17)	113.1(3)
N(2)-Ni(2)-Br(1)	129.19(7)	C(8)-C(16)-C(18)	110.5(3)
N(2)-Ni(2)-Br(3)	111.33(7)	C(18)-C(16)-C(17)	109.2(4)
N(2)-Ni(2)-O(2)	97.30(10)	C(20)-C(19)-N(2)	118.6(3)
Ni(1)-O(1)-Ni(2)	111.82(10)	C(24)-C(19)-N(2)	118.1(3)
C(3)-O(1)-Ni(1)	119.05(18)	C(24)-C(19)-C(20)	123.1(3)
C(3)-O(1)-Ni(2)	115.39(17)	C(19)-C(20)-C(28)	122.9(3)
C(31)-O(2)-Ni(2)	121.8(2)	C(21)-C(20)-C(19)	117.1(4)
C(34)-O(2)-Ni(2)	120.1(2)	C(21)-C(20)-C(28)	120.0(4)
C(34)-O(2)-C(31)	105.6(3)	C(22)-C(21)-C(20)	121.0(4)
C(35)-O(3)-Ni(1)	120.9(2)	C(23)-C(22)-C(21)	120.5(4)
C(38)-O(3)-Ni(1)	119.7(2)	C(22)-C(23)-C(24)	122.0(4)
C(38)-O(3)-C(35)	107.6(3)	C(19)-C(24)-C(25)	122.7(3)
C(2)-N(1)-Ni(1)	115.0(2)	C(23)-C(24)-C(19)	116.3(4)
C(2)-N(1)-C(7)	118.4(3)	C(23)-C(24)-C(25)	121.0(4)
C(7)-N(1)-Ni(1)	126.4(2)	C(24)-C(25)-C(26)	110.0(4)
C(4)-N(2)-Ni(2)	115.7(2)	C(24)-C(25)-C(27)	114.2(4)
C(4)-N(2)-C(19)	121.7(3)	C(26)-C(25)-C(27)	110.1(4)
C(19)-N(2)-Ni(2)	122.6(2)	C(20)-C(28)-C(29)	112.6(4)
N(1)-C(2)-C(1)	123.4(3)	C(30)-C(28)-C(20)	111.2(4)
N(1)-C(2)-C(3)	116.7(3)	C(30)-C(28)-C(29)	110.9(4)
C(1)-C(2)-C(3)	119.6(3)	O(2)-C(31)-C(32)	105.7(3)
O(1)-C(3)-C(2)	108.5(2)	C(31)-C(32)-C(33)	107.0(4)
O(1)-C(3)-C(4)	108.3(2)	C(32)-C(33)-C(34)	104.4(3)
O(1)-C(3)-C(6)	110.4(3)	O(2)-C(34)-C(33)	105.7(3)
C(2)-C(3)-C(4)	113.6(3)	C(36)-C(35)-O(3)	107.0(4)
C(2)-C(3)-C(6)	108.3(3)	C(35)-C(36)-C(37)	108.0(4)
C(4)-C(3)-C(6)	107.7(3)	C(36)-C(37)-C(38)	105.5(4)
N(2)-C(4)-C(3)	115.1(3)	O(3)-C(38)-C(37)	104.2(4)

Supplementary Figures

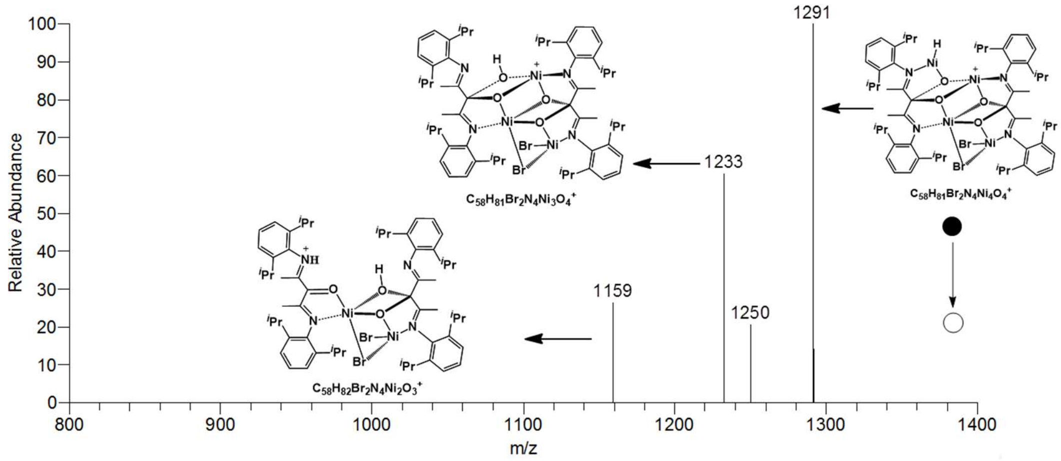

Supplementary Figure 1. MS/MS spectrometry spectrum of signals at *m/z* 1291.

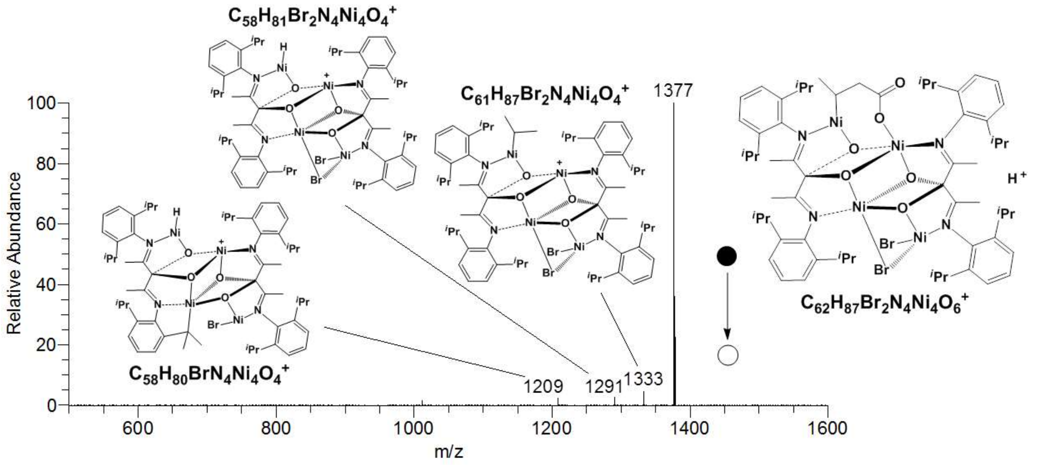

Supplementary Figure 2. MS/MS spectrometry spectrum of signals at *m/z* 1377.

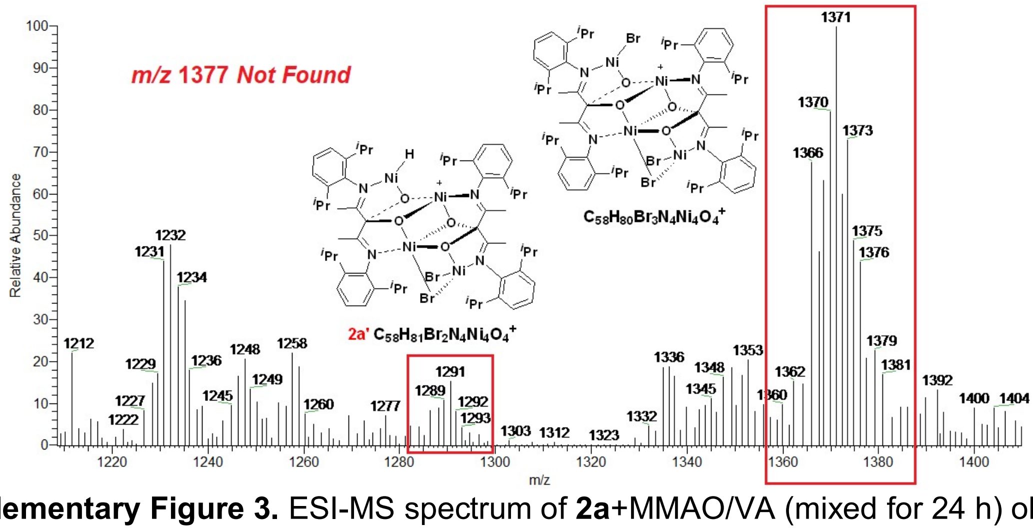

Supplementary Figure 3. ESI-MS spectrum of **2a**+MMAO/VA (mixed for 24 h) obtained by using SAESI-MS method (no signals were detected at *m/z* 1377).

a

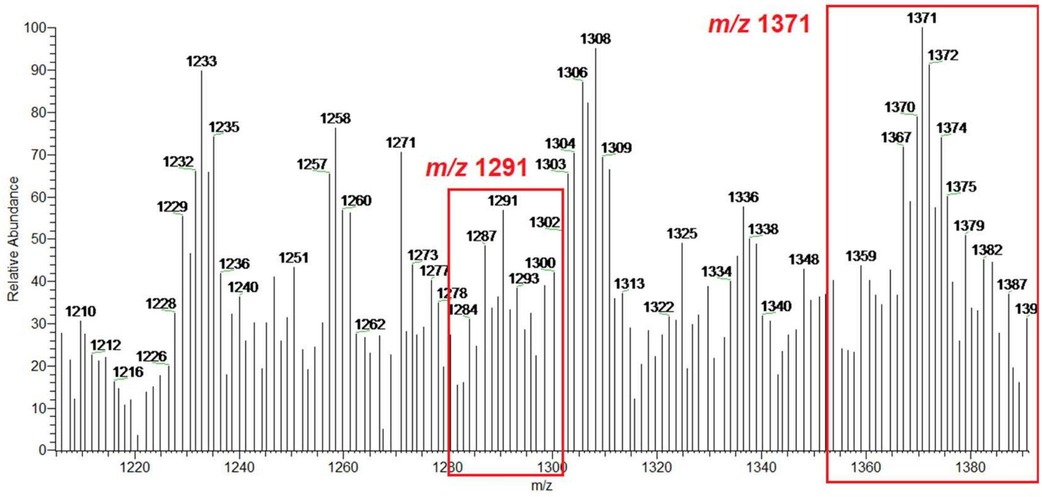

b

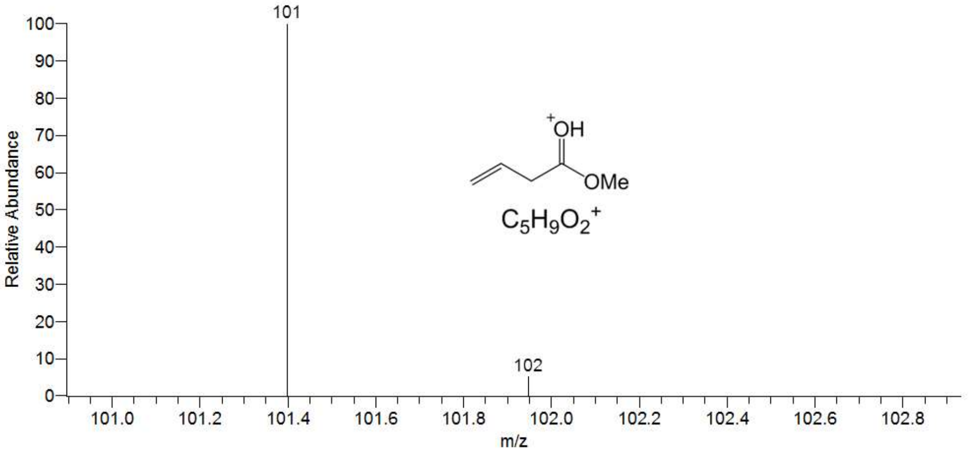

Supplementary Figure 4. ESI-MS spectrum of **2a**/MMAO/MVA obtained by using SAESI-MS method. **a**, signal at *m/z* 1291 corresponding to [**2a**-2Br+H]$^+$ **b**, signal of MVA at *m/z* 101.

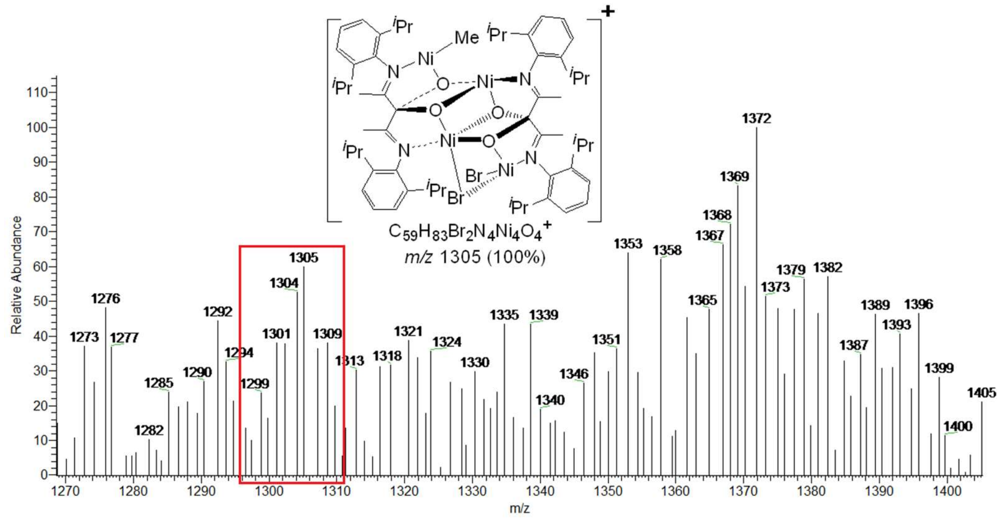

Supplementary Figure 5. SAESI-MS spectra of **2a**/MAO showing signals at *m/z* 1305 corresponding to [**2a**-2Br+Me]$^+$

a

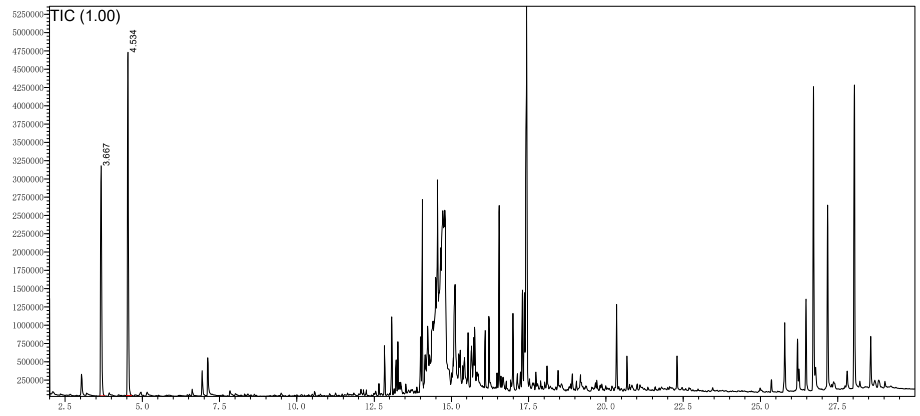

b

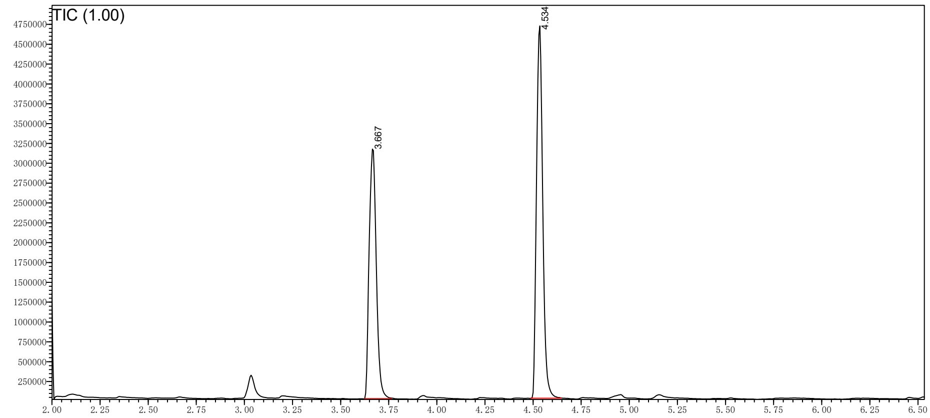

Supplementary Figure 6. a, GC-MS total ion current map of hydrogenated sample. **b**, GC-MS ion current map of hydrogenated sample (2-6.3 min).

a

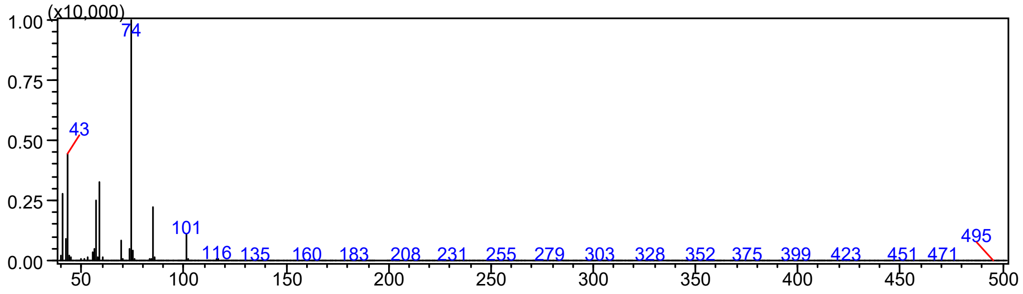

b

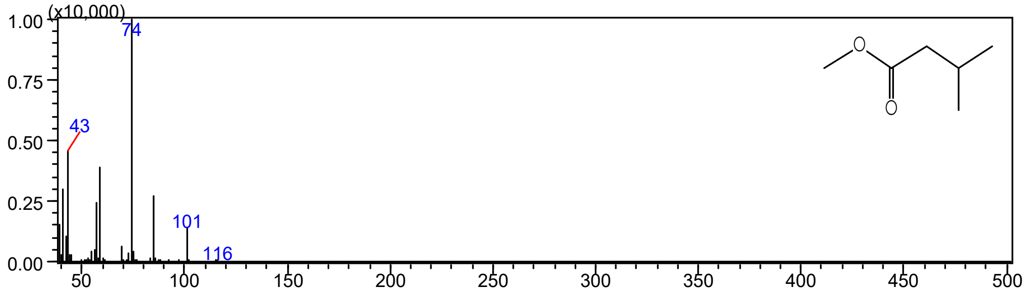

Supplementary Figure 7. a, Mass spectrum of methyl isopentanoate in 3.67 min (retention time). **b**, Database search results.

a

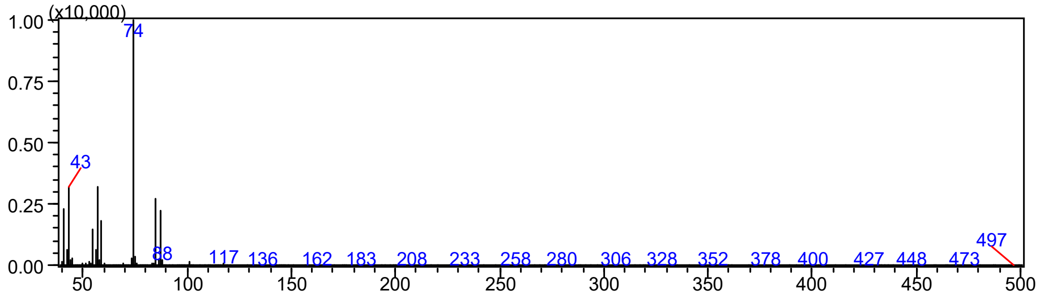

b

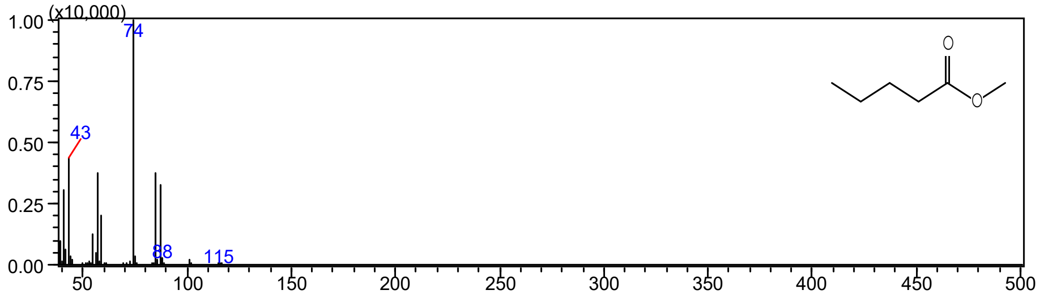

Supplementary Figure 8. a, Mass spectrum of methyl pentanoate in 4.51 min (retention time). **b**, Database search results.

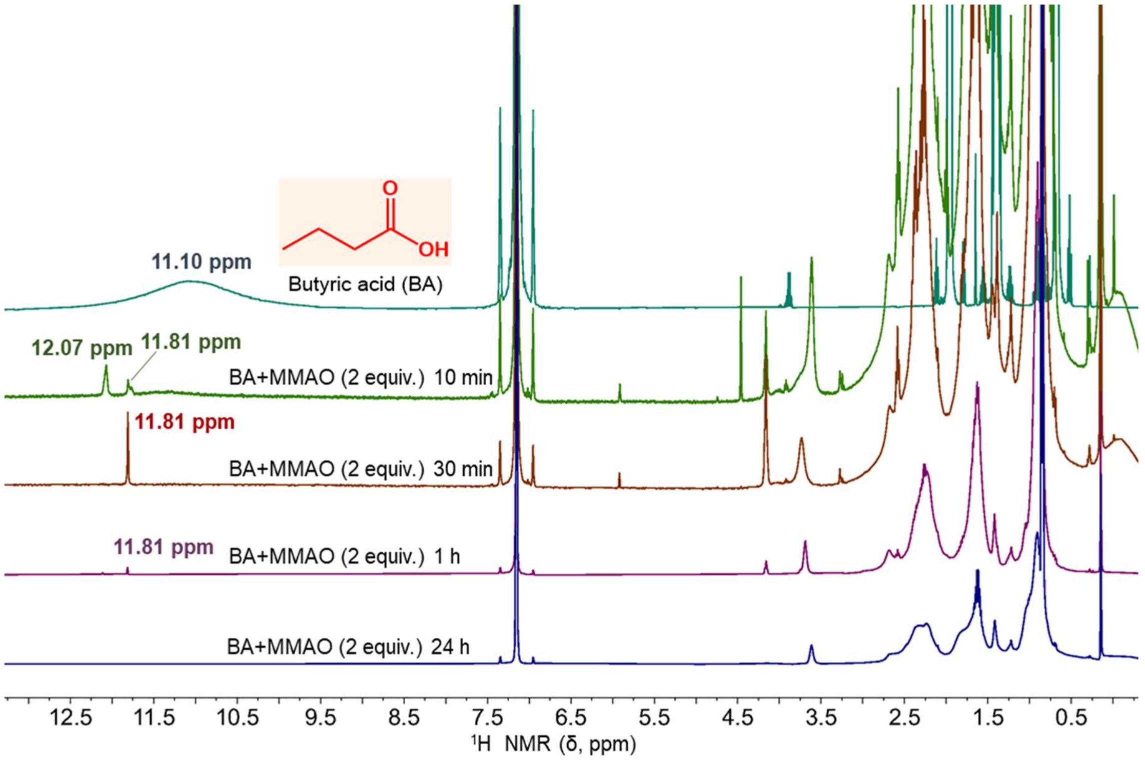

Supplementary Figure 9. ^{1}H NMR tracking MMAO + BA *in situ* reaction (C_6D_6, 25 ^{o}C).

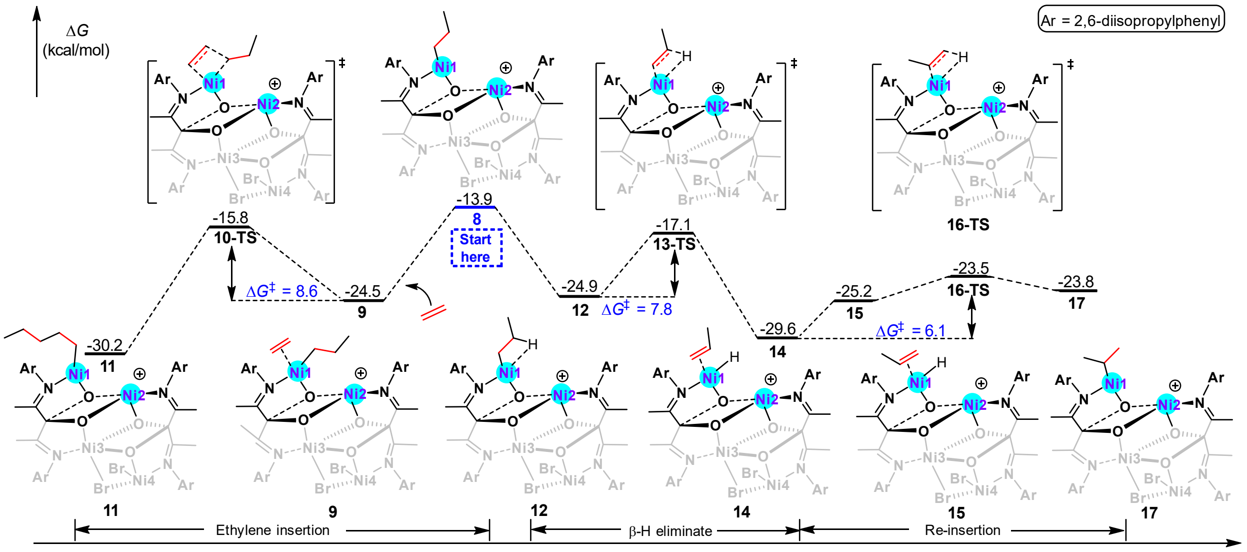

Supplementary Figure 10. Computed energy profile for the Ni mediated β-H elimination and re-insertion on the basis of ethylene insertion product **8**. Although the reinsertion is slightly endergonic by 5.8 kcal/mol, the subsequent ethylene enchainment could make this event eventually exergonic because of successive C−C bond formations.

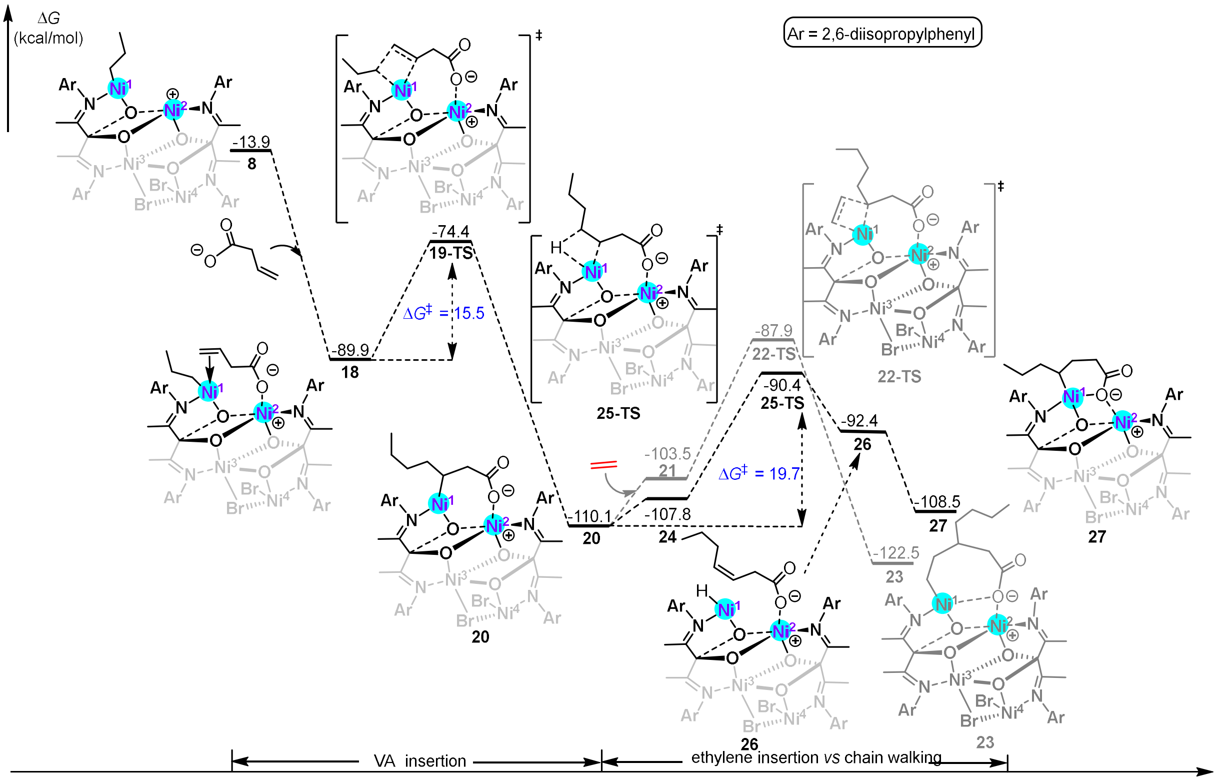

Supplementary Figure 11. Computed energy profiles for Ni mediated copolymerization of ethylene with VA anion. The energies are relative to the cationic active species with Ni-Me moiety and corresponding monomers.

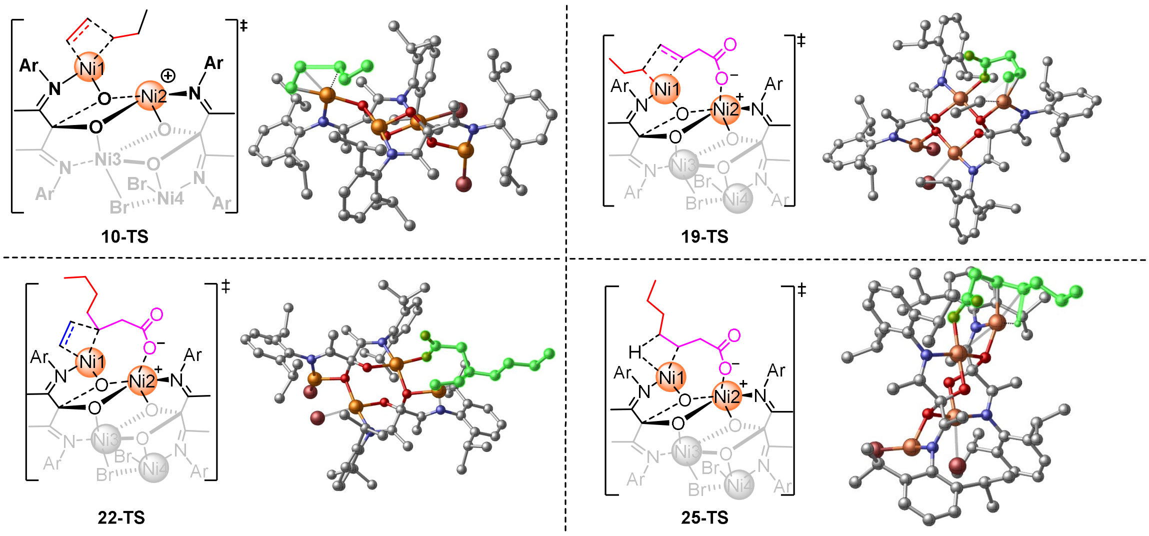

Supplementary Figure 12. Geometric structures of **10-TS**, **19-TS**, **22-TS**, **25-TS**.

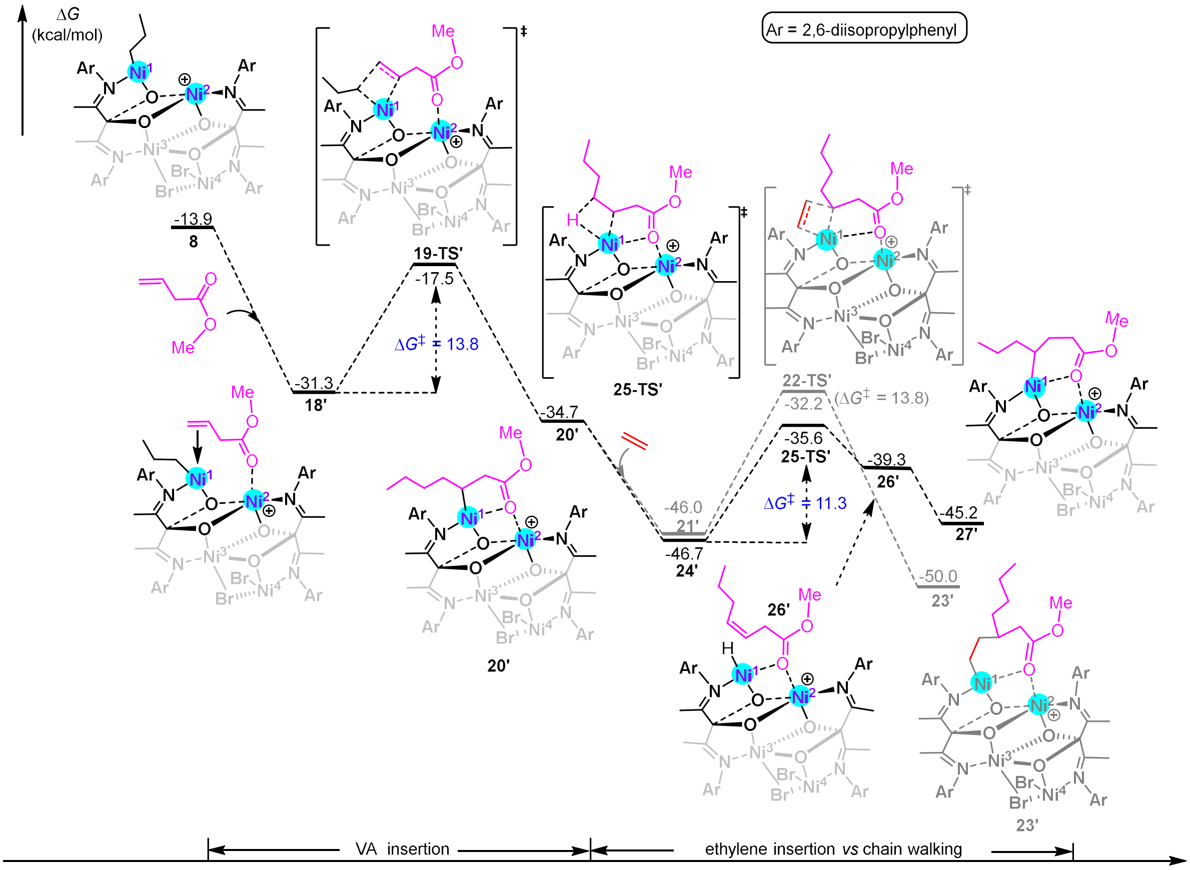

Supplementary Figure 13. Computed energy profiles for Ni mediated copolymerization of ethylene with methyl vinylacetate (MVA). The energies are relative to the cationic active species with Ni-Me moiety and corresponding monomers.

a ^{1}H NMR spectrum of **6**

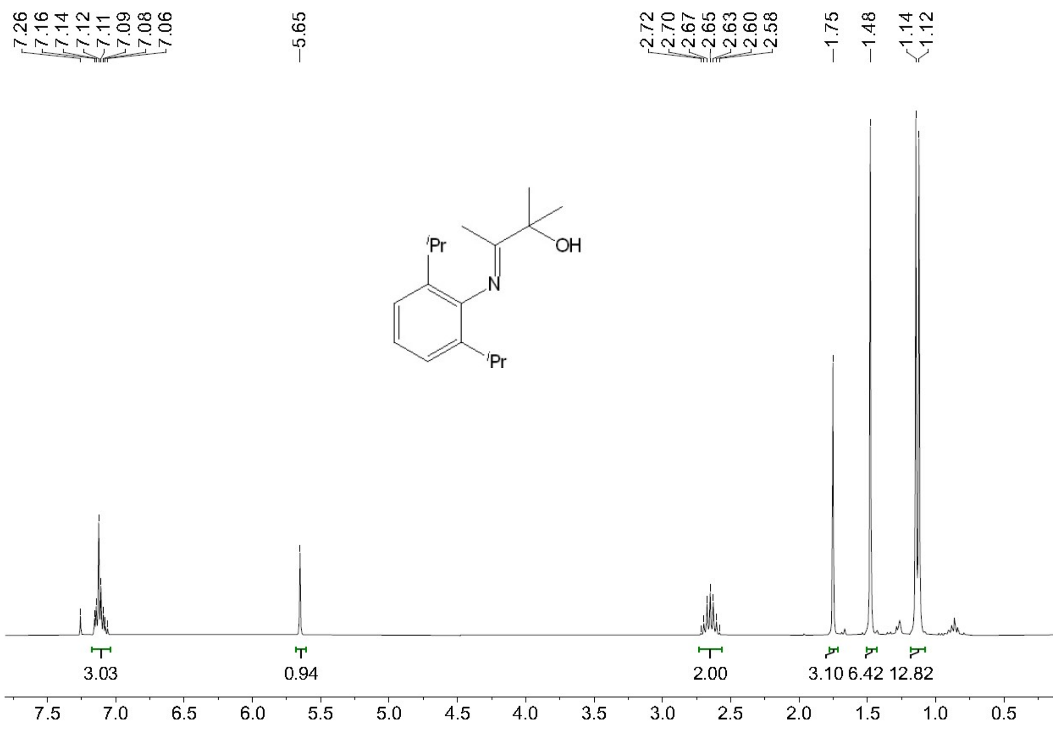

b ^{13}C NMR spectrum of **6**

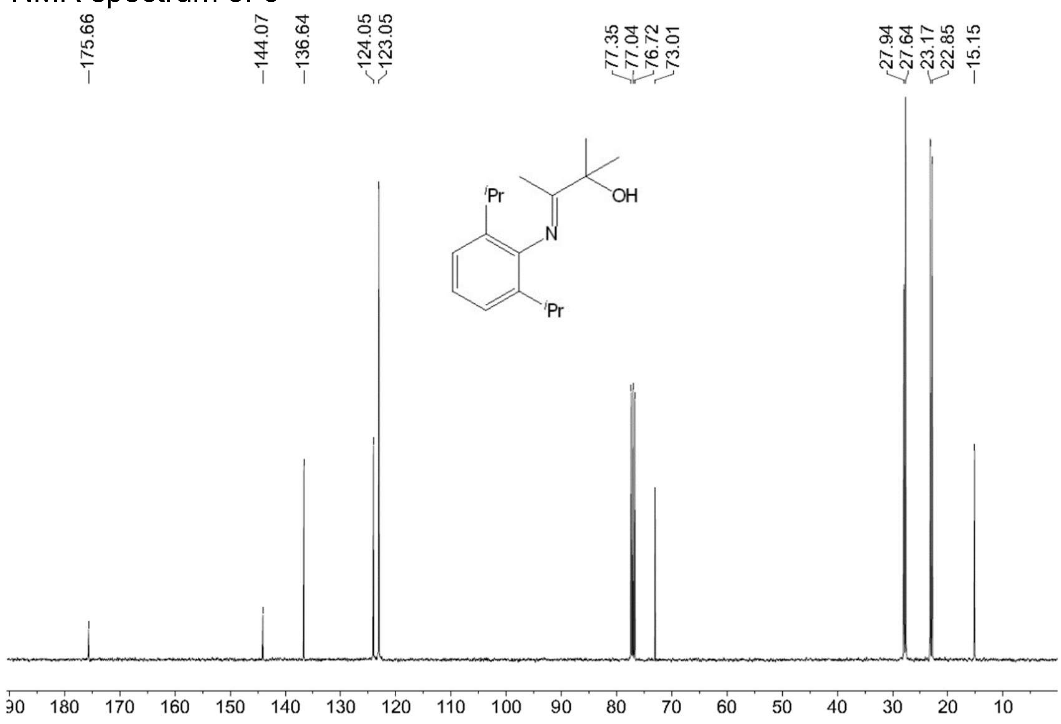

Supplementary Figure 14. ^{1}H NMR (**a**) and ^{13}C NMR (**b**) spectra of **6**, in $CDCl_3$.

a ^{1}H NMR spectrum of **28**

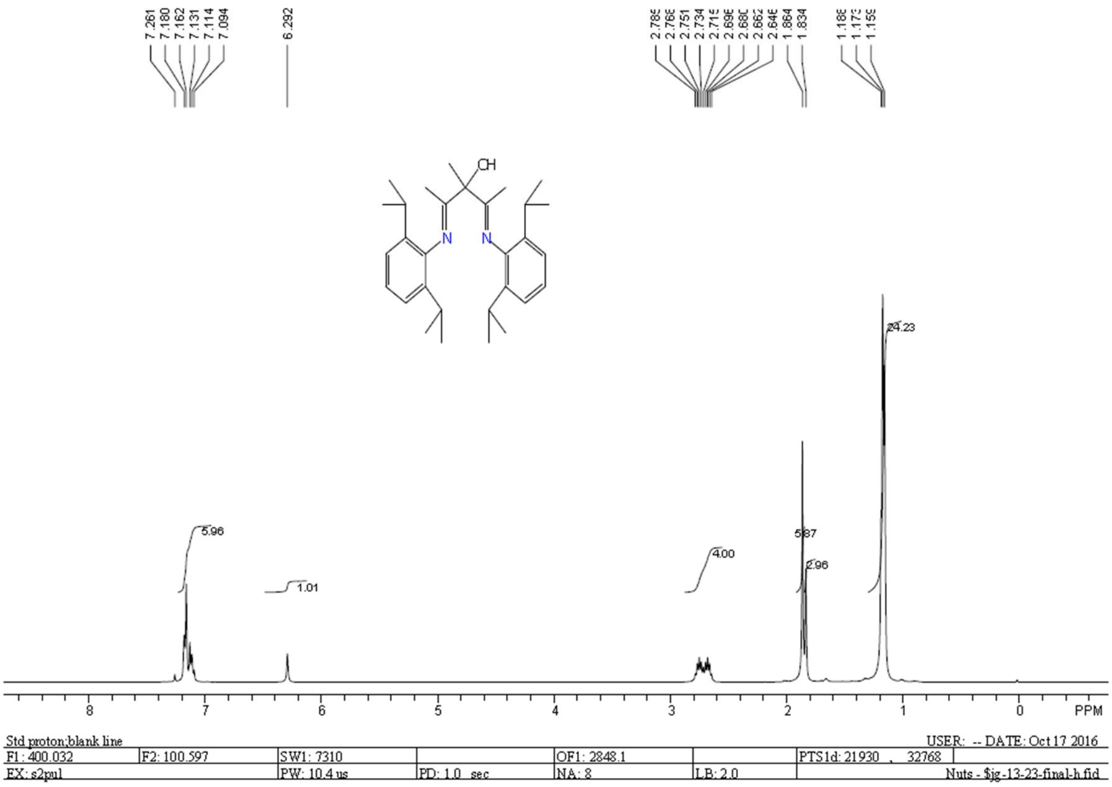

b ^{13}C NMR spectrum of **28**

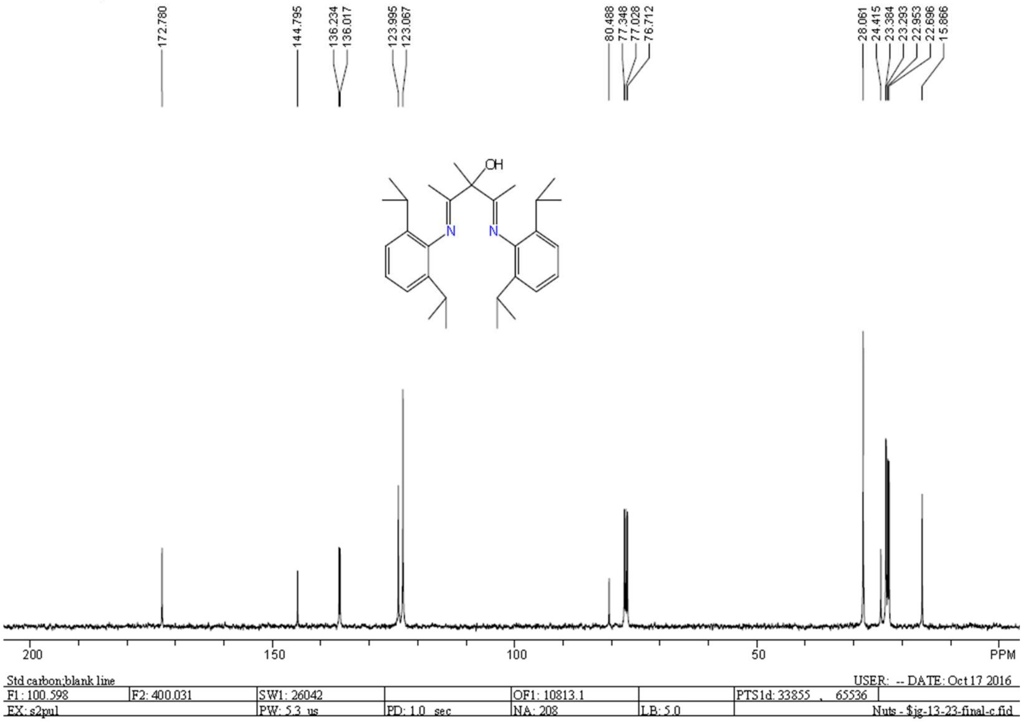

Supplementary Figure 15. ^{1}H NMR (**a**) and ^{13}C NMR (**b**) spectra of **28**, in $CDCl_3$.

a ^{1}H NMR spectrum of the polyethylene sample from Supplementary Table 1, Entry 7

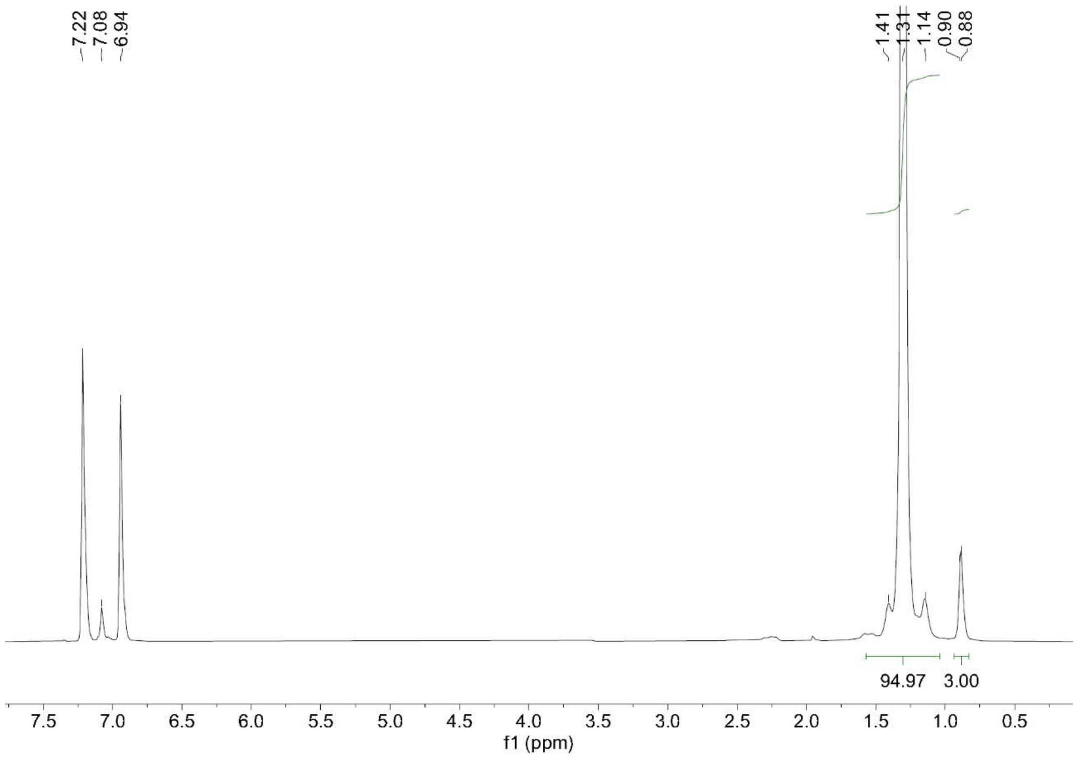

b ^{13}C NMR spectrum of the polyethylene sample from Supplementary Table 1, Entry 7

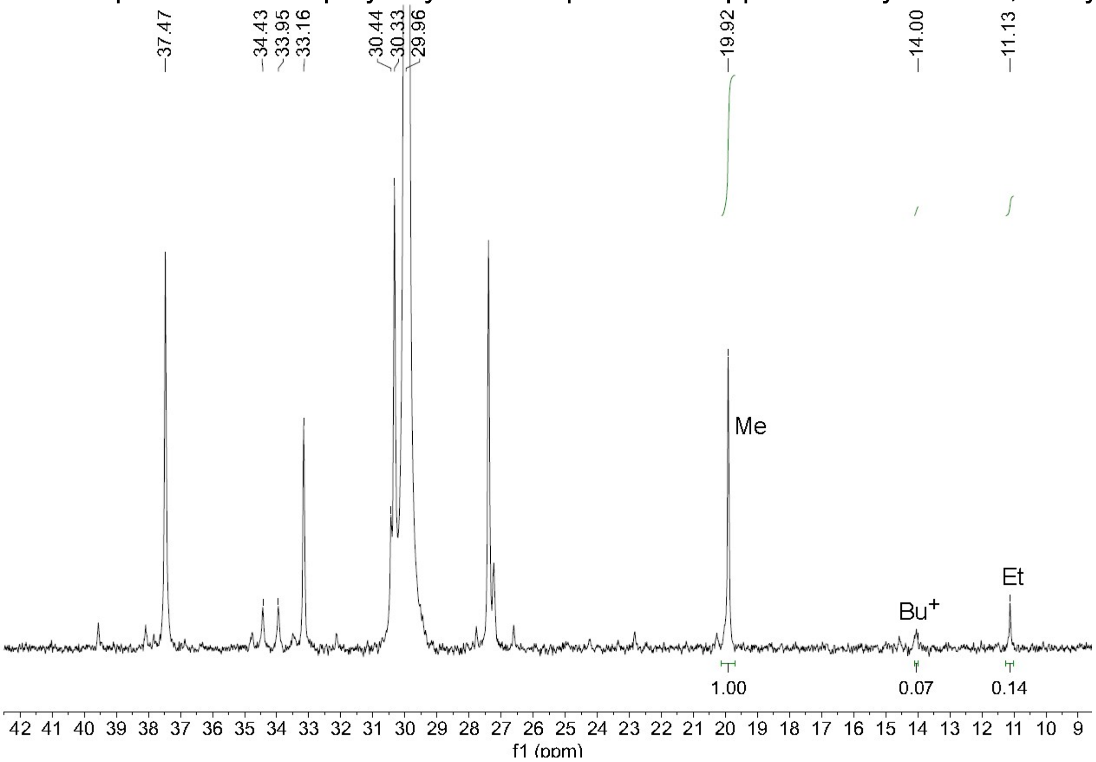

Supplementary Figure 16. NMR spectra of polyethylene sample from Supplementary Table 1, Entry 7. (**a**) ^{1}H NMR (**b**) ^{13}C NMR, in 1,2-dichlorobenzene-*d*$_4$,110 °C.

a ^{1}H NMR spectrum of the poly(ethylene-*co*-VA) sample from Table 2, Entry 1.

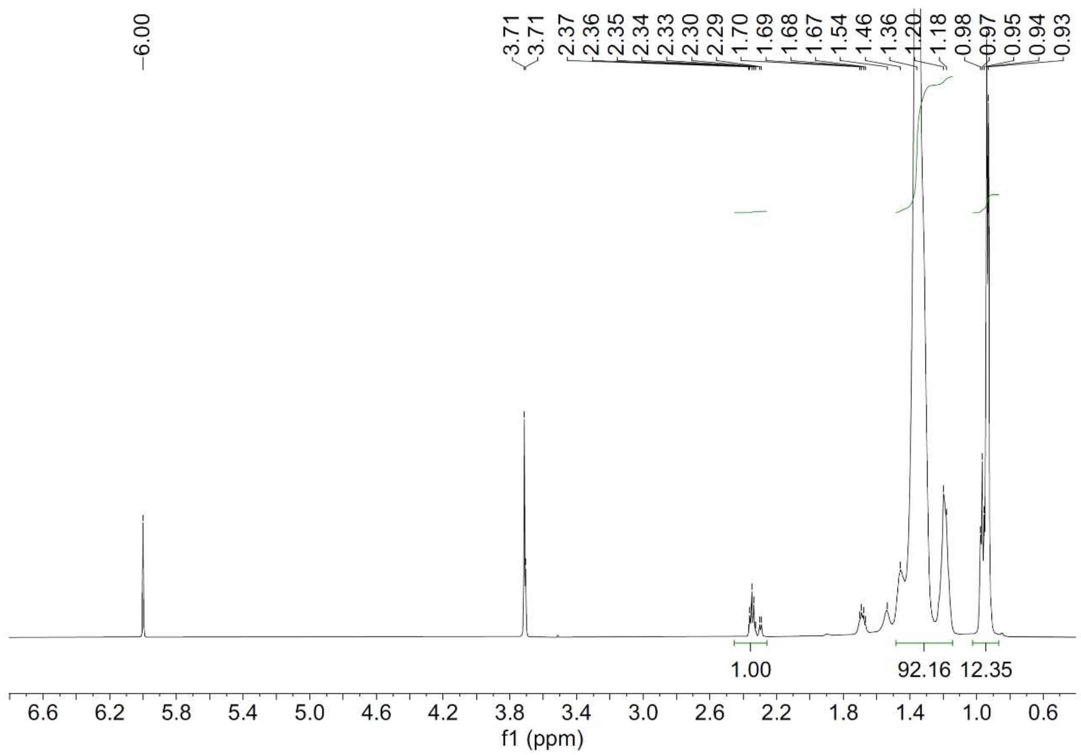

b ^{13}C NMR spectrum of the poly(ethylene-*co*-VA) sample from Table 2, Entry 1.

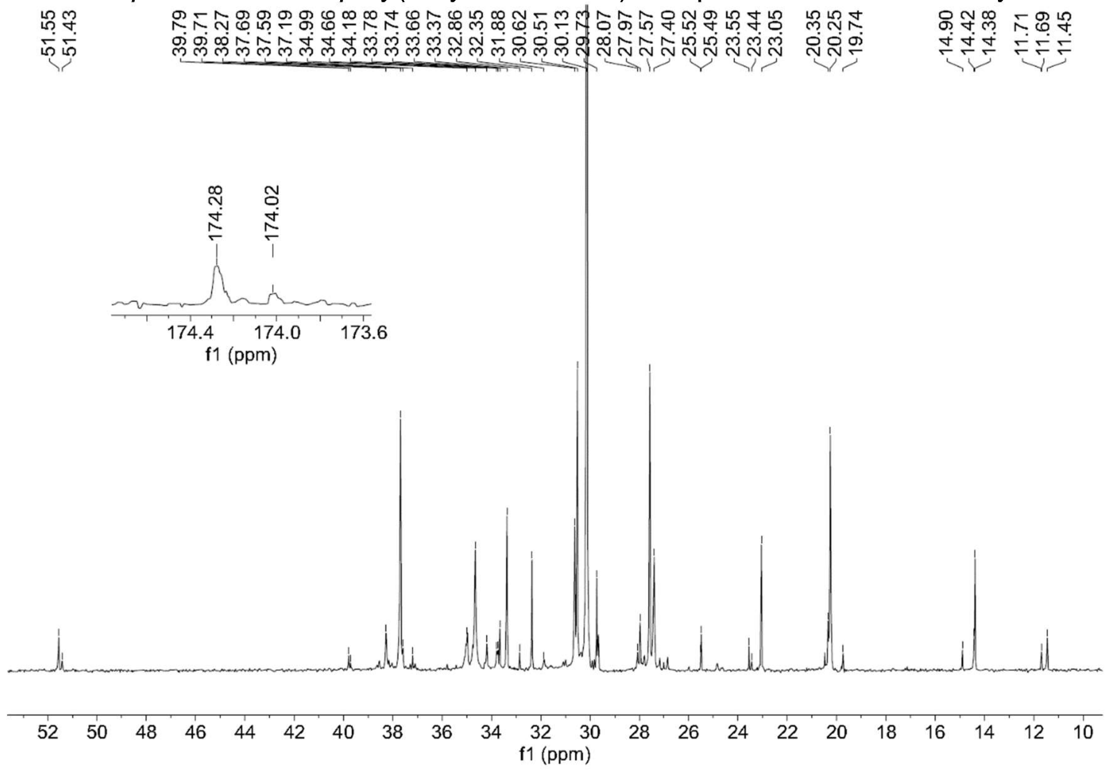

c DEPT 135 spectrum of the poly(ethylene-*co*-VA) sample from Table 2, Entry 1.

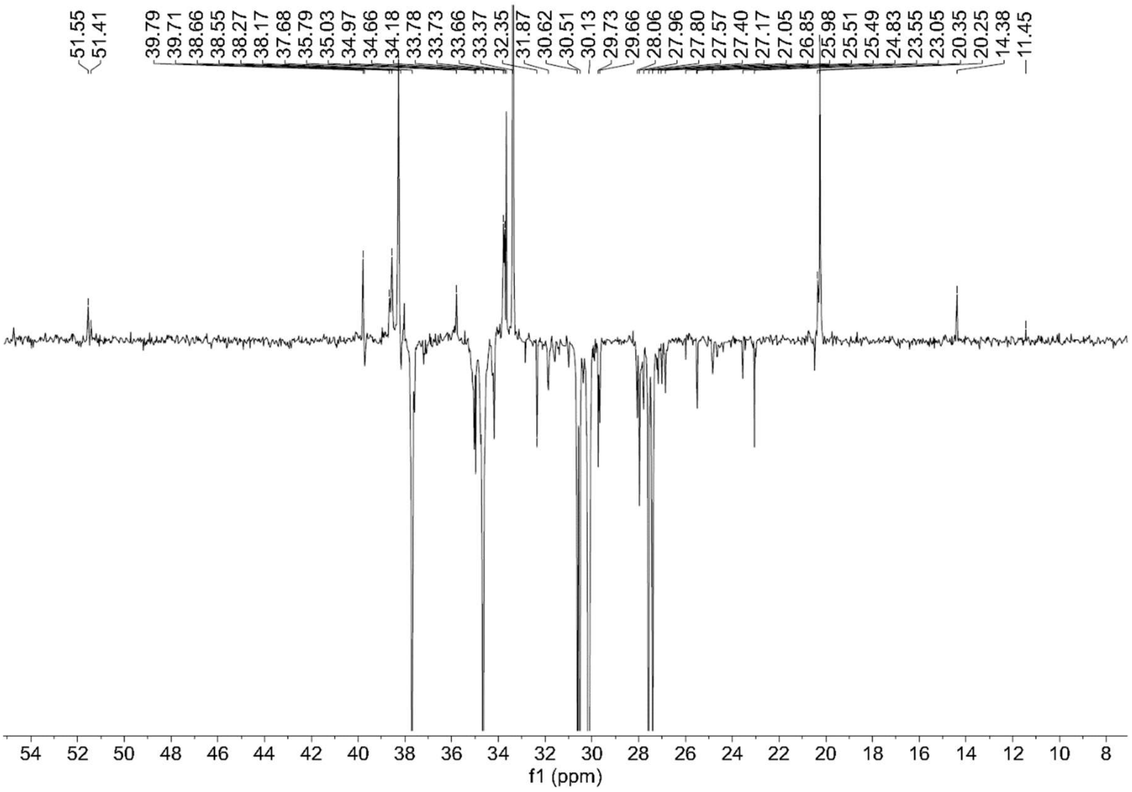

d COSY spectrum of the poly(ethylene-*co*-VA) sample from Table 2, Entry 1.

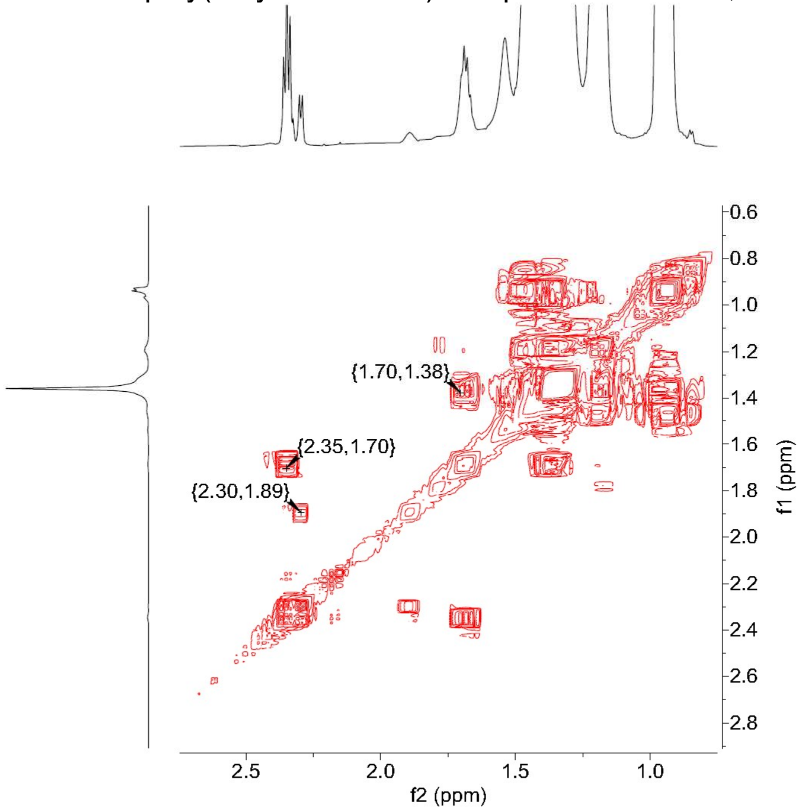

e HSQC spectrum of the poly(ethylene-*co*-VA) sample from Table 2, Entry 1.

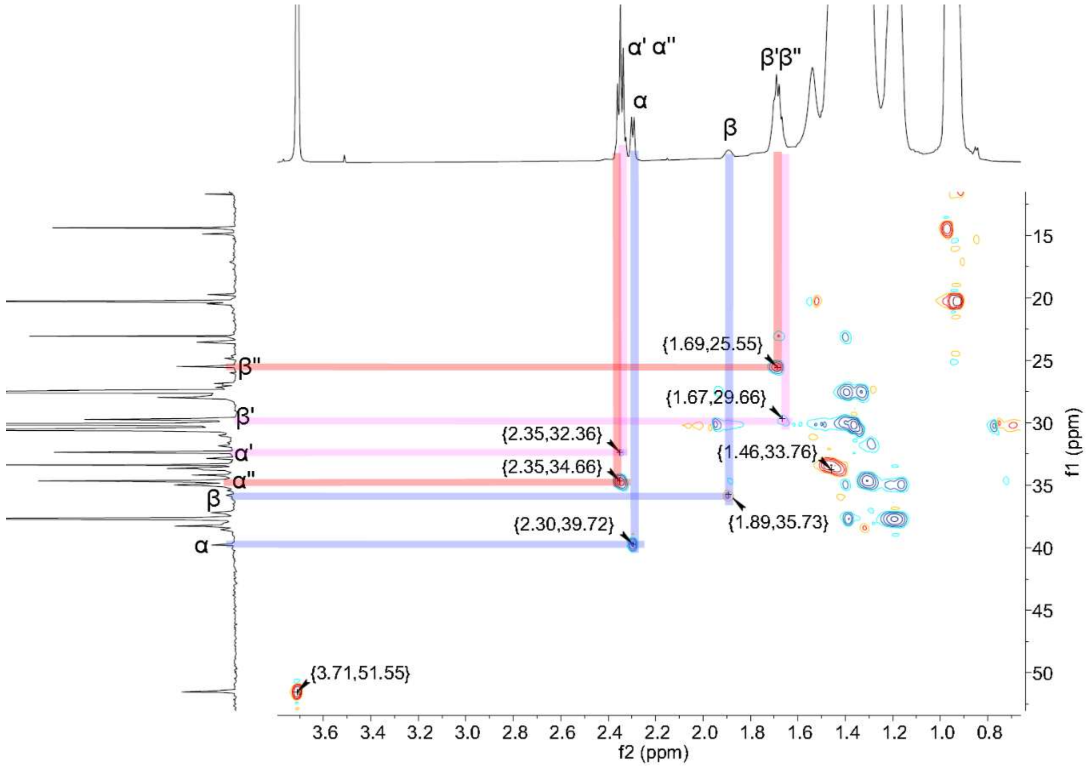

f HMBC spectrum of the poly(ethylene-*co*-VA) sample from Table 2, Entry 1.

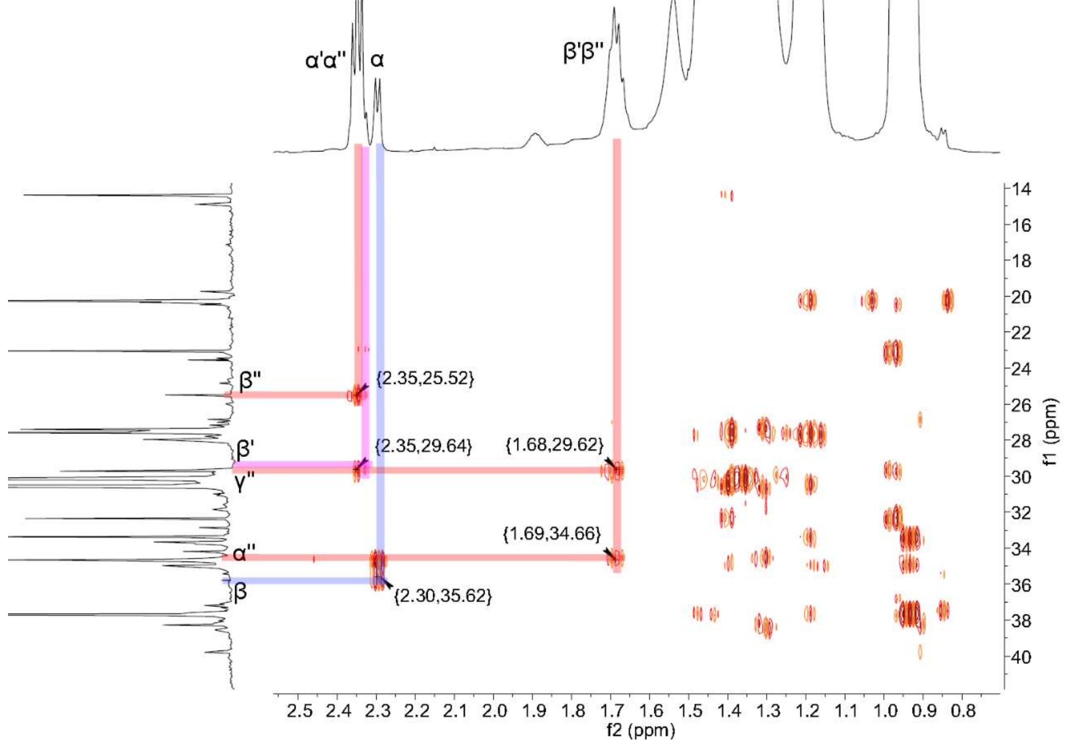

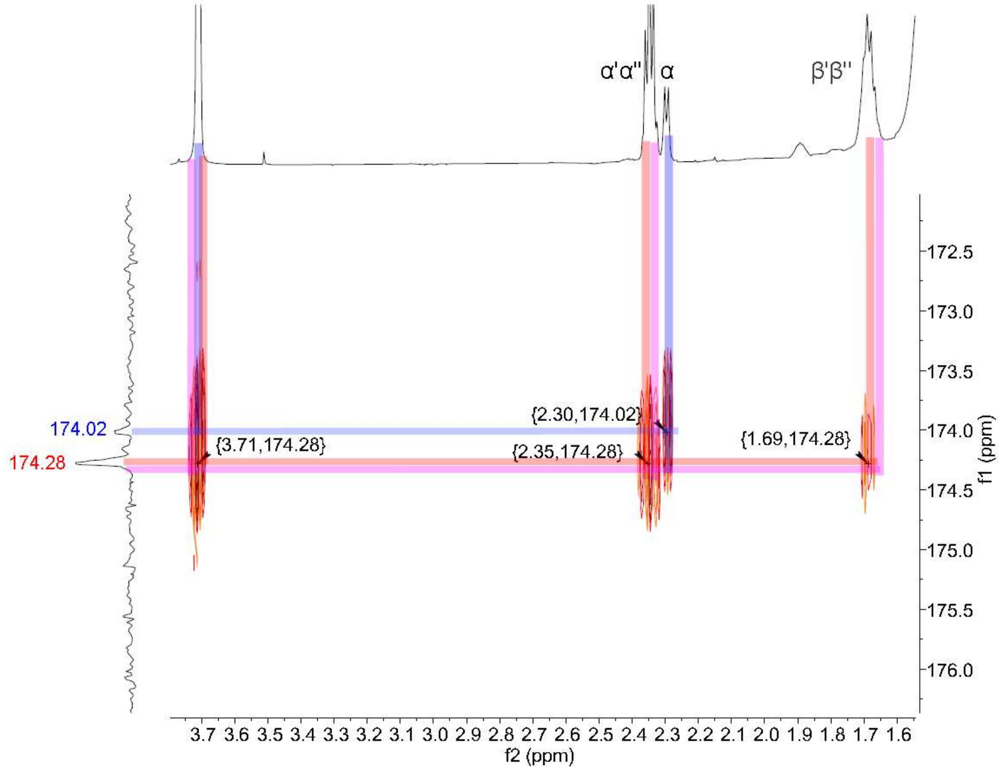

NMR data

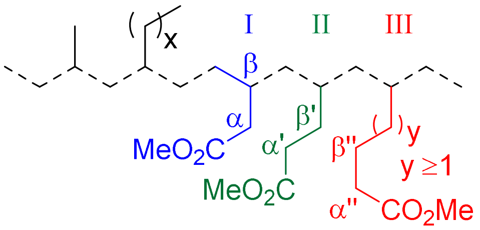

^{13}C NMR(^{1}H NMR)

	I	II	III
α	39.72(2.30)	32.36(2.35)	34.66(2.35)
β	35.73(1.89)	29.64(1.67)	25.55(1.69)
-COO*Me*	51.43(3.71)	51.55(3.71)	51.55(3.71)
-*C*OOMe	174.02	174.28	174.28

Supplementary Figure 17. NMR spectra of the poly(ethylene-*co*-VA) sample from Table 2, Entry 1 (2.1 mol% VA incorporation, after methyl esterification). (**a**) ^{1}H NMR (**b**) ^{13}C NMR (**c**) DEPT 135 (**d**) H-H COSY (**e**) HSQC (**f**) HMBC, in 1,1,2,2-tetrachloroethane-d_2,110 °C.

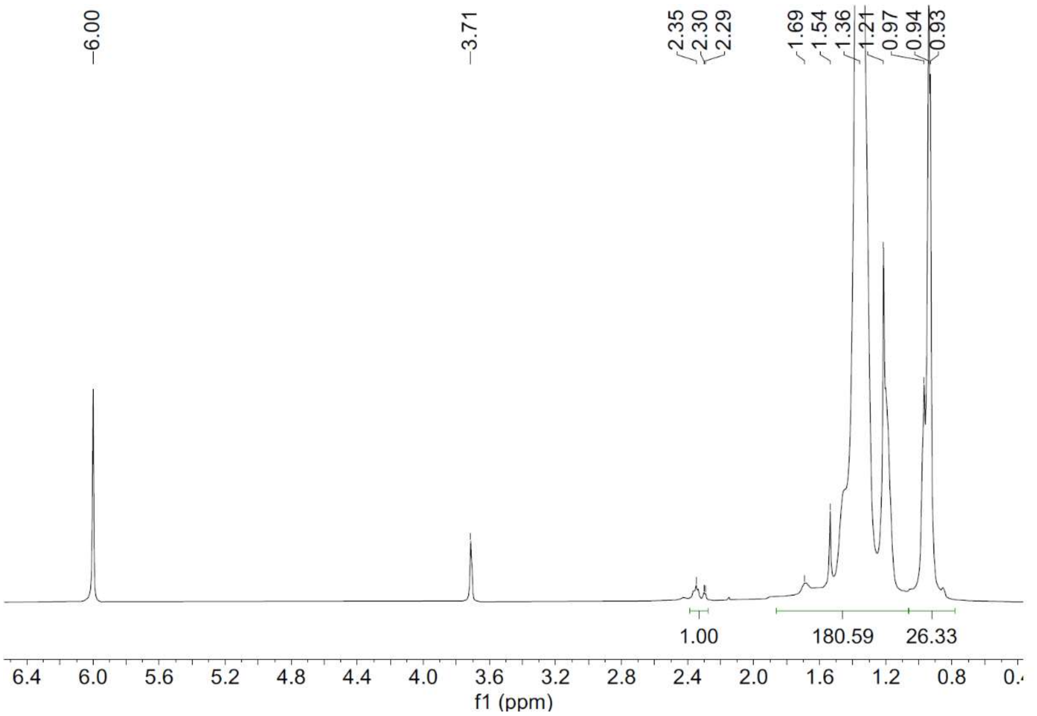

Supplementary Figure 18. ^{1}H NMR spectrum of the poly(ethylene-*co*-MVA) sample from Table 2, Entry 9 (1.1 mol% MVA incorporation, after methyl esterification), in 1,1,2,2-tetrachloroethane-d_2,110 °C.

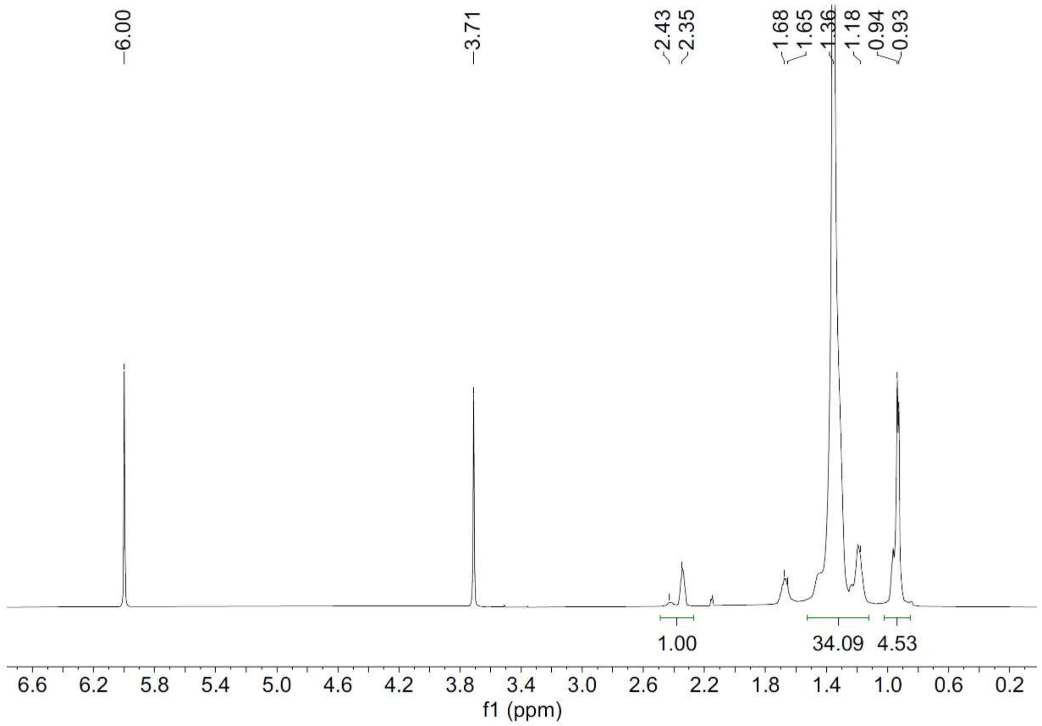

Supplementary Figure 19. ^{1}H NMR spectrum of the poly(ethylene-*co*-PA) sample from Table 2, Entry 10 (5.8 mol% PA incorporation, after methyl esterification), in 1,1,2,2-tetrachloroethane-d_2,110 °C.

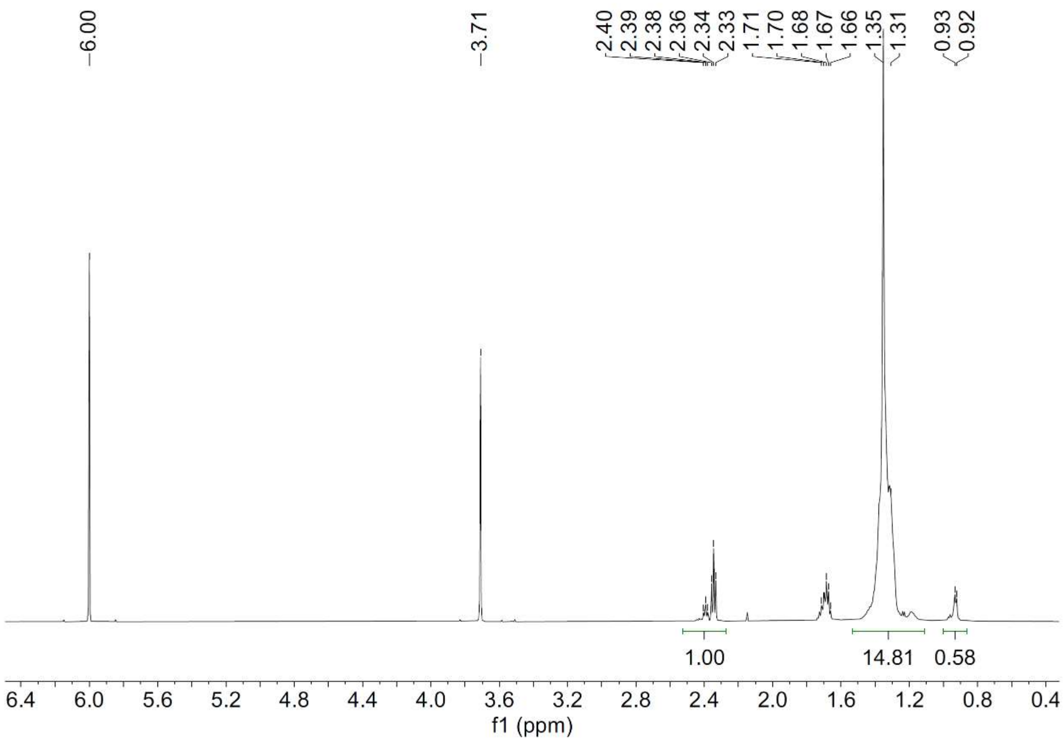

Supplementary Figure 20. ^{1}H NMR spectrum of the poly(ethylene-*co*-UA) sample from Table 2, Entry 14 (21.5 mol% UA incorporation, before methyl esterification), in 1,2-dichlorobenzene-d_4,110 °C.

a ^{1}H NMR spectrum of the poly(ethylene-*co*-HAA) sample from Table 2, Entry 16.

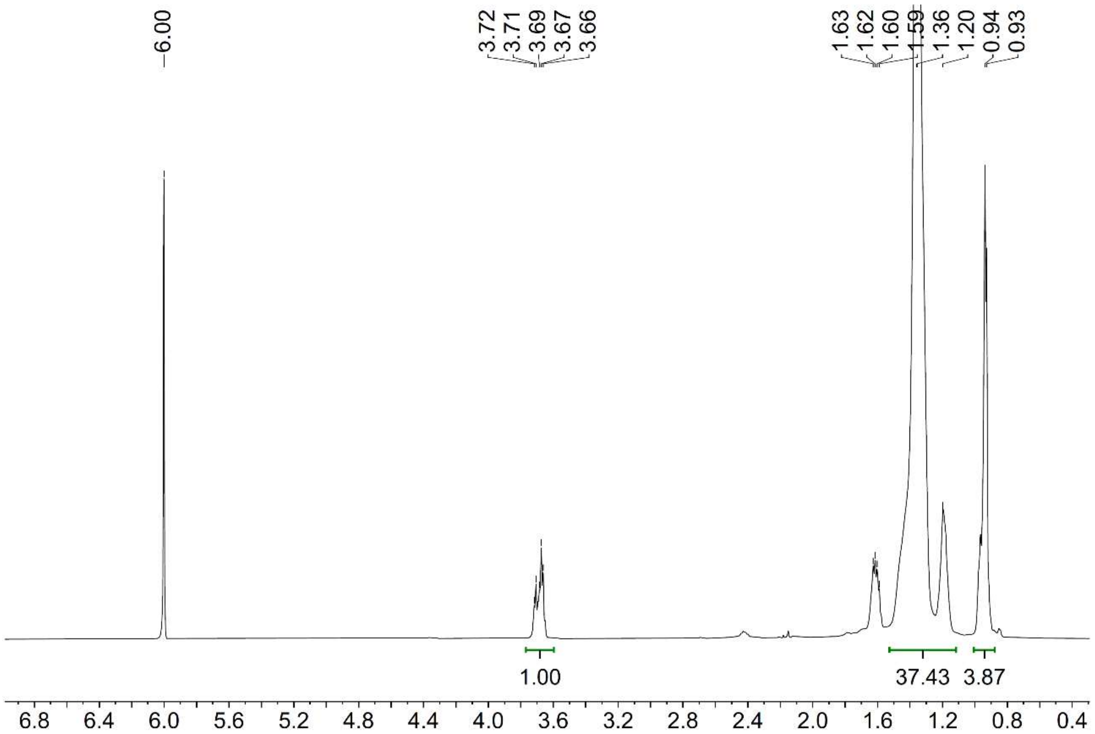

b ^{13}C NMR spectrum of the poly(ethylene-*co*-HAA) sample from Table 2, Entry 16.

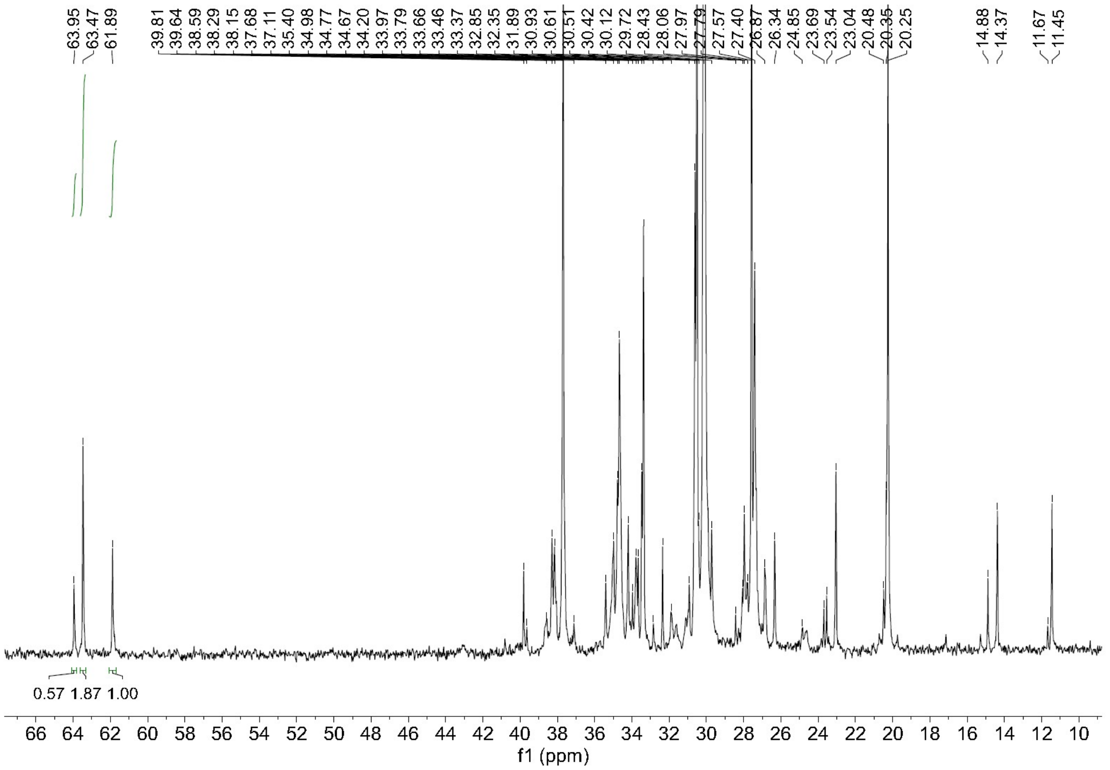

c DEPT 135 spectrum of the poly(ethylene-*co*-HAA) sample from Table 2, Entry 16.

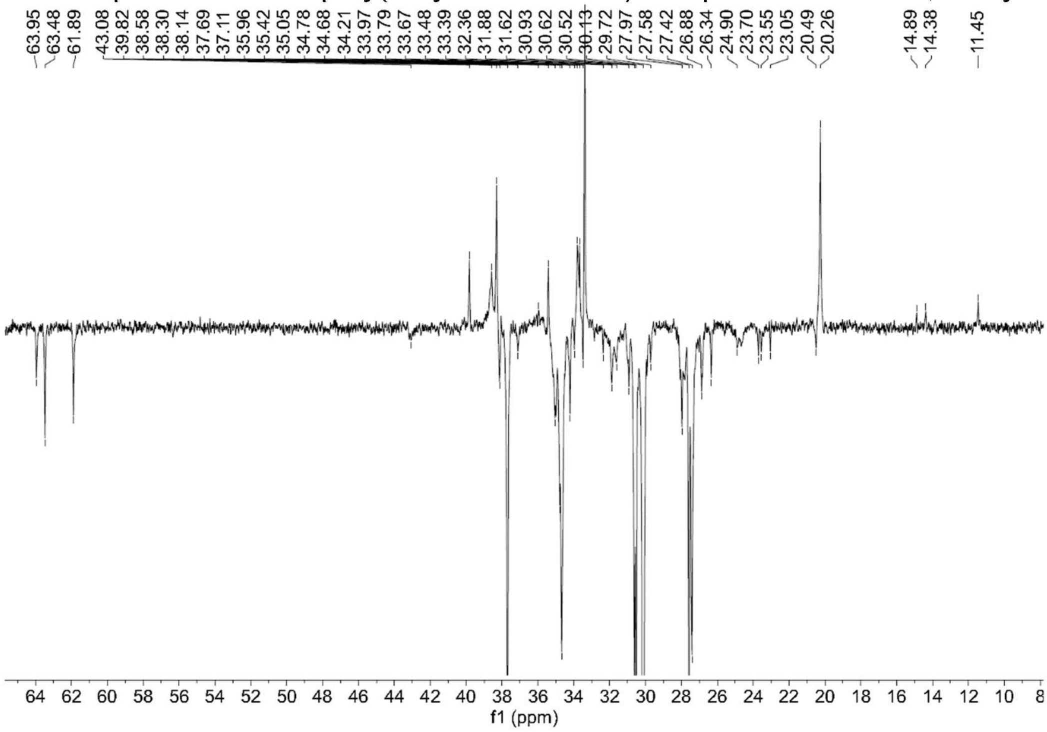

d COSY spectrum of the poly(ethylene-*co*-HAA) sample from Table 2, Entry 16.

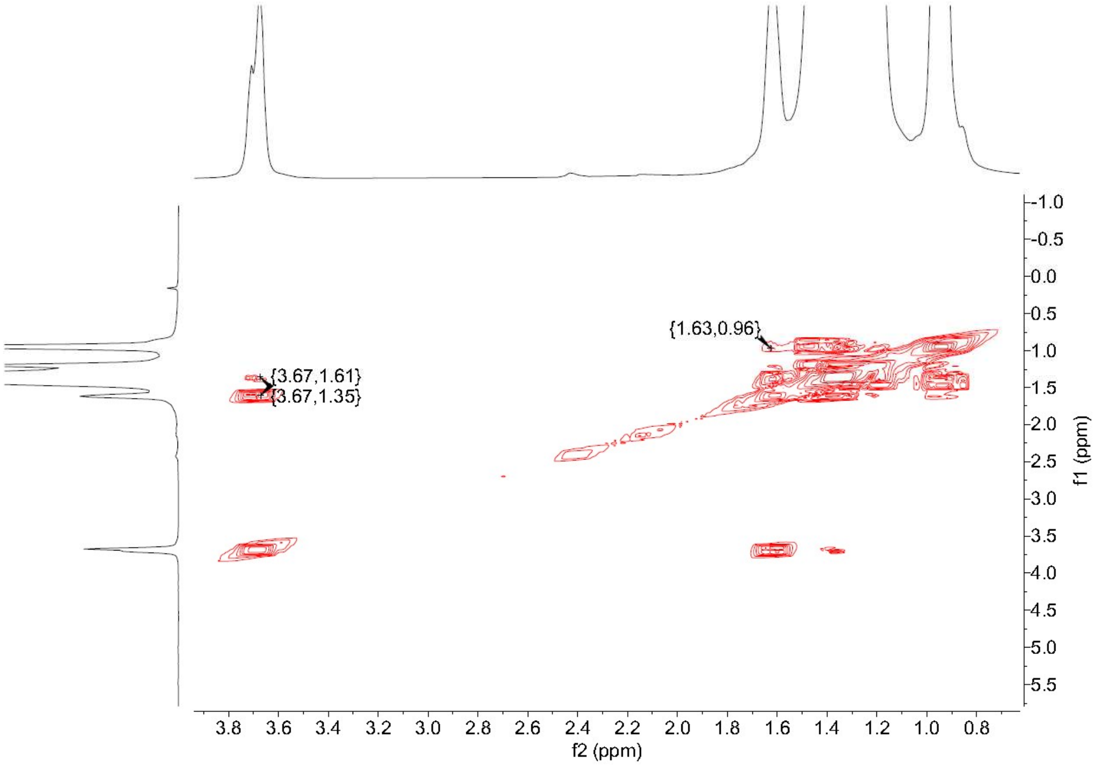

e HSQC spectrum of the poly(ethylene-*co*-HAA) sample from Table 2, Entry 16.

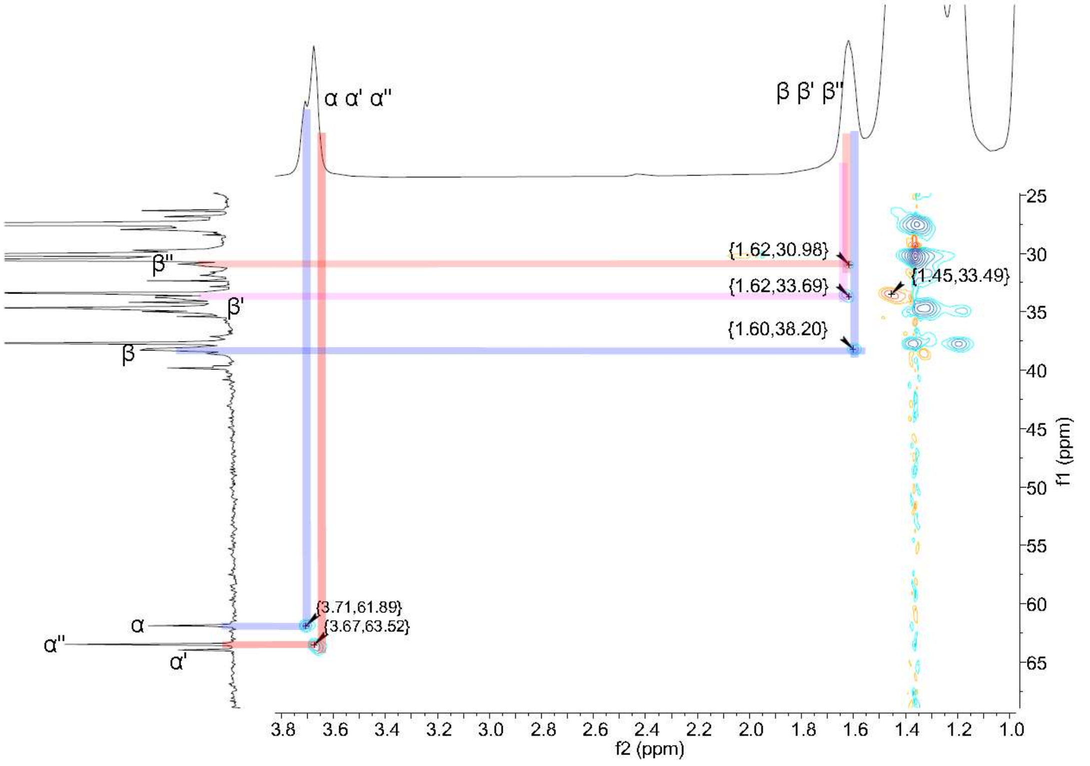

f HMBC spectrum of the poly(ethylene-*co*-HAA) sample from Table 2, Entry 16.

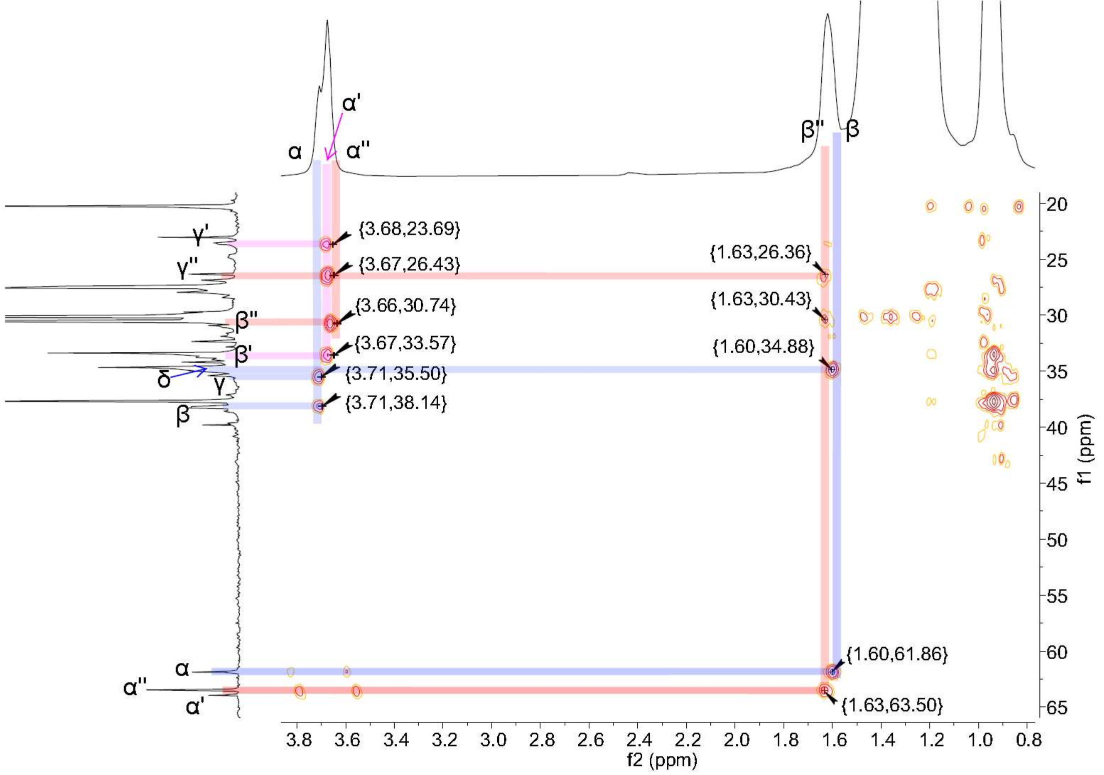

NMR data

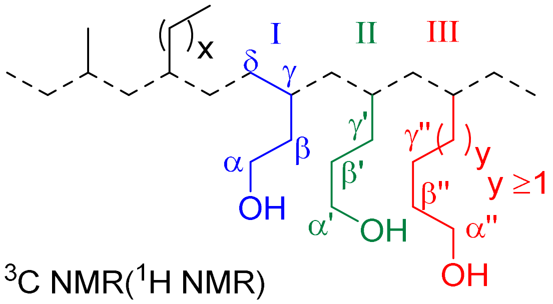

^{13}C NMR(^{1}H NMR)

	I	II	III
α	61.89(3.71)	63.95(3.67)	63.50(3.67)
β	38.14(1.60)	33.69(1.62)	30.98(1.63)
γ	35.50	23.89	26.30
δ	34.88	–	–

Supplementary Figure 21. NMR spectra of the poly(ethylene-*co*-HAA) sample from Table 2, Entry 16 (5.3 mol% HAA incorporation). (**a**) ^{1}H NMR (**b**) ^{13}C NMR (**c**) DEPT 135 (**d**) H-H COSY (**e**) HSQC (**f**) HMBC, in 1,1,2,2-tetrachloroethane-*d*$_2$,110 °C.

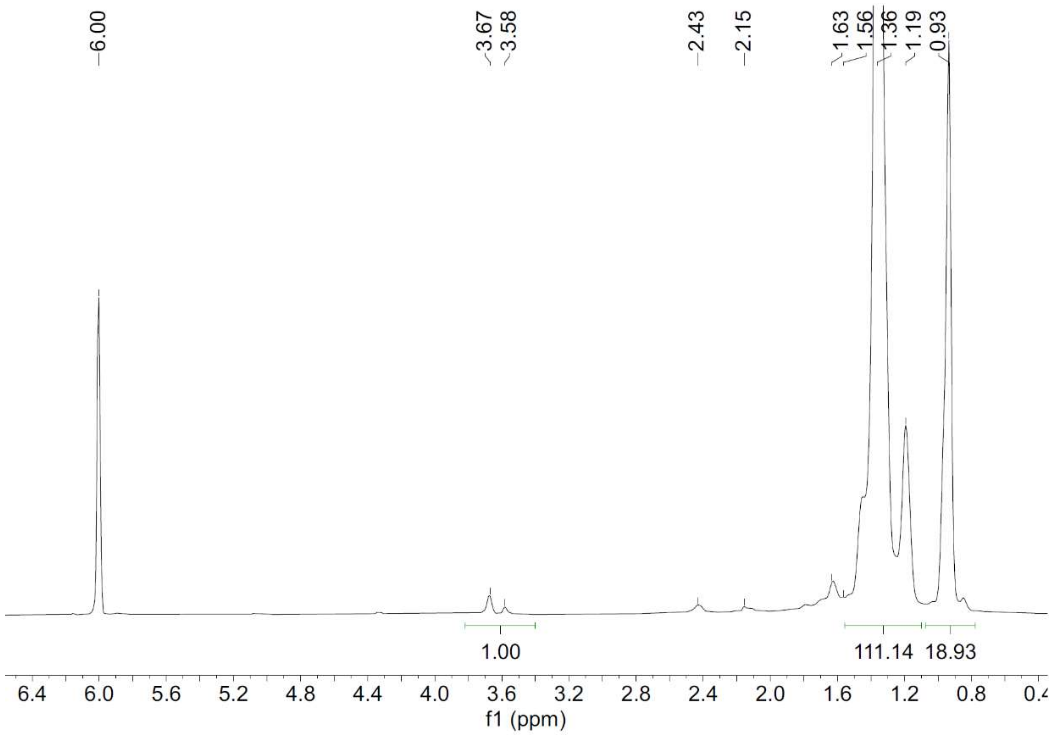

Supplementary Figure 22. ^{1}H NMR spectrum of the poly(ethylene-*co*-allyl alcohol) sample from Table 2, Entry 18 (1.8 mol% allyl alcohol incorporation), in 1,1,2,2-tetrachloroethane-d_2,110 °C.

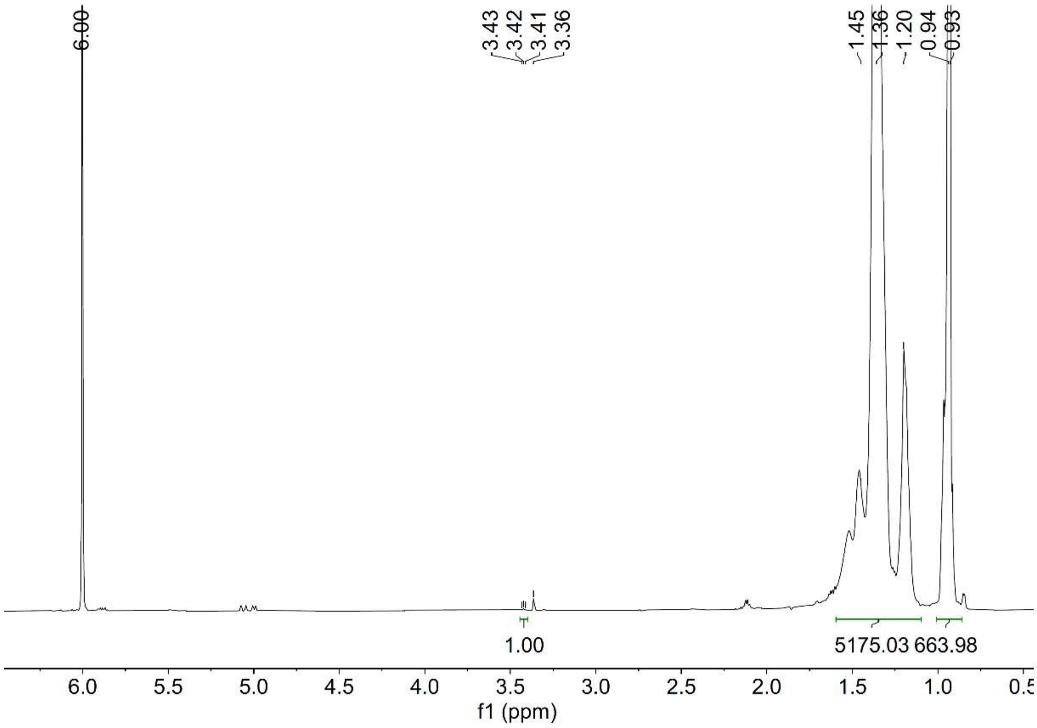

Supplementary Figure 23. ^{1}H NMR spectrum of the poly(ethylene-*co*-AME) sample from Table 2, Entry 19 (0.04 mol% AME incorporation), in 1,1,2,2-tetrachloroethane-d_2,110 °C.

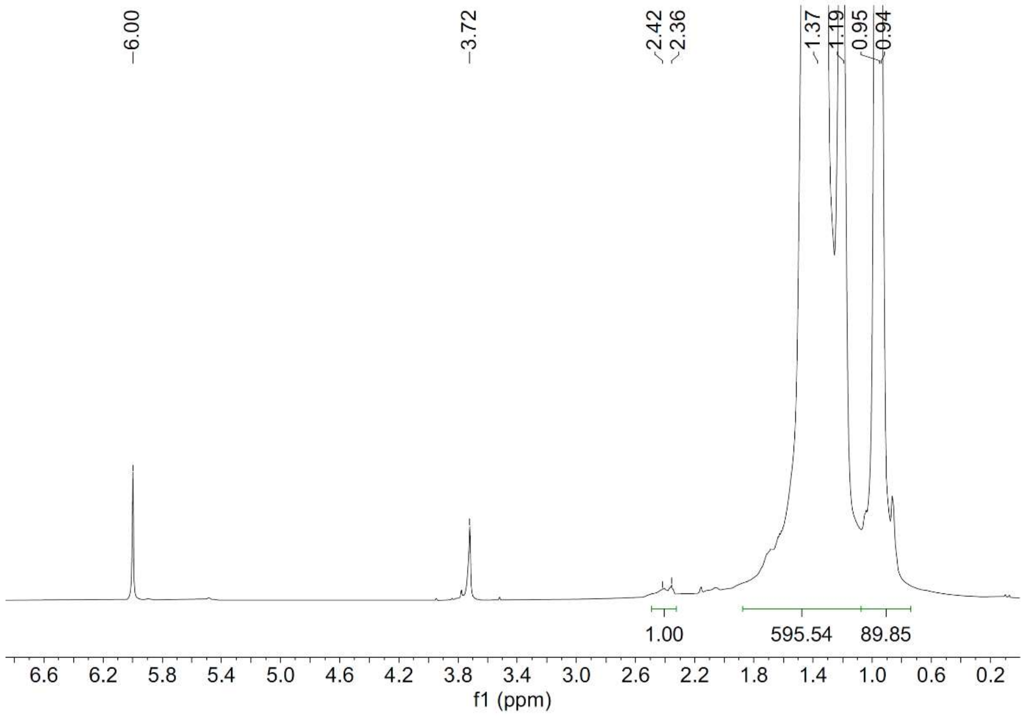

Supplementary Figure 24. NMR spectra of the poly(ethylene-*co*-AA) sample from Table 2, Entry 20 (0.3 mol% AA incorporation, after methyl esterification).

a ^{1}H NMR spectrum of the poly(ethylene-*co*-AA) sample from Table 3, Entry 7.

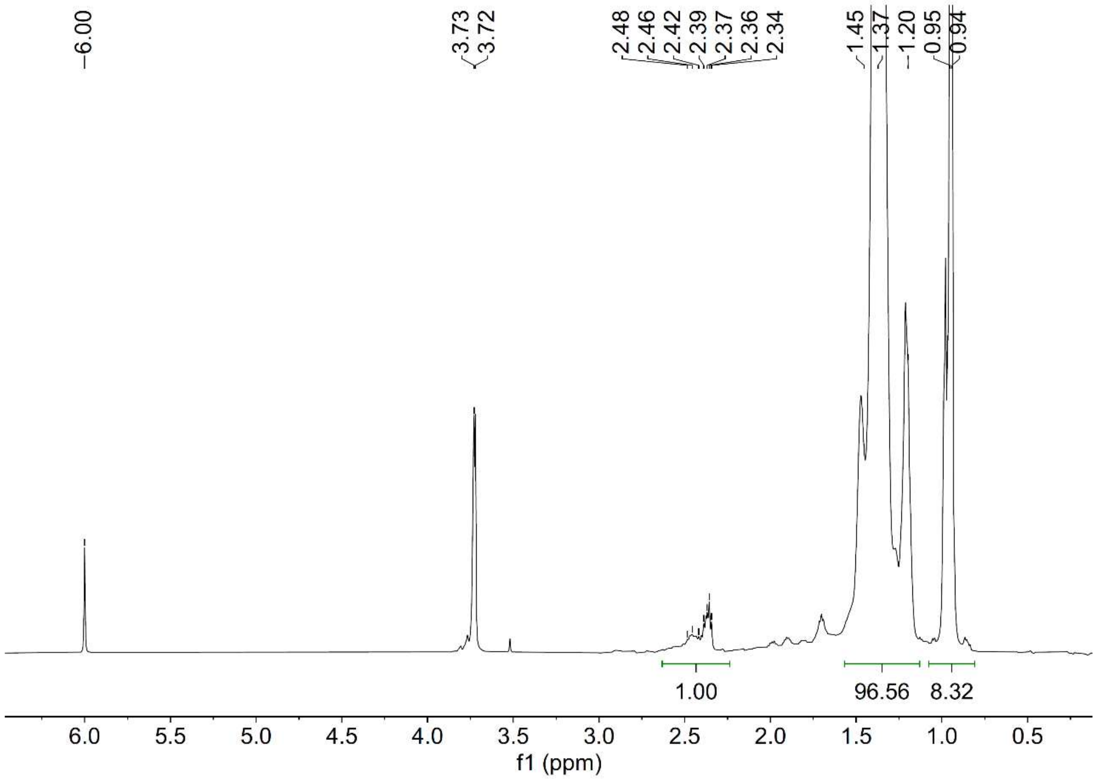

b ^{13}C NMR spectrum of the poly(ethylene-*co*-AA) sample from Table 3, Entry 7.

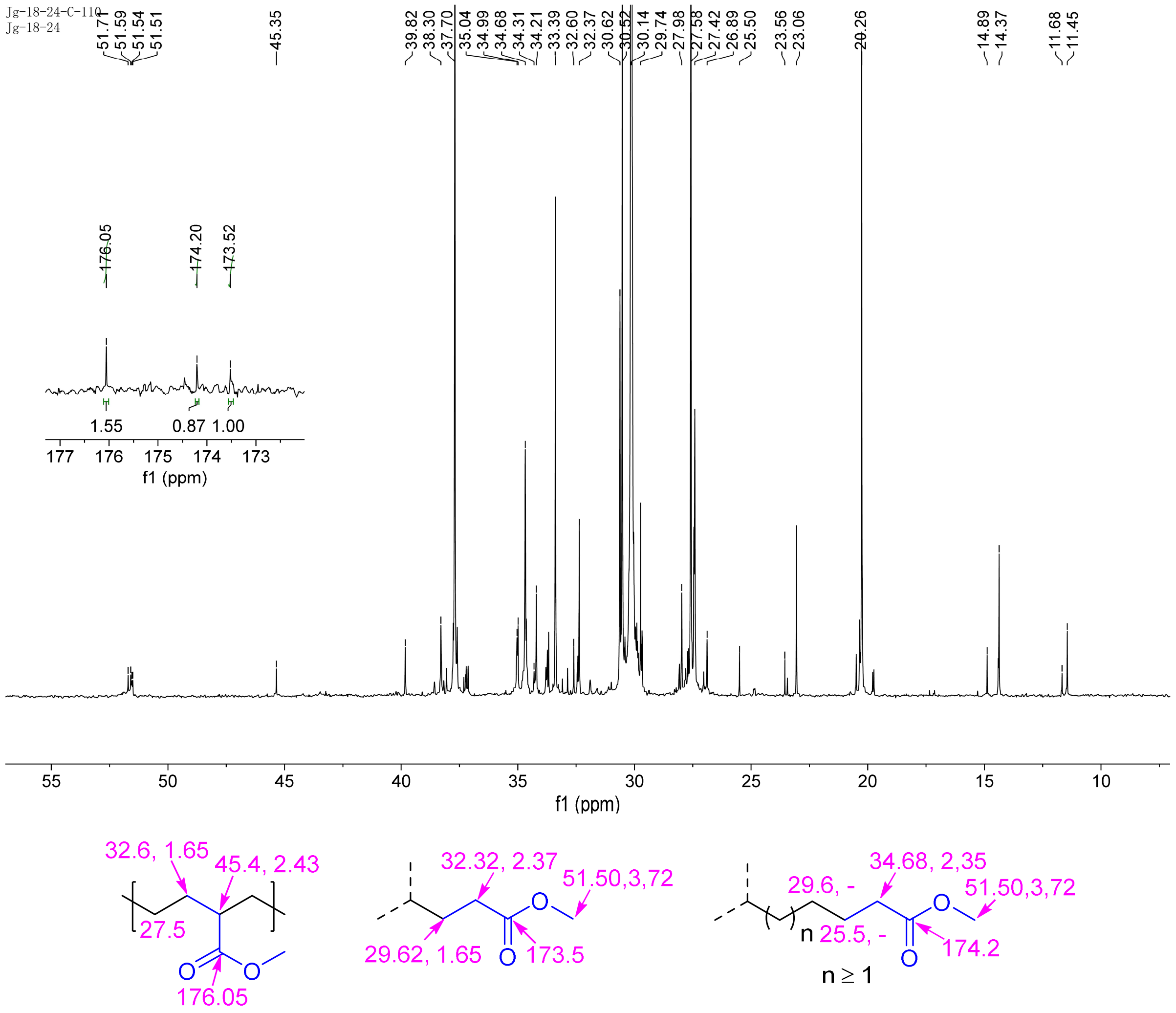

Supplementary Figure 25. NMR spectra of the poly(ethylene-*co*-AA) sample from Table 3, Entry 7 (2.1 mol% AA incorporation, after methyl esterification). (**a**) ^{1}H NMR (**b**) ^{13}C NMR, in 1,1,2,2-tetrachloroethane-d_2,110 °C.

MW Averages

Mp: 451532	Mn: 309896	Mv: 550364	Mw: 597011
Mz: 1030132	Mz+1: 1592938	PD: 1.9265	

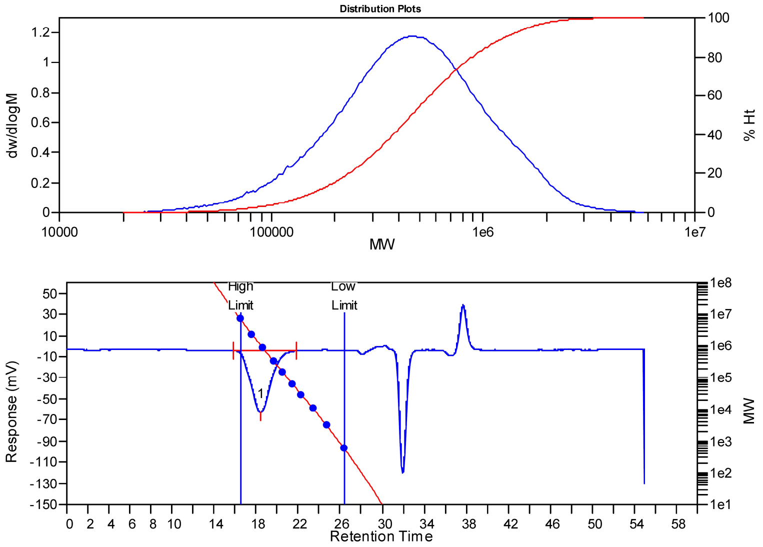

Supplementary Figure 26. GPC trace of the polyethylene sample from Supplementary Table 1, Entry 7.

MW Averages

Mp: 18645	Mn: 9855	Mv: 28171	Mw: 32593
Mz: 83002	Mz+1: 151923	PD: 3.3073	

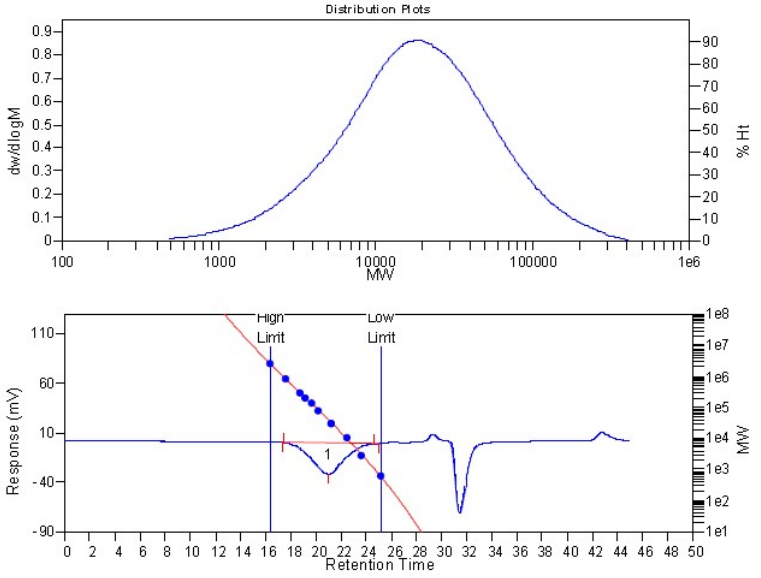

Cirrus GPC Version 3.4 Page 1

Supplementary Figure 27. GPC traces of the poly(ethylene-*co*-VA) sample from Table 2, Entry 1.

MW Averages

Mp: 12727	Mn: 5132	Mv: 13265	Mw: 15221
Mz: 38498	Mz+1: 78893	PD: 2.9659	

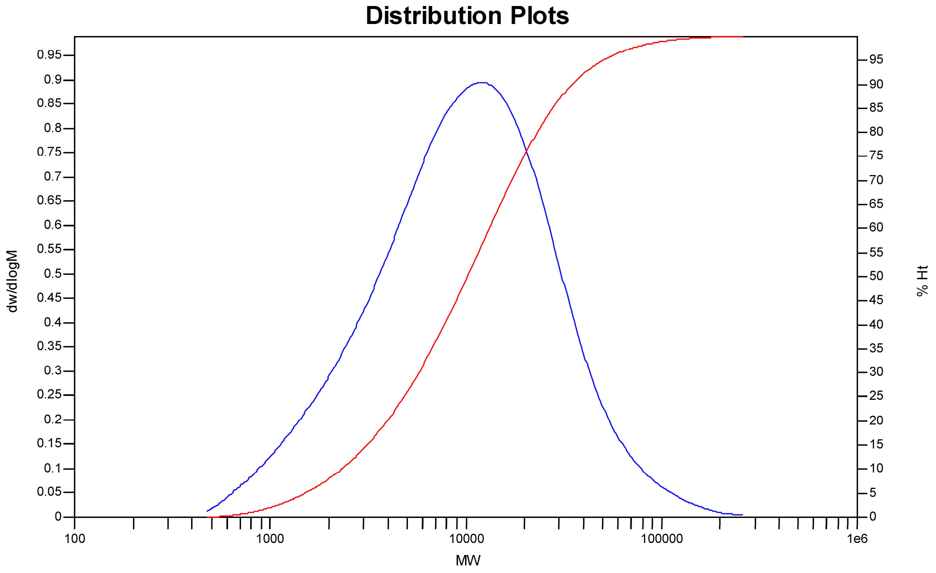

Supplementary Figure 28. GPC traces of the poly(ethylene-*co*-MVA) sample from Table 2, Entry 9.

MW Averages

Mp: 27495	Mn: 8154	Mv: 27155	Mw: 30738
Mz: 65308	Mz+1: 111499	PD: 3.7697	

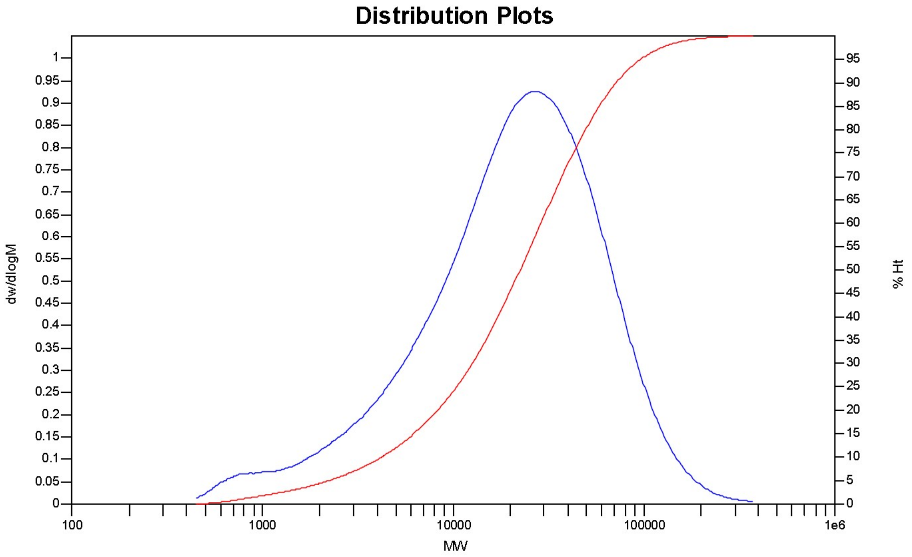

Supplementary Figure 29. GPC traces of the poly(ethylene-*co*-PA) sample from Table 2, Entry 10.

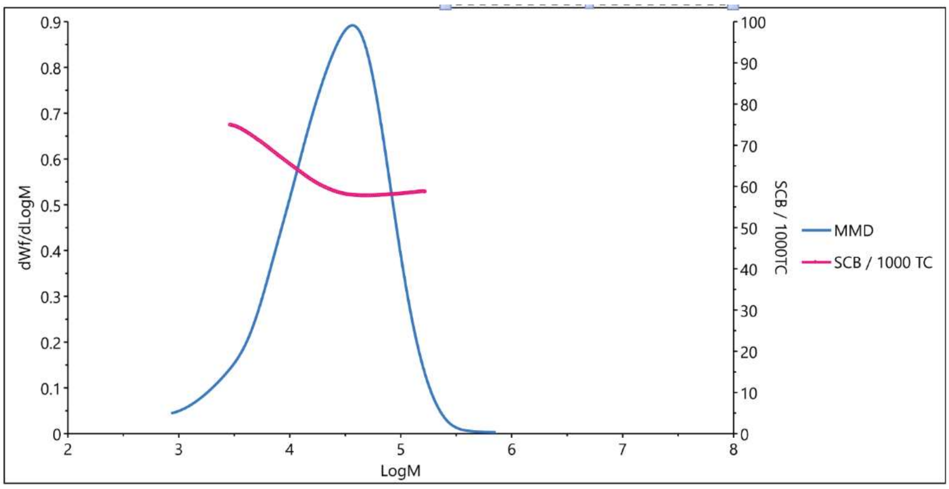

Results

Mw	39109	g/mol
Mn	12112	g/mol
Mw / Mn	3.23	
Mz	85897	g/mol
Mp	36599	g/mol
Mv	34082	g/mol
IVmwd	0.6919	dL/g
Bulk CH3 / 1000C	63.918	
Corrected Bulk CH3 / 1000C	61.6	CH3/1000TC corr

Calibrations

Calib Flow Marker	26.94	mL
Calib Mass constant	0.70	mL·V/mg

Performance

Flow Marker	27.09	mL
Mass recovery	78	%
Plate Count	22625	

Supplementary Figure 30. GPC traces of the poly(ethylene-*co*-HAA) sample from Table 2, Entry 16.

MW Averages

Mp: 7166	Mn: 2621	Mv: 7248	Mw: 8316
Mz: 19308	Mz+1: 33176	PD: 3.1728	

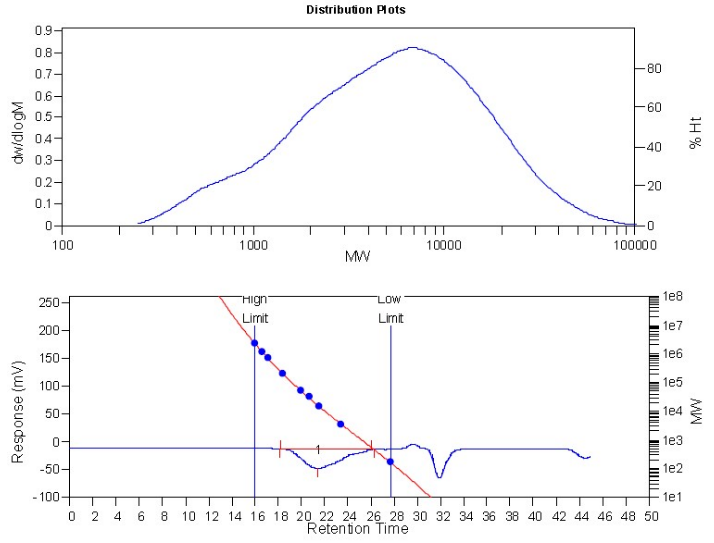

Cirrus GPC Version 3.4 Page 1 10/12/2016 4:51 PM

Supplementary Figure 31. GPC traces of the poly(ethylene-*co*-allyl alcohol) sample from Table 2, Entry 18.

MW Averages

Mp: 8918 Mn: 4369 Mv: 11890 Mw: 13975

Mz: 41759 Mz+1: 93740 PD: 3.1987

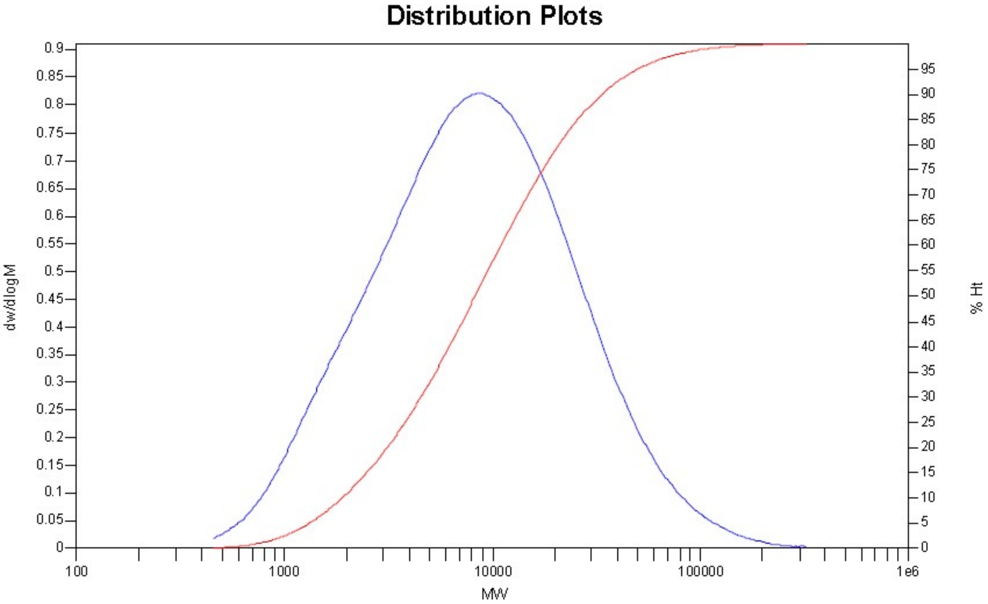

Supplementary Figure 32. GPC traces of the poly(ethylene-*co*-AME) sample from Table 2, Entry 19.

MW Averages

Mp: 14906 Mn: 4132 Mv: 17072 Mw: 20941

Mz: 78462 Mz+1: 182160 PD: 5.0680

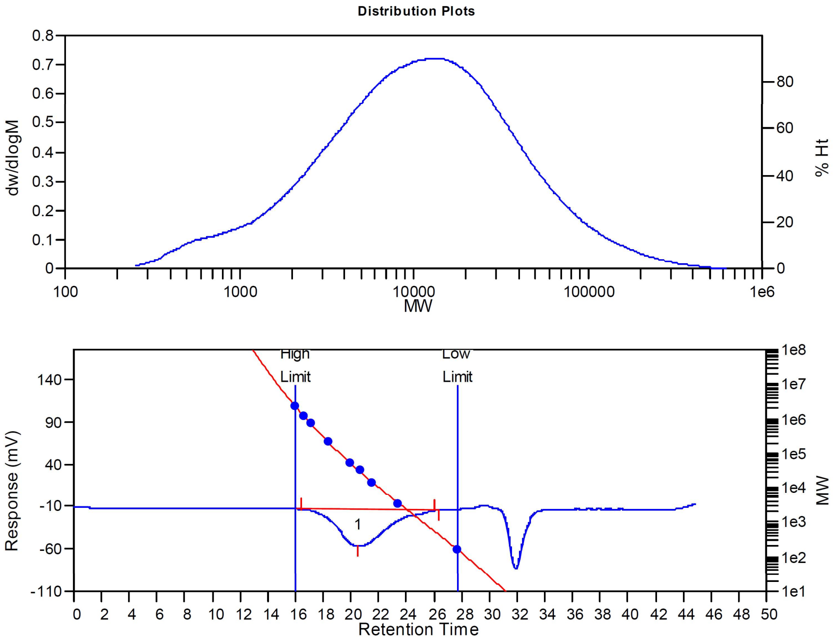

Supplementary Figure 33. GPC traces of the poly(ethylene-*co*-AA) sample from Table 2, Entry 20.

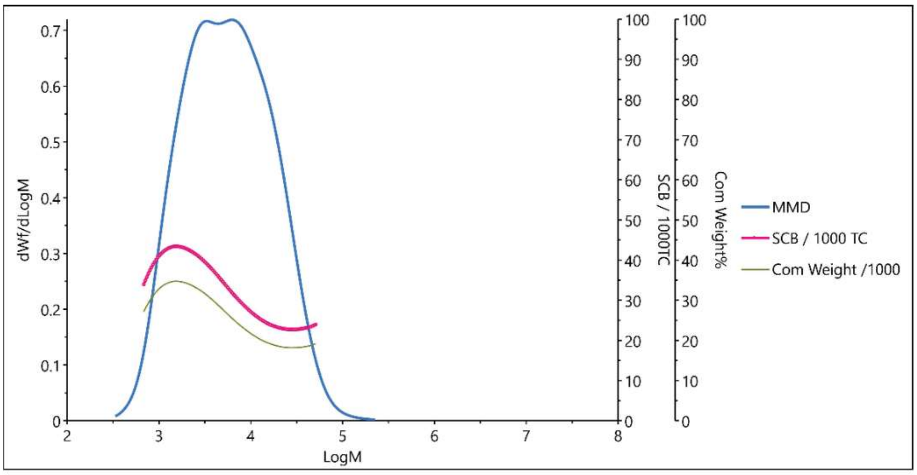

Results

Mw	9942	g/mol
Mn	3244	g/mol
Mw / Mn	3.06	
Mz	26866	g/mol
Mp	6199	g/mol
Mv	8368	g/mol
IVmwd	0.2614	dL/g
Bulk CH3 / 1000C	41.616	
Corrected Bulk CH3 / 1000C	32.8	CH3/1000TC corr

Calibrations

Calib Flow Marker	26.70	mL
Calib Mass constant	0.61	mL·V/mg

Performance

Flow Marker	26.81	mL
Mass recovery	102	%
Plate Count	20172	

Supplementary Figure 34. GPC traces of the poly(ethylene-*co*-AA) sample from Table 3, Entry 7.

a ATR-IR spectra of poly(ethylene-*co*-VA) sample from Table 2, Entry 1. (before methyl esterification)

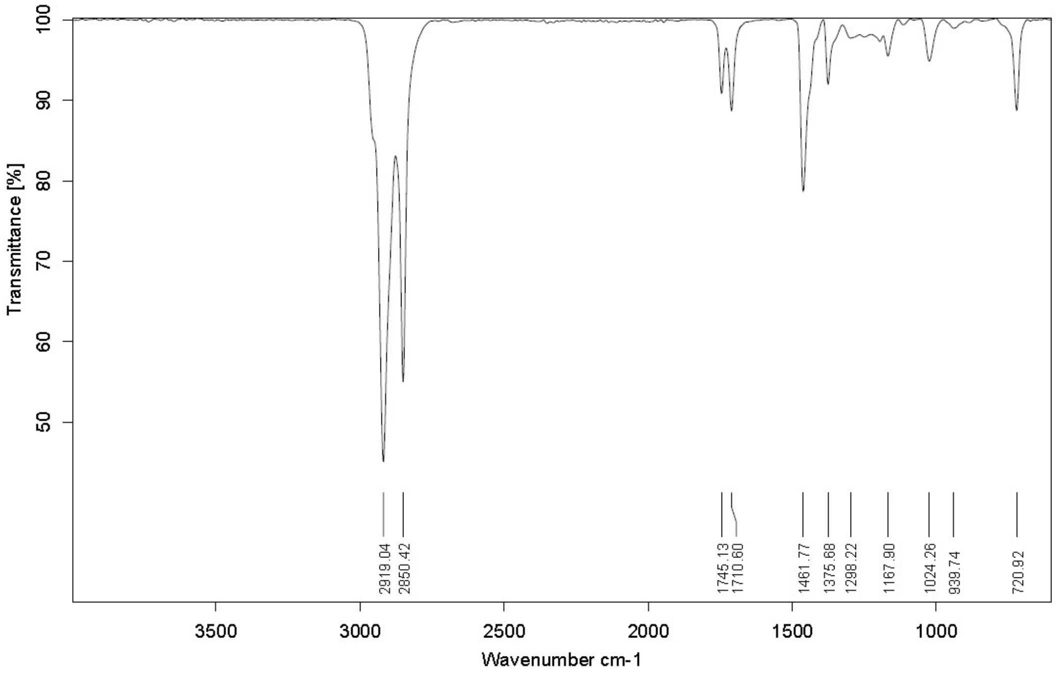

b ATR-IR spectra of poly(ethylene-*co*-VA) sample from Table 2, Entry 1. (after methyl esterification)

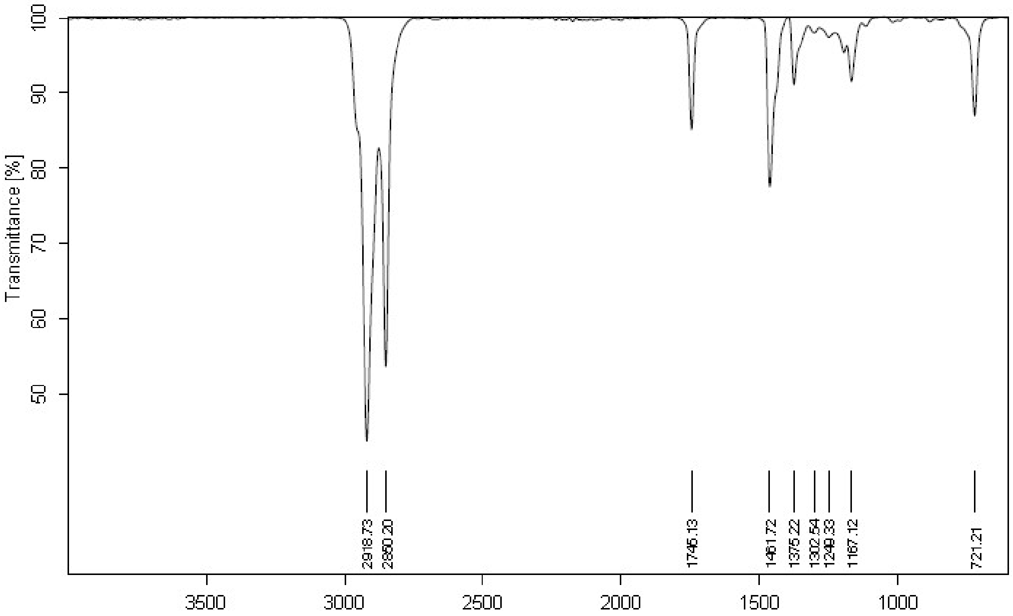

Supplementary Figure 35. ATR-IR spectra of poly(ethylene-*co*-VA) sample from Table 2, Entry 1 (2.1 mol% VA incorporation), (**a**) before methyl esterification (**b**) after methyl esterification.

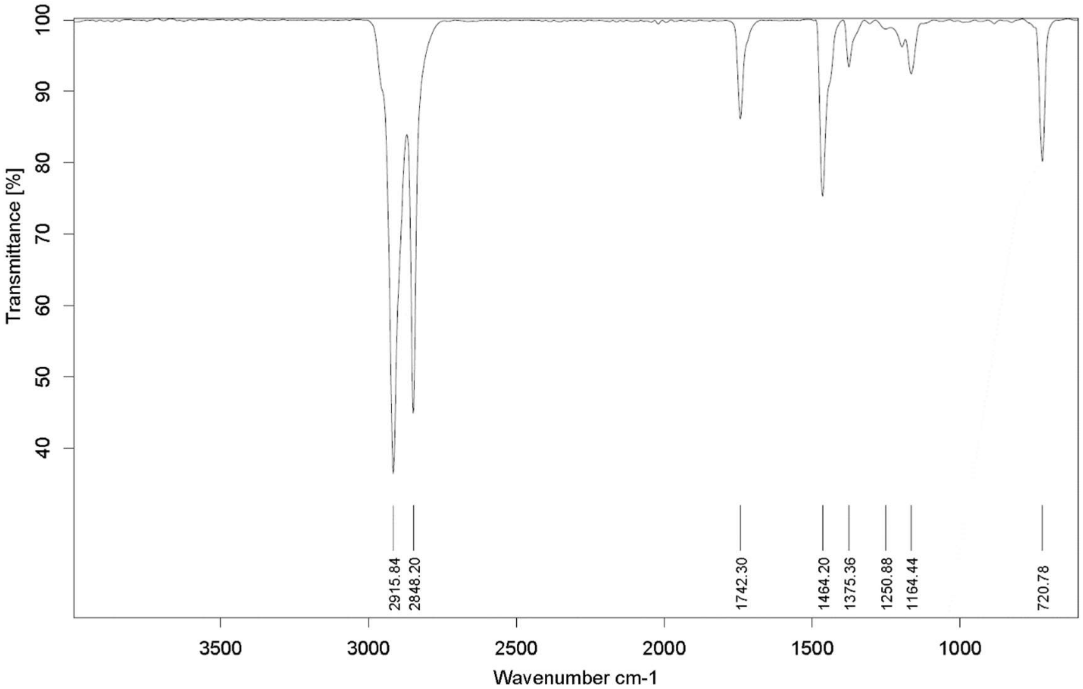

Supplementary Figure 36. ATR-IR spectra of poly(ethylene-*co*-AA) sample from Table 3, Entry 7 (2.1 mol% AA incorporation, after methyl esterification).

Supplementary References

1. Zhang, J.; Gao, H.; Ke, Z.; Bao, F.; Zhu, F.; Wu, Q. Investigation of 1-hexene isomerization and oligomerization catalyzed with β-diketiminato Ni(II) bromide complexes/methylaluminoxane system. *J. Mol. Catal. A: Chem.* **2005**, *231*, 27-34.

2. Budzelaar, P. M.; Moonen, N. P.; Gelder, R.; Smits, J. M.; Gal, A. Rhodium and Iridium β-diiminate complexes-olefin hydrogenation step by step. *Eur. J. Inorg. Chem.* **2000**, 753-769.

3. Xin, B. S.; Sato, N.; Tanna, A.; Oishi, Y.; Konishi, Y.; Shimizu, F. Nickel catalyzed copolymerization of ethylene and alkyl acrylates. *J. Am. Chem. Soc*. **2017,** *139*, 3611-3614.

4. Zhang, Y.; Mu, H.; Pan, L.; Wang, X.; Li, Y. Robust bulky [P,O] neutral nickel catalysts for copolymerization of ethylene with polar vinyl monomers. *ACS Catal.* **2018**, *8*, 596-5976.

5. Schmid, M.; Eberhardt, R.; Kukral, J.; Rieger, B. Novel non-symmetric Nickel-diimine complexes for the homopolymerization of ethene: control of branching by catalyst design. *Z. Naturforsch* **2002**, *57b*, 1141-1146.

6. Yokota, S.; Tachi, Y.; Itoh, S. Oxidative degradation of β-diketiminate ligand in Copper(II) and Zinc(II) complexes. *Inorg. Chem.* **2002**, *41*, 1342-1344.

7. Azoulay, J. D.; Rojas, R. S.; Serrano, A. V.; Ohtaki, H.; Galland, G. B.; Wu, G.; Bazan, G. C. Nickel α-keto-β-diimine initiators for olefin polymerization. *Angew. Chem. Int. Ed.* **2009**, *48*, 1089-1092.

8. Zhang, J. -T.; Wang, H. -Y.; Zhu, W.; Cai, T. -T.; Guo, Y. -L. Solvent-assisted electrospray ionization for direct analysis of various compounds (complex) from low/nonpolar solvents and eluents. *Anal. Chem.* **2014**, *86*, 8937-8942.

9. Chen, E. Y.-X.; Marks, T. J. Cocatalysts for metal-catalyzed olefin polymerization: activators, activation processes, and structure-activity relationships. *Chem. Rev*. **2000,** *100*, 1391-1434.

10. Schröder, D. Ion-mobility mass spectrometry of complexes of nickel and acetonitrile. *Collect. Czech. Chem. Commun.* **2011**, *76*, 351-369.

11. Glasovac, Z.; Štrukil, V.; Eckert-Maksić, M.; Schröder, D.; Schlangen, M.; Schwarz, H. Coordination chemistry of Nickel(II) nitrate with superbasic guanidines as studied by electrospray Mass Spectrometry. *Int. J. Mass Spectrom.* **2010**, *290*, 22-31.

12. Tsierkezos, N. G.; Schröder, D.; Schwarz, H. Complexation of Nickel(II) by ethylenediamine investigated by means of Electrospray Ionization Mass Spectrometry. *Int. J. Mass Spectrom.* **2004**, *235*, 33-42.

13. Tsierkezos, N. G.; Schröder, D.; Schwarz, H. Gas-phase solvation behavior of Ni(II) in water/N,N-dimethylformamide mixtures. *J. Phys. Chem. A* **2003**, *107*, 9575-9581.

14. Compared with Chen's reported work, structure III in quantitative ^{13}C NMR spectrum is consistent with E/10-methyl undecanoate copolymers. Dai, S.; Li, S.; Xu, G.; Chen. C. Direct synthesis of polar functionalized polyethylene thermoplastic elastomer. *Macromolecules* **2020**, 53, 2539−2546.

15. Wucher, P.; Caporaso, L.; Roesle, P.; Ragone, F.; Cavallo, L.; Mecking, S.; Göttker-Schnetmann, I. Breaking the regioselectivity rule for acrylate insertion in the Mizoroki–Heck reaction. *Proc. Natl Acad. Sci. USA.* **2011**, *108*, 8955-8959.

16. Stephenson, C. J.; McInnis, J. P.; Chen, C.; Weberski, M. P.; Motta, A.; Delferro, M.; Marks, T. J. Ni(II) phenoxyiminato olefin polymerization catalysis: striking coordinative modulation of hyperbranched polymer microstructure and stability by a proximate sulfonyl group. *ACS Catal.* **2014**, *4*, 999-1003.
17. Wiedemann, T.; Voit, G.; Tchernook, A.; Roesle, P.; Gottker-Schnetmann, I.; Mecking, S. Monofunctional hyperbranched ethylene oligomers. *J. Am. Chem. Soc.* **2014**, *136*, 2078-2085.
18. Compared with our previous work, structure III in quantitative ^{13}C NMR spectrum is consistent with E/9-decen-1-ol copolymers which represents polar monomer branch with more than three carbon linkage. Yang, X. -H.; Liu, C,-R.; Wang, C.; Sun, X.-L.; Guo, Y-H.; Wang, X-K.; Wang, Z.; Xie, Z.; Tang, Y. [O-NSR]TiCl3-catalyzed copolymerization of ethylene with functionalized olefins. *Angew. Chem., Int. Ed.* **2009**, 48, 8099–8102.
19. Galland, G. B.; de Souza, R. F.; Mauler, R. S.; Nunes, F. F. ^{13}C NMR Determination of the composition of linear low-density polyethylene obtained with [η^3-Methallyl-nickel-diimine]PF_6 complex. *Macromolecules* **1999**, *32*, 1620-1625.
20. Konishi, Y.; Tao, W.-J.; Yasuda, H.; Ito, S.; Oishi, Y.; Ohtaki, H.; Tanna, A.; Tayano, T.; Nozaki, K. Nickel-catalyzed propylene/polar Monomer copolymerization. *ACS Macro Lett.* **2018,** *7*, 213-217.
21. Frisch, M. J.; Trucks, G. W.; Schlegel, H. B.; Scuseria, G. E.; Robb, M. A.; Cheeseman, J. R.; Scalmani, G.; Barone, V.; Petersson, G. A.; Nakatsuji, H.; Li, X.; Caricato, M.; Marenich, A. V.; Bloino, J.; Janesko, B. G.; Gomperts, R.; Mennucci, B.; Hratchian, H. P.; Ortiz, J. V.; Izmaylov, A. F.; Sonnenberg, J. L.; Williams; Ding, F.; Lipparini, F.; Egidi, F.; Goings, J.; Peng, B.; Petrone, A.; Henderson, T.; Ranasinghe, D.; Zakrzewski, V. G.; Gao, J.; Rega, N.; Zheng, G.; Liang, W.; Hada, M.; Ehara, M.; Toyota, K.; Fukuda, R.; Hasegawa, J.; Ishida, M.; Nakajima, T.; Honda, Y.; Kitao, O.; Nakai, H.; Vreven, T.; Throssell, K.; Montgomery Jr., J. A.; Peralta, J. E.; Ogliaro, F.; Bearpark, M. J.; Heyd, J. J.; Brothers, E. N.; Kudin, K. N.; Staroverov, V. N.; Keith, T. A.; Kobayashi, R.; Normand, J.; Raghavachari, K.; Rendell, A. P.; Burant, J. C.; Iyengar, S. S.; Tomasi, J.; Cossi, M.; Millam, J. M.; Klene, M.; Adamo, C.; Cammi, R.; Ochterski, J. W.; Martin, R. L.; Morokuma, K.; Farkas, O.; Foresman, J. B.; Fox, D. J. *Gaussian 16 Rev. A.03*, Wallingford, CT, 2016.
22. Becke, A. D. A new mixing of Hartree-Fock and local density-functional theories. *J. Chem. Phys.* **1993**, *98*, 1372-1377.
23. Becke, A. D. Density-functional exchange-energy approximation with correct asymptotic behavior. *Phys. Rev. A* **1988**, *38*, 3098-3100.
24. Lee, C.; Yang, W.; Parr, R. G. Development of the Colle-Salvetti correlation-energy formula into a functional of the electron density. *Phys. Rev. B* **1988**, *37*, 785-789.
25. Hay, P. J.; Wadt, W. R. *Ab initio* effective core potentials for molecular calculations. Potentials for the transition metal atoms Sc to Hg. *J. Chem. Phys*. **1985**, *82*, 270-283.
26. Hay, P. J.; Wadt, W. R. *Ab initio* effective core potentials for molecular calculations. Potentials for K to Au including the outermost core orbitals. *J. Chem. Phys.* **1985**, *82*, 299-310.
27. Wadt, W. R.; Hay, P. J., *Ab initio* effective core potentials for molecular calculations. Potentials for main group elements Na to Bi. *J. Chem. Phys.* **1985,** *82*, 284-298.
28. Zhao, Y.; Truhlar, D. G. The M06 Suite of Density functionals for main group thermochemistry, thermochemical kinetics, noncovalent interactions, excited states, and transition elements: two new

functionals and systematic testing of four M06-class functionals and 12 other functionals. *Theor. Chem. Acc.* **2008**, *120*, 215-241.
29. Zhao, Y.; Truhlar, D. G. Benchmark energetic data in a model system for Grubbs II metathesis catalysis and their use for the development, assessment, and validation of electronic structure methods. *J. Chem. Theory Comput.* **2009**, 5, 324-333.
30. Marenich, A. V.; Cramer, C. J.; Truhlar, D. G. Performance of SM6, SM8, and SMD on the SAMPL1 test set for the prediction of small-molecule solvation free energies. *J. Phys. Chem. B* **2009**, *113*, 4538-4543.
31. Kaupp, M.; Schleyer, P. v. R.; Stoll, H.; Preuss, H. Pseudopotential approaches to Ca, Sr, and Ba hydrides. Why are some alkaline earth MX_2 compounds bent? *J. Chem. Phys.* **1991**, *94*, 1360-1366.
32. Henglein, A. Physicochemical properties of small metal particles in solution: "Microelectrode" reactions, chemisorption, composite metal particles, and the atom-to-metal transition. *J. Phys. Chem.* **1993**, *97*, 5457-5471.
33. Steinbrenner, U.; Bergner, A.; Dolg, M.; Stoll, H. On the transferability of energy adjusted pseudopotentiais: A calibration study for XH_4 (X=C, Si, Ge, Sn, Pb). *Mol. Phys.* **1994**, *82*, 3-11.
34. Szabo, M. J.; Galea, N. M.; Michalak, A.; Yang, S.-Y.; Groux, L. F.; Piers, W. E.; Ziegler, T. Copolymerization of ethylene with polar monomers: chain propagation and side reactions. A DFT theoretical study using zwitterionic Ni(II) and Pd(II) catalysts. *J. Am. Chem. Soc.* **2005**, *127*, 14692-14703.
35. Yang, S.-Y.; Szabo, M. J.; Michalak, A.; Weiss, T.; Piers, W. E.; Jordan, R. F.; Ziegler, T. The exploration of neutral azoligand-based Grubbs type Palladium(II) complex as potential catalyst for the copolymerization of ethylene with acrylonitrile: a theoretical study based on density functional theory. *Organometallics* **2005**, *24*, 1242-1251.
